# Neurodegenerative and Neurodevelopmental Diseases and the Gut-Brain Axis: The Potential of Therapeutic Targeting of the Microbiome

**DOI:** 10.3390/ijms24119577

**Published:** 2023-05-31

**Authors:** Brian Bicknell, Ann Liebert, Thomas Borody, Geoffrey Herkes, Craig McLachlan, Hosen Kiat

**Affiliations:** 1 NICM Health Research Institute, University of Western Sydney, Westmead, NSW 2145, Australia; ann.liebert@sydney.edu.au (A.L.); hosen.kiat@chi.org.au (H.K.); 2 Faculty of Medicine and Health, University of Sydney, Camperdown, NSW 2006, Australia; 3 Department of Governance and Research, Sydney Adventist Hospital, Wahroonga, NSW 2076, Australia; geoffrey.herkes@sah.org.au; 4 Centre for Digestive Diseases, Five Dock, NSW 2046, Australia; thomas.borody@cdd.com.au; 5 Centre for Healthy Futures, Torrens University Australia, Ultimo, NSW 2007, Australia; reperfusion@hotmail.com; 6 Macquarie Medical School, Macquarie University, Macquarie Park, NSW 2109, Australia; 7 ANU College of Health and Medicine, Australian National University, Canberra, ACT 2601, Australia

**Keywords:** microbiome, photobiomodulation, faecal microbiome transplants, neurodegenerative disease, neurodevelopmental disease, Alzheimer’s disease, Parkinson’s disease, autism spectrum disorder

## Abstract

The human gut microbiome contains the largest number of bacteria in the body and has the potential to greatly influence metabolism, not only locally but also systemically. There is an established link between a healthy, balanced, and diverse microbiome and overall health. When the gut microbiome becomes unbalanced (dysbiosis) through dietary changes, medication use, lifestyle choices, environmental factors, and ageing, this has a profound effect on our health and is linked to many diseases, including lifestyle diseases, metabolic diseases, inflammatory diseases, and neurological diseases. While this link in humans is largely an association of dysbiosis with disease, in animal models, a causative link can be demonstrated. The link between the gut and the brain is particularly important in maintaining brain health, with a strong association between dysbiosis in the gut and neurodegenerative and neurodevelopmental diseases. This link suggests not only that the gut microbiota composition can be used to make an early diagnosis of neurodegenerative and neurodevelopmental diseases but also that modifying the gut microbiome to influence the microbiome–gut–brain axis might present a therapeutic target for diseases that have proved intractable, with the aim of altering the trajectory of neurodegenerative and neurodevelopmental diseases such as Alzheimer’s disease, Parkinson’s disease, multiple sclerosis, autism spectrum disorder, and attention-deficit hyperactivity disorder, among others. There is also a microbiome–gut–brain link to other potentially reversible neurological diseases, such as migraine, post-operative cognitive dysfunction, and long COVID, which might be considered models of therapy for neurodegenerative disease. The role of traditional methods in altering the microbiome, as well as newer, more novel treatments such as faecal microbiome transplants and photobiomodulation, are discussed.

## 1. Preamble

The human microbiome exists wherever the skin or a mucous membrane comes into contact with the outside world, allowing microorganisms (the microbiota) to colonise these surfaces. The microbiome is the sum of the genes in the microbiota, which contribute to our own genetics. The microbiome is acquired at birth (or perhaps before [1]), develops with age, and is modified throughout life. Distinct microbiomes develop in the oral cavity, nasopharyngeal airway, respiratory tract, urogenital tract, and various skin sites, and evidence is accumulating that sites that have been previously considered sterile may have their own distinct microbiomes, such as the liver [2], blood [3], and even the brain [4]. The composition, density, and diversity of our various microbiomes are dynamic and influenced by a myriad of factors, including genetics, geography, lifestyle, diet, the immune system, history of infections, and a plethora of environmental elements. The gastrointestinal tract (GIT) has a large interface between the outside environment and the body and has by far the largest and most complex microbiome. It is arguably the most important microbiome in terms of its interaction with the rest of the body, sometimes being considered an organ in its own right [5]. The microbiome of the GIT varies greatly along the length of the gastrointestinal tract. While there are perhaps 1 billion microbial cells per mL of saliva, this number is reduced to perhaps thousands per mL in the high acidity of the stomach, with numbers increasing again in the ileum with the decrease in acidity and slowing peristalsis. The numbers reach their highest levels and most dense concentration in the colon, in part due to the slowing GIT movement and undigested nutrients, with up to 10^11^ bacteria per gram of faeces. It is also the colon (gut) microbiome that has the most influence on the body. It consists of up to 100 trillion bacteria, archaea, protists, and fungi that colonise the lumen of the colon, as well as the intestinal walls and the mucus, with the majority of the cells being bacteria [6]. Interestingly, there may be as many virus particles as bacteria in the gut [7], with most being bacteriophages with bacterial hosts. The number of bacteria rivals or exceeds the number of cells composing our body. More importantly, the gut microbiome contains, by some estimates [8,9], over 22,000,000 genes, while our own genome includes just over 23,000 genes [10]. Thus, there are perhaps 1000 times more genes contributed by the gut microbiome than by our own cells. These genes are active, and many are complementary to our bodily processes, with the microbiome having co-evolved with humans, as well as our mammalian and other predecessors. The genes contribute to our holobiome, the complement of transcribable genes from our own genome plus the microbial genome. The gut microbiome contributes to the digestion of food, nutrition, metabolism, immunity and immune tolerance, and protection from invading pathogens. This mutualistic relationship between the gut microbes and our body relies on a complex network of molecular communication, the interpretation of which is still in its infancy.

The gut microbiome consists of eight major bacterial phyla, of which 90% is normally a combination of Bacteroidetes and Firmicutes, with smaller numbers of Actinobacteria, Proteobacteria, Fusobacteria, and Verrucomicrobia. There are hundreds of separate genera that can be found in the microbiome, most probably with thousands of separate species and strains. The majority of the Firmicutes belong to various *Clostridium* genera; the Bacteroidetes are predominantly from the *Bacteroides* and *Prevotella* genera; most bacteria in the Actinomycetes are from the *Bifidobacterium* genus; and Verrucomicrobia is represented by a single genus, *Akkermansia.* The totality and balance of the microbiome play a role in keeping our gut and metabolism healthy. It is increasingly apparent that the microbiome plays a critical role in human health and disease, with multiple roles in our metabolism, including the production of neurotransmitters, hormones, and other bioactive molecules, and in maintaining the integrity of the intestinal barrier, the blood–brain barrier (BBB), and in the regulation of the immune system. An unbalanced microbiome (dysbiosis) is linked to many diseases, including diseases of neurodegeneration and neurodevelopment.

## 2. Technology

It is only in the past few decades that technology has advanced beyond culture and microscopy to the point where microbiome analysis is currently feasible and economical. DNA is extracted from (usually) faecal material, and this is used in next-generation sequencing (NGS), most often the sequencing of the 16SrRNA gene, which is a conserved region of DNA that is common to (almost) all bacteria and archaea but also has variable regions that are particular to different taxonomic levels. This allows the identification of bacteria when the sequence is compared to an ever-expanding database of bacteria. 16S rRNA analysis provides a relatively low level of taxonomic classification, only reliably to the genus level. The other major technique is whole-genome sequencing or metagenomics, where the entire DNA of the microbiome is sequenced. This allows a higher level of classification to the species/strain level, as well as information on non-bacterial microbes and functional genes. This technique is more expensive and requires more computational power and expertise to analyse. Other techniques include metatranscriptomics (analysis of mRNA to determine functional genes in the microbiome), metaproteomics or community proteomics (analysis of the collective proteins present in the microbiome), and metabolomics (analysis of the metabolic products that are present).

Microbiome analysis is carried out from faecal samples in most studies, but this will not capture the proportion of the microbiome more tightly attached to or embedded in an extracellular matrix (biofilm) [11]. There is some evidence that the use of only stool samples to assess the microbiome will miss much microbial biodiversity and bias the interpretation of the gut microbiome [12].

## 3. Microbiome Development

The gut microbiome begins to develop immediately after birth and is affected by the delivery method (natural birth versus caesarean), gestational age (pre-term versus full term), infant feeding choices (breast- versus bottle-fed), solid food introduction, transition to cow’s milk, increased food variety, and changes in diet through the teenage and adult years [13,14,15]. While the microbiome is relatively stable by age 3, its structure is continually modified by numerous factors, such as hormonal changes, diet, and the environment. Progression to old age (generally from 65 years) is accompanied by a profound change in the microbiome, with reduced diversity and high variation in the microbiome structure [16].

There is great diversity in the microbiome composition from person to person, with only a small proportion of the gut microbiota being shared in a population (perhaps less than one-third of the genera), with the majority of the genera being specific to an individual [17]. Even closely related individuals, such as identical twins, have distinct microbiomes modulated by protean environmental factors [18].

## 4. A Healthy Microbiome?

There is still much controversy as to what constitutes a “healthy” microbiome, and, in many ways, the knowledge of the microbiome structure is still in its infancy. Combined global data [19] have indicated that there may be a “core” microbiota consisting of 14 genera, but with probably hundreds of other genera still to be investigated. A list of genera from the microbiomes of healthy people has identified 155 bacterial and 2 archaeal organisms, with 84 potentially representing the core microbiota [20]. It is widely acknowledged that the balance of the microbiome appears to be most important for health, with diversity (most often calculated as α-diversity [21]) being an important factor and with the disruption of this balance being associated with disease.

The Firmicutes-Bacteroidetes ratio has been suggested as a proxy for human gut health, with the ratio being higher in an ageing microbiome [22] as well as in obesity, metabolic syndrome, and type 2 diabetes myelitis (T2DM) [23]. This is not universally accepted, however [24], with an increased ratio not consistently found in a number of diseases associated with a dysregulated microbiome. Another suggested proxy for gut health is the microbiome enterotype, where a microbiome is assigned to one of three clusters, which are established by long-term diet and identified by the variation in one of three genera (enterotype 1—*Bacteroides*; enterotype 2—*Prevotella*; enterotype 3—*Ruminococcus*). The helpfulness of this construct has been questioned [25], with microbiome variations being visualised as a continuum rather than rigid groups.

**Figure 1:**
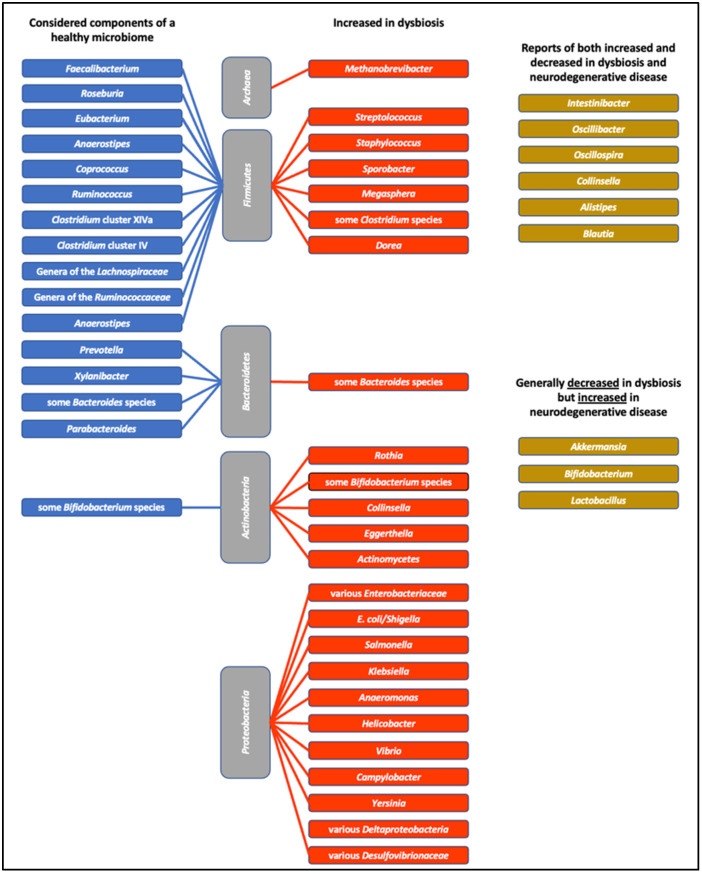
Putative “healthy” and “unhealthy” bacteria found in a balanced microbiome and dysbiosis, respectively.

While it is difficult to classify bacteria as either “good” or “bad”, a list of bacteria can be produced (see Figure 1) depending on the metabolic products that are produced by the microbes and whether the bacterium is associated with health or with a disease state [26]. In general, bacteria associated with a healthy microbiome include those that produce short-chain fatty acids (SCFAs), and those that are considered negative in the microbiome are potential pathogens and/or those bacteria that produce bacterial toxins such as lipopolysaccharide (LPS). It is important to remember that a healthy microbiome is associated with high microbiota diversity and a balance of bacteria. Bacteria that are associated with various diseases can be found, usually in low numbers, in a healthy microbiome [20]. A recent study of almost 3500 people [27] found that a number of what are considered to be beneficial bacterial genera were negatively correlated with each other (*Bacteroides* versus *Prevotella*; Lachnospiraceae versus Ruminococcaceae; *Bifidobacterium* versus *Faecalibacterium)*, suggesting that direct competition between bacteria with similar nutritional needs can result in the predominance of one over the other, leading to a spectrum of healthy microbiomes. Thus, a healthy, well-functioning microbiome does not depend on one specific combination of bacteria.

### 4.1. Influences on the Microbiome

Diet, unsurprisingly, has a major influence on microbiome structure, with evidence strongly suggesting that it is long-term diet that has the most influence on an individual’s microbiome [28]. The gut microbiome ferments some of the dietary fibre (polysaccharides, oligosaccharides, resistant starches, fermentable carbohydrates), that passes through to the colon undigested by the body’s limited range of digestive enzymes. In addition, small amounts of protein (5–10% or more, depending on the diet) can pass into the colon to be used as a substrate for proteolytic bacteria. The other main source of nutrition for gut microbes is mucus produced by goblet cells.

Gut microorganisms produce secondary metabolites that actively contribute to our body’s homeostasis in a mutualistic relationship between human and microbial cells. The body’s metabolism and the metabolism of the microbiota of the gut have co-evolved over millennia to produce a web of complex interactions that are far from being fully understood. Recent historical changes in the human diet affect the substrates that are available for the gut microbiome, which changes the microbial community structure and in turn affects the production of beneficial metabolites. The change from a hunter–gatherer diet to a diet typified by increased grains and finally to the more recent global dietary pattern that typifies a “Western” diet has resulted in shifts to the gut microbiome. The widespread adoption of a Western diet, with its abundance of energy-dense foods, simple carbohydrates (sugars), fats, and protein and low intake of fruits and vegetables, has resulted in a diet low in plant-derived fibre. This negatively impacts the microbial balance, since many bacteria in the colon require fermentable, undigested carbohydrates. The resultant change in microbial composition impacts the host’s physiology, metabolism, and immunity and has been implicated in many metabolic and chronic diseases, including metabolic syndrome, T2DM, cardiovascular disease (CVD), cancers, and increased mortality [29].

There is a dramatic difference between the microbiota of people with a Western diet and those of rural populations that consume a diet richer in fibre. Children from rural Africa have higher proportions of Bacteroidetes and lower Firmicutes than children from countries with a Western diet, with increases in *Prevotella* and *Xylanibacter* genera [30]. Geography can be one factor that might influence the microbiome structure, although this may be mostly due to dietary differences. For example, in a study of inflammatory bowel disease (IBD) patients, geography was a major factor in microbiome variation [31], although most variation was still unexplained. A change in a person’s geography influences microbiome structure. Immigration to the USA results in significant changes in the microbiome (in addition to increased generational obesity) [32], most probably due to dietary changes.

Diets high in legumes, fish, and nuts are associated with a balanced microbiome with few opportunistic pathogens and increased SCFA-producing bacteria (*Roseburia*, *Faecalibacterium*, and *Eubacterium*) [33]. One diet that incorporates these types of foods is the Mediterranean diet, known to be protective against a range of diseases, including obesity, hypertension, and CVD [34], as well as Alzheimer’s disease [35,36]. The microbiome of people who consume Mediterranean diets are generally enriched in *Prevotella* and reduced in Firmicutes [37]. Similarly, a vegetarian or vegan diet, with its greater reliance on plant protein and higher fibre content, generally results in increased Bacteroidetes genera compared to a meat-based diet [38], with increased numbers of *Ruminococcus*, *Eubacterium*, *Roseburia*, *Bifidobacterium*, and *Lactobacillus* and increased SCFA production, but with lower levels of *Bacteroides* and Enterobacteriaceae genera. Wholegrain diets (high in carbohydrates and fibre) tend to produce microbiomes with less Enterobacteriaceae and Desulfovibrionaceae and increased Bifidobacteriaceae and *Prevotella* [17]. A diet low in fermentable oligosaccharides, disaccharides, monosaccharides and polyols (FODMAP diet) does not change the α-diversity or β-diversity but appears to reduce *Bifidobacterium* species [39] and improves intestinal permeability in patients with diarrhoea-predominant irritable bowel syndrome (IBS).

Individual foods can also correlate with specific inflammatory patterns and bacteria [33]. For example, coffee, tea, and berries encourage *Oscillibacter* as well as anti-inflammatory pathways; red wine can induce beneficial changes in the microbiome [40]; the consumption of fish is associated with SCFA production [41]; and the consumption of chocolate is associated with changes in the microbiome and SCFA increases [42,43].

A diet high in fibre is associated with lower markers of inflammation [44] and a lower incidence of chronic inflammatory diseases [45,46]. A diet low in fibre disturbs the balance of the microbiome and is associated with a wide range of metabolic diseases in the host [47,48]. The mix of beneficial bacteria, rather than specific genera, is associated with high levels of SCFA production [27]. Dietary intervention by increasing fibre intake can reduce the incidence of inflammatory diseases and improve the microbiome [49], although a large prospective study of over 56,000 Danes over 50 years did not support this, with long-term high fibre intake not significantly reducing the risk of late-onset chronic inflammatory diseases [50].

A diet high in protein results in an increase in protein reaching the colon and subsequent increases in protein-fermenting bacteria, resulting in less SCFA production, an increasing pH, and a subsequent shift in the microbial population [51]. The changed microbiome can increase potentially toxic metabolites, including ammonia, phenols, indoles, amines, sulphides, and *N*-nitroso compounds (see below), which are associated with an increased risk of IBD, T2DM, colon cancer, CVD, and neurodegenerative diseases [52,53]. Similarly, a diet high in fat is also associated with an altered microbiome, along with the development of obesity and chronic diseases. High-fat diets reduce microbiome diversity, with decreased *Faecalibacterium* and increased *Alistipes*, together with increases in potentially toxic metabolites and pro-inflammatory markers [54,55]. High sugar intake also disrupts the microbiome, resulting in reduced diversity, a decrease in Bacteroidetes, and an increase in the pro-inflammatory Proteobacteria, specifically the LPS-producing Enterobacteriaceae [56]. Hogh sugar diets are linked to obesity, metabolic disorders, and CVD (see below).

An apparent anomaly to this trend is the ketogenic diet, which has been shown to be effective in reducing seizures, improving metabolic health, reducing obesity, improving insulin resistance and dyslipidaemia. This has been trialled for autism spectrum disorder (ASD) and Alzheimer’s disease (AD). While there have been few studies to show the effects of this diet on the human microbiome, a mouse study [57] demonstrated decreased microbiome diversity but increases in *Akkermansia* and *Parabacteroides.* A single pilot study of the effects of a ketogenic diet for obesity found a decrease in Firmicutes and an increase in Bacteroidetes, together with a decrease in body mass index and improved insulin resistance [58].

Exercise has also been shown to positively influence the microbiome in animal models and in humans [21].

### 4.2. Contribution to Health—SCFA Production

A healthy and balanced microbiome has a major influence on overall health, including metabolising nutrients that reach the colon, maintaining a strong barrier between the gut contents and the tissues of the body, degrading potential toxins, producing vitamins and other metabolites, protecting the body from pathogens that find their way into the gut, and stimulating and modulating the immune system.

One of the major ways that the microbiome contributes to gut and overall health is through the production of SCFAs from the fermentation of soluble fibres. SCFAs consist of more than 95% acetate, butyrate, and propionate (usually with a 60:20:20 ratio) and smaller amounts of formate, valerate, and caproate and the branched-chain fatty acids isobutyrate, 2-methyl butyrate, and isovalerate. The ratios depend on the mix of bacteria in the colon. All of these molecules have physiological effects. Butyrate is considered the most important SCFA and is produced by a wide phylogenetic range of bacteria, including many types of Firmicutes (e.g., *Ruminococcus*, *Clostridial* Clusters IV and XIVa, *Eubacterium*, *Anaerostipes*, *Coprococcus*, *Faecalibacterium*, and *Roseburia* genera) as well as some *Bacteroides* species and some *Bifidobacterium* species [17]. Butyrate is the major energy source for epithelial cells that line the large intestine. Adequate SCFAs ensure the health of the cells and regulates the proteins (claudin-1, occluding, and zonula occludens-1) in the tight junctions between the cells and so maintains the barrier between the contents of the colon and the tissues of the body. This prevents bacteria and their toxic metabolites, such as LPS, from entering the bloodstream and causing inflammation. Butyrate stimulates serotonin (5-HT) production in the enteroendocrine cells (EECs) of the epithelium and stimulates mucin production by goblet cells for the protective mucous layer. Butyrate can also downregulate intestinal inflammation by regulating inflammation pathways such as G protein-coupled receptors, nuclear factor kappa light chain enhancer of activated B cells (NF-κB), and Janus kinase/signal transducers and activators of transcription (JAK/STAT), and can modulate pro-inflammatory cytokine release (interleukin 1 beta (IL-1β), IL-12, tumour necrosis factor alpha (TNF-α)), and upregulating anti-inflammatory IL-10 [59,60]. Butyrate is also circulated via the lymphatic and systemic circulation, affecting the BBB and other organ systems.

Bacteroidetes produce mostly acetate and propionate, as well as some butyrate [17]. Propionate and acetate cross the intestinal barrier, and propionate is metabolised by the liver, mostly for gluconeogenesis, while acetate circulates in the blood to be taken up by cells for lipid biosynthesis. SCFAs also have various roles in gastric mucosal cells and in the immune and oxidative stress responses [61]. Acetate and butyrate can stimulate EECs to release glucagon-like peptide-1 (GLP-1) and peptide-YY (PYY) [17] to reduce appetite and stimulate insulin release.

### 4.3. Contribution to Health—Other Metabolites

Polyphenols and carotenoids are a group of diverse compounds found in plants (fruit, vegetables, seeds, cereals, tea, coffee, wine), including flavonoids, phenolic acids (such as lignin), and polyphenol amides (such as capsinoids). Most pass undigested into the colon, where they are modified by the microbiome and then absorbed as bioactive compounds, being anti-inflammatory and antioxidant in nature and potentially having beneficial effects on a number of inflammatory diseases [62].

Bile acids (cholic acid and chenodeoxycholic acid) are produced from cholesterol in the liver, secreted into the duodenum to aid in the digestion of fats, and substantially reabsorbed in the small intestine. The small amounts of bile acids that reach the colon are metabolised and converted to secondary bile acids by colon microbiota. Secondary bile acids target G protein-coupled bile acid receptor 1 and regulate glucose, cholesterol, and energy homeostasis [17], help to maintain gut integrity [17,63], inhibit NF-κB-dependent transcription of pro-inflammatory genes [17], downregulate the inflammatory response of macrophages and natural killer T cells [64], and suppress *C. difficile* germination and growth [65].

A variety of vitamins, such as vitamins B9, B1, B2, B3, and K, are produced by bacteria in the microbiome, including some *Bifidobacterium*, *Bacillus*, and Bacteroidetes species [17].

In addition, microbes in the gut contribute a multitude of metabolites that are recognised by the body, with profound effects on metabolism. These include metabolites that are chemically and biologically identical to metabolites produced by the body (e.g., glutamate, gamma-aminobutyric acid—GABA, dopamine, and acetylcholine) and analogues or functional mimics of metabolites (e.g., catecholamines, α-MSH, and tryptophan precursors). The microbiome influences the synthesis of neurotransmitters in the body’s metabolic pathways and stimulates the production of metabolites by EECs, such as 5-HT, epinephrine, and dopamine. Supplementation with *Akkermansia* in mice has been shown to increase 5-HT production in enterochromaffin cells [66]. Metabolites can act locally on intestinal mucosal cells or the enteric nervous system (ENS), act on the brain via the vagus nerve, or enter the circulation. Metabolites can also have a direct effect on metabolomics, such as glutathione metabolism, amino acid and fatty acid metabolism, and redox balance [67].

Changes in the microbiome composition influence tryptophan and 5-HT levels as well as key brain metabolites, such as dopamine, norepinephrine, and brain-derived neurotrophic factor (BDNF) [68]. The majority tryptophan, an essential amino acid, is absorbed in the small intestine, where it is either involved in the kynurenine pathway with the end product being nicotinamide adenine dinucleotide (NAD), or acts as a precursor for 5-HT production [69]. One rate-limiting step in the production of kynurenine is indoleamine 2,3-dioxygenase 1, which is both controlled by the gut microbiome and also a modulator of the microbiome [62]. Some tryptophan passes through to the colon, where bacterial degradation produces a range of secondary indole metabolites (such as tryptamine, indoleethanol, indolealdehyde, indoleacetic acid, indolepropionic acid, and indoleacrylic acid) [70]. These act as signals to enterochromaffin cells for 5-HT production [71] and positively modulate innate immunity and systemic inflammation [72].

Indole compounds have multiple functions, such as modulating GLP-1, insulin, and 5-HT secretion, improving intestinal integrity, and modulating immunity [62]. *Lactobacillus reuteri* has been shown to produce the metabolite indole-3-carbinol, which modulates regulatory T-cell homeostasis to give tolerance to food antigens and intestinal microbes [73].

### 4.4. The Microbiome and Immunity

The gut is a major interface between the body and the external world, and the mucosa comes into contact with myriad compounds, ranging from the breakdown products of food, fibres, and other undigested substances to toxins, drugs, and invading organisms. The potential exposure of the vast numbers of bacteria (both commensal and pathogenic), archaea, fungi, protists, viruses, and compounds from the colon to the tissues of the body has resulted in a complex series of defences. Dealing with this challenge requires both tolerance to commensal bacteria and non-harmful substances and a strong response to infectious agents. The intestinal barrier, the mucous layer, and the commensal bacteria of the gut constitute the first line of defence against invasion. A healthy microbe contributes to colonisation resistance by inhibiting invading pathogens and suppressing overgrowth by potentially pathogenic bacteria [74]. The intestinal barrier includes the close-fitting tight junctions between the single layer of epithelial cells that line the colon, the protective mucus layer secreted by goblet cells and adhering to epithelial cells, as well as antimicrobial peptides (AMPs) secreted by specialised epithelial cells, and the production of IgA deposited on the luminal surface. There are a number of bacteria that are known to degrade mucin and so stimulate new mucin production to improve the intestinal barrier. These include *Akkermansia* (considered a mucin specialist) and some *Bacteroides*, *Bifidobacterium*, *Ruminococcus*, *Paraclostridium*, and *Prevotella* species [75]. *Akkermansia* can also increase the intestinal barrier integrity by activating the TLR2 pathway to produce IL-10 [76].

If the epithelium is breached, the innate and adaptive immune systems (macrophages, innate lymphoid cells, T cells, B cells) come into play [77]. The greatest proportion of the body’s immune system (up to 80%) is centred on the gut [78] as gut-associated lymphoid tissue. Somewhat paradoxically, the barriers against microbial invasion of the tissues and acquired immunity are developed and maintained by constant exposure to the gut microbiota. The correct functioning of the immune system depends on the establishment of a stable intestinal microbiota in the early years of life [79]. For example, germ-free mice do not develop an effective immune system, with a reduced capacity to produce IgA and IgG but increased IgE, resulting in an increased incidence of diverse diseases [80]. The extensive interface between the microbiome and the gut epithelium, with the intestinal barrier allowing some antigens to pass, means that large numbers of molecules can challenge the immune system to maintain immune surveillance [81]. The tight junction integrity of the intestinal barrier depends on the health of the microbiome and the production of SCFAs, which regulate the expression of proteins integral to tight junctions’ function [82].

The immune system has evolved to cope with the symbiotic association with the microbiome and distinguish foreign and invading microbes and toxins presented to it via the gut, facilitated by Toll-like receptors (TLRs) on epithelial and immune cells. These identify potential threats, such as Gram +ve cell wall subunits (peptidoglycan), Gram −ve cell membranes (LPS), and bacterial capsule polysaccharides. The result of the activation of TLRs is the instigation of inflammasomes and the release of AMPs.

## 5. The Microbiome–Gut–Brain Axis (MGBA)

**Figure 2:**
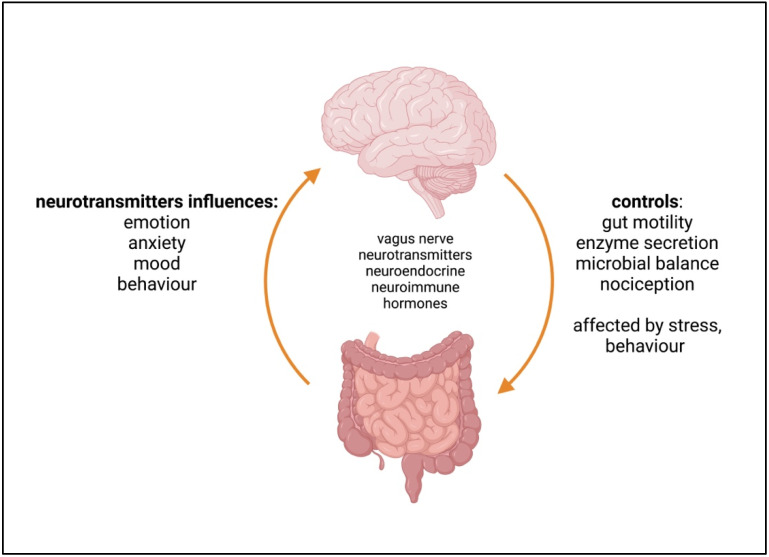
The bidirectional nature of the gut–brain axis. Created with BioRender.com.

The gut–brain axis refers to the intricate, bidirectional communication network between the central nervous system (CNS) and the gut (Figure 2). This communication network involves a complex interplay of neural, hormonal, and immune signalling mechanisms. Recent research has shown that the gut–brain axis plays a critical role in regulating a wide range of physiological processes and modulating a host of pathophysiologic processes. This evidence comes from germ-free animal studies, the use of faecal transplants or probiotics to restore function in animal models of neurodegeneration [83,84], and the association of a dysregulated microbiome with neurodegenerative and neurodevelopmental diseases [85].

In addition to acting locally, metabolites produced by the microbiome have systemic effects. A great deal of evidence has emerged over the last decade that gives weight to the bidirectional nature of the gut–brain axis: that is, the direct control that the brain has over the gut and the influence that the microbiome and gut have over the brain. In human studies, most evidence of a MGBA link comes from the association of dysbiosis with neurological disorders, including neurodegenerative, neurodevelopmental, and neuropsychiatric disorders. In animal studies, there is a great deal of direct evidence from germ-free animal models and from infection, antibiotic, and faecal transplant studies. However, the complete mechanisms that control this communication are not yet fully understood.

The brain communicates with the gut via interconnected neuronal, hormonal, and neuroendocrine pathways to control numerous gut functions. Emotions, for example (such as fear, anger, and sadness), can alter the gut microbiome, and traumatic brain injury significantly reduces the diversity of the gut microbiome [86]. The communication from the microbiome to the CNS can be direct and rapid via the vagal nerve, as well as by the slower, indirect communication via the neuroendocrine (hypothalamus–pituitary–adrenal—HPA) axis, immune signalling (such as cytokines), and metabolic products (such as SCFAs, hormones, and tryptophan metabolites). The MGBA can affect emotional responses and behaviour. For example, germ-free mice show reduced anxiety levels compared to mice with a normal microbiome [87], and mice infected with *Campylobacter* that cause diarrhoea exhibited symptoms of anxiety that were reversed by probiotics [88], although not when vagotomised [89]. While the gut microbiome influences behaviour, anxiety, and depressive symptoms, these same symptoms can also further disturb the microbiome, exacerbating symptoms [90]. Evidence is also accumulating that stress can influence gastrointestinal diseases in humans [83,91]. It has been shown that glucocorticoids generated from the HPA axis during stress reactions directly affect the ENS and gut inflammation [92]. A recent study has also shown that perceived stress, despite not necessarily affecting biochemical stress levels, can modulate the microbiome within one week in a number of different (individual) ways [93], which may reflect genetic or other differences between individuals.

The vagus nerve is the 10th cranial nerve and links the CNS to the viscera. It is a major element of the parasympathetic nervous system and the major pathway of communication between the brain and the gut, controlling gut function as well as non-gut functions (breathing, heartbeat, etc.). It connects to the gut via the ENS, a complex nerve network second in size only to the brain. The ENS consists of a fine network of nerves, including enteric neurons, enteric glia, peripheral ganglia, and intrinsic neurons, that interface directly with EECs and neuropod cells of the intestinal lining [94].

The vagus nerve, via the ENS, directly controls the functions of the gut, including gut motility, intestinal permeability, the secretion of enzymes and bile, mucous production, the adsorption of nutrients, and the feeling of satiation, many of which also have a direct effect on the microbiome. The vagus nerve also functions to regulate inflammation [95] and controls the functioning of the gut epithelium, with some evidence that it can alter gut permeability [96]. While the vagus nerve has no direct contact with the microbiome, it is able to sense the microbiota via the metabolites produced and via epithelial cells that are connected to the ENS. EECs detect signals from TLRs on their surface that recognise bacterial metabolites and produce 5-HT to interact with the vagus nerve 5-HT3 receptors. SCFAs and TLR4 also directly activate vagus nerve fibres [97]. It has been shown in mouse models that some probiotic bacteria (*Lactobacillus*) increase vagus activity; modify brain GABA, glutamate, and 5-HT; and reduce anxiety-related behaviour [88].

The other route of communication between the gut and the brain is via the neuroendocrine (HPA axis), neuroimmune, and metabolic routes. Metabolites that communicate with the brain are either directly produced by the microbiome or are stimulated or influenced by the microbiome. SCFAs produced by the microbiome stimulate EECs to produce and secrete hormones and other metabolites, including GLP-1, PYY, leptin, and ghrelin. These are released into the blood and lymphatic systems, which regulate energy production and can affect the CNS. GLP-1 and PYY promote feelings of satiety, which does not occur in germ-free mice [98]. Many of the disease conditions that involve the MGBA are associated with an imbalance of the HPA axis, such as IBS, insulin resistance [99], and depression [100].

A further avenue of communication in the MGBA might be a direct effect of the intestinal microbiome on neural mitochondria [101]. Metabolites produced by the microbiome or stimulated by the microbiome can have direct effects on the mitochondria in neurons and brain metabolism. SCFAs are able to cross the BBB and directly influence mitochondria in the brain to modulate energy production. GABA, 5-HT, and dopamine can also influence mitochondrial function.

### 5.1. Neurodevelopment

The microbiome, acquired at birth and developed through childhood and adolescence, is considered to have a major effect on development, particularly neurological development [90], via the MGBA, and dysregulation of the gut microbiome in early life can impact neurodevelopment [102]. In germ-free animal models, many aspects of neurodevelopment have been shown to be compromised, including neurogenesis and myelination [103] and dendritic spine development [104], as well as abnormal ENS development [105], which can be reversed by the post-natal transplant of a normal microbiome. The gut microbiome also appears to be essential for the successful development of the HPA axis [106], with a developing microbiome stimulating inflammatory markers such as TNF-α, IL-1β, and IL-6, which help mature the HPA axis [107]. While there have been far fewer studies in humans, microbial diversity in the gut has been implicated in the development of cognitive function and functional connectivity, especially in studies of ASD, where the maternal microbiome, microbiota establishment in utero, the mode of delivery, and antibiotic use (mother and child) have been implicated in microbiome development and impaired neurodevelopment [108]. A novel hypothesis for MGBA communication is that it acts via the microbiome’s potential influence on microRNA expression in the brain [109], which may affect neurodevelopmental gene expression.

### 5.2. Dysbiosis and Disease

**Figure 3:**
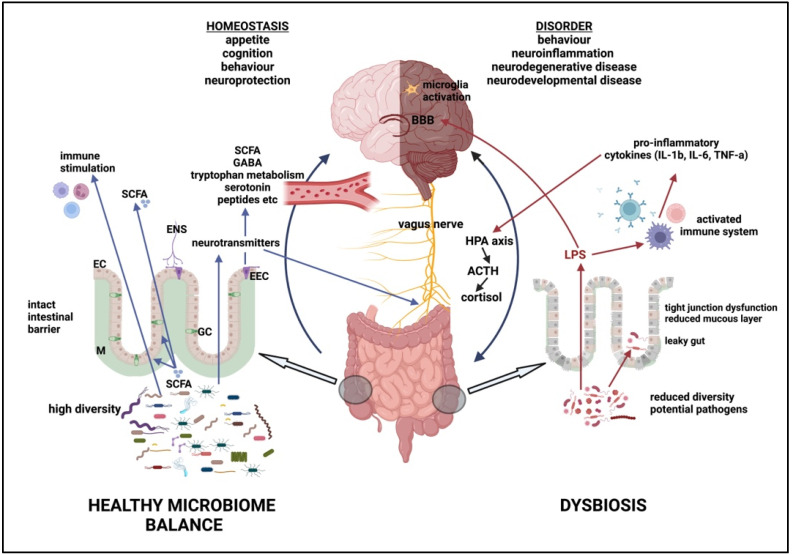
The microbiome–gut–brain axis in health and dysbiosis. ACTH—adrenocorticotropic hormone; BBB—blood–brain barrier; EC—epithelial cell; EEC—enteroendocrine cell; ENS—enteric nervous system; GABA—gamma-aminobutyric acid; GC—goblet cell; HPA—hypothalamic–pituitary–adrenal; IL—interleukin; LPS—lipopolysaccharides; M—mucous; SCFA—short-chain fatty acid; TNF-α—tumour necrosis factor alpha. Created with BioRender.com.

A balanced, healthy microbiome is essential to our body’s homeostasis. The disruption of the gut microbiome to an unbalanced state, generally referred to as dysbiosis, results in the loss of homeostasis and is associated with multiple diseases. Dysbiosis in the bacterial population of the microbiome cannot yet be accurately characterised in either taxonomic or functional terms, and even less is known about changes in archaea, fungi, viruses, and protists. There is, however, a general recognition that dysbiosis involves a change in the microbiome structure to less diversity, with the loss of what are thought of as beneficial bacteria (SCFA producers, mucin degraders) and their replacement with increased numbers of so-called “pro-inflammatory” bacteria, potential pathogens, and those that produce toxic bacterial metabolites (Figure 3).

Dysbiosis is associated with many diseases, including gastrointestinal, metabolic, and inflammatory diseases, such as obesity [110,111], IBD [112,113,114] and IBS [115,116,117], metabolic syndrome [111,118], T2DM [119,120,121,122], rheumatoid arthritis [123], hypertension [124], cardiovascular disease [125,126,127,128], systemic lupus erythematosus [129], non-alcohol liver disease [130], age-related loss of muscle mass and function (sarcopenia) [131,132], and alcohol-dependent inflammation [133]. There are also multiple brain conditions that are associated with dysbiosis via the MGBA (vagus or BBB), including depression and anxiety disorders [134,135,136], migraine [137], neuropsychiatric and mental health conditions [138], postoperative cognitive dysfunction (POCD) that can accompany anaesthesia and surgery, especially in the elderly [139], and potentially all of the neurodegenerative and neurodevelopmental diseases. Brain injuries are also associated with gut dysbiosis, but the injury is most probably causing the imbalance and intestinal inflammation [140], although a recent study has implicated gut dysbiosis as a risk factor for ischaemic stroke, with two genera (*Intestinimonas* and a genus in Lachnospiraceae; both SCFA-producing bacteria) identified as lowering the risk of developing ischaemic stroke [141]. The association of dysbiosis and disease does not necessarily reflect causation, and changes in the microbiome may instead be induced as a result of the disease. There is, however, increasing evidence as to the role of dysbiosis in the instigation and progression of many diseases, mostly from animal studies. Unravelling the complex interactions of the microbiome with the metabolic state of the body is a major undertaking and is currently at the beginning stage. Despite the great heterogeneity in the microbiomes between individuals, there appears to be some commonality in the dysbiosis of a number of metabolic and neurodegenerative conditions, including obesity, metabolic syndrome, IBD, IBS, T2DM, PD, and AD.

The loss of microbial diversity and the decrease in SCFA-producing bacteria (such as *Faecalibacterium*, *Roseburia*, and *Ruminococcus*) and mucin-degrading bacteria (such as *Akkermansia*) have a profound effect on gut health. Reduced levels of SCFAs have been associated with obesity, metabolic syndrome, T2DM, non-alcoholic fatty liver disease, neurodegenerative diseases (AD, PD), neurodevelopmental disease (ASD), neuropsychiatric disorders (anxiety, depression), and cancer development [62]. The reduction in SCFAs leads to increased gut permeability due to apoptosis of cells and the dysregulated expression of tight junction proteins, which in turn allows bacterial products, including LPS, microbial toxins, and even whole bacterial cells, to translocate across the intestinal barrier from the gut into the surrounding tissues (Figure 3). This so-called “leaky gut” leads to an increased inflammatory response [81] and to systemic inflammation, which is associated with multiple metabolic, neuroinflammatory, neuropsychiatric, neurodevelopmental, and neurodegenerative conditions. It is characterised by elevated levels of circulating pro-inflammatory cytokines, such as IL-1β, IL-6, TNF-α, and c-reactive protein (CRP) [142,143,144,145,146,147], which can further reduce gut integrity [148], thereby further increasing dysbiosis. In addition to the intestinal barrier, dysbiosis is also associated with the disruption of the BBB [149], allowing increased movement of bacterial metabolites into the brain and increasing neuroinflammation.

The increase in potential pathogens can include the Proteobacteria phylum, including *E. coli*/*Shigella* strains and *Salmonella*, as well as *Helicobacter pylori*, *Vibrio, Staphylococcus, Streptococcus, Enterococcus, Yersinia*, and some *Clostridium* and *Bacteroides* strains. Many of these bacteria produce toxins, including LPS, which produce a strong immune response, including septic shock [148]. *Bacteroides fragilis* produces an exotoxin that disrupts adherence junctions and further increases the leakiness of the intestinal barrier [150].

### 5.3. Dysbiosis Metabolites

LPS is a molecule that makes up a substantial proportion of the outer membrane of Gram −ve bacteria and is released after bacterial cell death. The passage of LPS through the intestinal lining activates the HPA axis [151] and provokes a very strong immune cytokine response, mediated by TLR4-CD14/TLR2 receptors, via NF-κB [98]. In a mouse model, dysbiosis is accompanied by increased plasma LPS, an intestinal inflammatory response, followed later by increased apoptosis, including in ENS neurons, mediated by TLR4 [152]. LPS also activates mast cells, which results in the release of tryptase, TNF-α, IL-1β, IL-4, IL-13, NF-κB, and pSTAT3, which activate an immune reaction and can cause further damage to the mucosa [153]. Corticotropin-releasing hormone released from mast cells increases the permeability of the BBB and so activates microglia and increases neuroinflammation [154].

Circulating cytokines, corticotropin-releasing hormone, and activated immune cells increase the permeability of the BBB, allowing the entry of bacterial metabolites and toxins into the brain. In addition, LPS also directly affects the BBB [98]. This, in turn, activates an immune response in the brain, activating microglia and recruiting macrophages and immune cells to the CNS, increasing neuroinflammation, and potentially hastening neurodegeneration [98]. In a healthy microbiome, the BBB is regulated via the vagus nerve and endocrine pathways, with SCFAs stabilising the BBB [98]. BBB disruption is a feature of AD, PD and multiple sclerosis (MS). Increased (undigested) protein supply to the colon stimulates protein-degrading bacteria and potential pathogens [155], affecting the intestinal barrier [156] and increasing amino acid metabolites such as indole, ammonia, amines, *N*-nitroso compounds, hydrogen sulphide, and methane, which have been associated with IBD and colon cancer [52]. Increased tryptophan levels can lead to the production of indole derivatives that are detrimental. For example, the overproduction of indole results in its conversion to indoxyl sulphate (in the liver), which is associated with chronic kidney disease (CKD) [62]. Histidine can be converted by bacteria (*Streptococcus* and *Eggerthella*) to histamine and to imidazole propionate (associated with T2DM) and ammonia [62]. Microbial breakdown products of phenylalanine and tyrosine have been implicated in neurological disease, PD, AD, kidney disease, and CVD [62]. l-Carnitine and choline in proteins are converted to trimethylamine (TMA) by some species of *Clostridium* and a number of genera in Enterobacteriaceae [157]. This is transformed into trimethylamine N oxide (TMAO) by enzymes in the liver, which has been directly linked to CVD and CKD [28,157].

Gut dysbiosis changes the production of 5-HT due to decreased SCFAs. 5-HT can affect CNS signalling and may be related to neuropsychiatric serotonin-related diseases (depression, anxiety, schizophrenia), and, importantly, in the developing brain, reduced 5-HT can influence normal neural development in ASD [158].

## 6. Causes of Dysbiosis

Modern human lifestyles have had a profound effect on the gut microbiome, as well as on the pattern of human diseases. Global disease incidence has transitioned from infectious diseases to autoimmune diseases, metabolic diseases, cardiovascular diseases, neuropsychological diseases (depression, anxiety), and neurodegenerative and neurodevelopmental diseases, all of which are associated with gut microbiome dysbiosis.

### 6.1. Diet

Dysbiosis tends to have a similar signature across many conditions, with dietary patterns being shown to be highly correlated with gut dysbiosis as well as inflammatory patterns in the gut across multiple dietary patterns, inflammation, and disease and control groups [33], with 393 associations between 123 bacteria and 61 food items, mostly in the same direction with respect to health versus disease.

Diet is the primary reason for dysbiosis worldwide. The influence of long-term diet on the gut microbiome is profound. A Western diet, with its emphasis on simple carbohydrates (sugars), high saturated fat, high protein, and highly processed foods with low dietary fibre content, has accelerated historical human microbiome changes [159]. The increasing acceptance of the Western diet over the last 60 years around the world is correlated with the rise in obesity and the increasing occurrence of the so-called lifestyle diseases (metabolic conditions, CVD), as well as gastrointestinal, metabolic, autoimmune, and neurological conditions. Clinical obesity rates have tripled in the last 40 years worldwide to 650 million, or 13% of the world’s population, with a further 1.3 billion (26%) being overweight in 2016. This included 41 million children under 5 and 340 million older children and adolescents [160]. Immigration to developed countries with a consequent change to a Western-style diet results in changes to the microbiome, including reduced diversity and a changed genus profile, and corresponds to increased obesity, which increases over the generations [161].

Diets with highly processed foods, animal products, and sugars promote a microbiome with increased inflammation [33]. This is especially true of ultra-processed foods, where nutrients are largely acellular and absorbed in the small intestine, with few nutrients available to the gut bacteria [162]. Ultra-processed foods make up the majority of the energy intake in the USA and are linked to dysregulated nutrient adsorption, an impaired glycaemic response, hypertension, reduced metabolic health, and increased mortality [29,162]. Highly processed foods are associated with some species of *Clostridium*, *Ruminococcus*, and *Blautia*, related to increased energy harvesting from energy-dense foods that pass through the small intestine and into the colon [33]. High-animal-protein diets, which are usually also high in saturated fat, can allow bacteria from the upper GIT and oral cavity to increase in number in the gut while increasing the number of opportunistic pathogens and pro-inflammatory conditions. There is also increasing evidence that artificial sweeteners [163] and emulsifiers [164] can directly affect the microbiome and promote pro-inflammatory conditions.

### 6.2. Ageing

Another major influence on the gut microbiome is age, which is also the major risk factor for neurodegenerative diseases. While the microbiome generally shows stability over time, there are accelerated changes in the composition of the microbiome in people over the age of about 65 years, and they are exacerbated with continued ageing. The microbiome composition of older humans [165] and mice [166], compared to healthy controls, is characterised by lower diversity, decreased SCFA producers, and increased numbers of potential pathogens, especially Gram −ve Enterobacteriaceae. That is, there is a typical dysbiosis shift from anti-inflammatory to pro-inflammatory bacteria. There is also a diminishment in the Firmicutes–Bacteroidetes ratio and a decrease in the genus *Bacteroides* [167]. This, together with an increasingly permeable BBB [168], age-related decreased immune response (macrophage activity, T-cell production), reduced gut motility, and reduced intestinal barrier integrity [169,170,171], sets the stage for the increased translocation of microbial products (such as LPS and TMA), an increase in pro-inflammatory cytokines, and a greater inflammatory response. This leads to chronic low-grade inflammation (or “inflammaging”), which is a hallmark of this age group, and which can become systemic and chronic [172]. Increased infections, increased medication use, poorer nutrition, sleep disturbance, and other stresses also contribute to this inflammaging, which can accelerate age-related diseases such as metabolic disease, T2DM, CVD, and neurodegenerative diseases [173].

### 6.3. Lifestyle

Myriad other environmental factors have been shown to have an effect on the microbiome [21], including cultural factors, gender [174], lifestyle, smoking, toxins, infections, and diseases. There is increasing evidence that a built environment, with the associated humidity and dust, has a negative effect on microbiome development, while increasing green spaces might reverse this to some extent [175]. Access to rural areas and soil has a positive effect on the gut microbiome of urban-dwelling individuals [176] and exposure to soil has an effect on the microbiome and innate immunity [177], most probably due to the long ancestral association of humans with soil and the more recent history of urbanisation.

Many environmental toxins, including pesticides, herbicides, hydrocarbons, metals, and other persistent chemicals, can find their way to the GIT, where they may not only be bio-transformed by the microbiota (such as methylation and metal immobilisation) but also negatively impact the microbiome [175].

Many socioeconomic factors can be related to microbiome dysbiosis, including alcohol dependency, smoking, poor diet, sedentary lifestyle, and reduced access to medical and dental care. Smoking has been shown to increase potential pathogens and decrease beneficial bacteria [178]. Alcohol use generally has an adverse effect on the microbiome [175], with alcohol dependency having the additional impact of reducing intestinal integrity [40]. Many commonly used drugs, both medicinal [179] and recreational [41,180], can influence the gut microbiome. While the impact of genetics is generally considered a minor influence in disease states such as neurodegenerative diseases, the epigenetics of the disease has a major impact on the disease and the microbiome [181].

### 6.4. Medications

The microbiome can be affected generally in an adverse way, by external toxins and ingested drugs. Many medications, including prescription, over-the-counter, and recreational drugs and dietary supplements, can influence the microbiome, with estimates of over 17 categories of drugs causing over 150 interactions [182] and almost 25% of drugs tested in vitro inhibiting one or more bacteria [183]. The most important categories of drugs that adversely affect the microbiome are antibiotics (designed to kill or inhibit microbes), proton pump inhibitors (PPIs), laxatives, lipid-lowering statins, and selective serotonin reuptake inhibitor antidepressants [184]. Antibiotics generally reduce bacterial diversity, increase microbial resistance to antibiotics, and potentially increase the number of potential pathogens such as *Clostridium difficile* [185,186,187].

PPIs are often prescribed for gastric conditions such as gastro-oesophageal reflux disease (GORD), gastrointestinal bleeding, *Helicobacter pylori* infection, or the prevention of gastric ulcers. They are some of the most prescribed drugs worldwide and are commonly over-prescribed or inappropriately prescribed [188]. PPIs significantly decrease microbial diversity and contribute to dysbiosis and, through a reduction of gastric acid production, favour the transfer of oral bacteria to the lower GIT and encourage the establishment of pro-inflammatory conditions, with increased Enterobacteriaceae (including potentially pathogenic *E. coli/Shigella* species), *Enterococcus*, *Campylobacter*, *Staphylococcus*, and other potential pathogens, as well as an increased risk of *C. difficile* infection [189,190].

Selective serotonin reuptake inhibitors (SSRIs) are commonly used to treat a broad range of psychiatric conditions, including depression, with estimates of up to 13% of the population of the USA being “prevalent users” [191]. The majority of the body’s serotonin is produced in the gut, where, amongst other effects, it influences gut motility. SSRIs may have side effects such as nausea, diarrhoea, constipation, and weight gain and are also known to have an adverse effect on the gut microbiome, acting somewhat like antibiotics to reduce microbial diversity [191], specifically reducing *Akkermansia* [192]. It should also be remembered that depression and other psychiatric disorders are also associated with gut dysbiosis [134]. Other antidepressants and antipsychotic medications can also affect the microbiome. Olanzapine induces weight gain, causes metabolic disturbances, and alters the gut microbiome [193].

Medications that are used to manage neurodegenerative and neurodevelopmental diseases can also influence the microbiome. Both levodopa and levodopa-carbidopa have been shown in a number of studies to adversely affect the microbiome [194,195], although, in a small longitudinal study, the initiation of levodopa medication was not accompanied by a significant change in alpha or beta diversity [196]. In addition, members of the gut microbiota, such as *Enterococcus*, are known to convert levodopa to dopamine, rendering it unable to cross the BBB [197]. Both Catechol-O-methyl transferase (COMT) inhibitors and anticholinergics have gastrointestinal side effects and have been shown to have a negative effect on the microbiome [195,198,199,200]. There have been fewer studies that address the interaction of Alzheimer’s disease medications with the microbiome [201], although, for anticholinesterase inhibitors and N-methyl-D-aspartate (NMDA) receptor antagonists that are in use, gastrointestinal side effects are common [202,203], which would suggest that dysbiosis might be involved. Of the medications approved for ASD, risperidone has been shown to cause dysbiosis [204], as has aripiprazole, an antipsychotic medication [205].

## 7. The Gut–Brain Axis and Neurodegenerative Diseases

Neurodegenerative diseases are characterised by progressive neuronal dysfunction and losses in different parts of the brain, leading to the loss of function and eventual death. Neurodegenerative diseases have a number of features in common. They are, for the most part, idiopathic; there is neuroinflammation, there is mitochondrial dysfunction and neuronal death, and there may be an accumulation of misfolded, aggregated proteins that spread in a prion-like manner. AD and PD are the most common neurodegenerative diseases, followed by a lower incidence of amyotrophic lateral sclerosis (ALS), Huntington’s disease (HD), multiple system atrophy (MSA), prion disease (Creutzfeldt–Jakob disease (CJD)), Batten disease, progressive supranuclear palsy, and cortico-basal degeneration. To this list we might potentially add long COVID, which has been shown to have neurodegenerative aspects. The most common neurodevelopmental diseases are ASD and attention-deficit hyperactivity (ADHD). In addition, chronic fatigue (CF), channelopathy diseases (epilepsy migraine with aura, etc.), and POCD, while not strictly neurodegenerative diseases, share some of the aspects of these diseases (such as neuroinflammation and gut dysbiosis). The fact that these are, theoretically at least, reversible may make them valuable as models of neurodegeneration amenable to treatment via the MGBA.

The single most important risk factor for neurodegenerative disease is age. As previously indicated, ageing results in the twin problems of microbiome deterioration and immune system decline, which leads to a leaky gut and accelerating inflammation (inflammaging) that the immune system can no longer effectively deal with. This inflammation, while systemic, is centred in the abdomen. This interaction between ageing, gut dysbiosis, and neurodegenerative diseases requires much further study of the triggers of disease, the links to other systemic inflammatory diseases, and the pre-disposition to develop different neurodegenerative diseases.

An altered gut microbiome composition has been reported for all neurodegenerative and neurodevelopmental diseases [103], and many patients with these diseases have gastrointestinal symptoms. The realisation over the last two decades of the interaction between the gut microbiome and the brain has resulted in a transformation in the way that neurological and neurodegenerative diseases are perceived and in the number of systematic reviews that have stressed the significance of these findings for diagnosis and potential therapeutic intervention [195,206,207,208,209,210]. Much of this evidence has come from cross-sectional studies that indicate differences between persons who suffer from the disorder and so-called healthy controls (the association of dysbiosis with disease), as well as animal models, where the manipulation of the microbiome is more practicable. While a causal link between dysbiosis and disease is difficult to demonstrate in humans, dysbiosis is often suspected to have a role in the initiation, manifestation, and progression of disease. In animal models, however, the use of germ-free mice and the manipulation of the microbiome with populations of bacteria or faeces from patients with neurodegenerative diseases have provided evidence of causation [32,211,212].

It is certain that there is a profound microbiome change in neurodegenerative and neurodevelopmental diseases [194,199,200,213,214,215,216,217,218,219,220,221,222,223,224,225,226,227,228], independent of the effects of sex, age, BMI, constipation, gastrointestinal discomfort, geography, and diet [195,206,207,208,209]. Dysbiosis is characterised by the loss of diversity, the loss or depletion of SCFA-producing bacteria and reduced SCFA levels [222] and increases in pro-inflammatory bacteria. In general, dysbiosis is characterised by the reduced abundance or loss of SCFA-producing *Faecalibacterium*, Christensenellaceae, *Collinsella*, *Roseburia*, some *Ruminococcus*, *Bifidobacterium*, *Bacteroides*, *Parabacteroides*, *Oscillospira*, some *Clostridium* and the mucin-degrading bacterium *Akkermansi.a* and by the overabundance of potential pathogens such as Enterobacteriaceae (*E. coli/Shigella*, *Klebsiella*, *Salmonella*), *Campylobacter*, *Enterococcus*, *Streptococcus*, *Staphylococcus*, *Fusobacterium*, *Veillonella*, some *Ruminococcus*, *Megasphaera*, and Deltaproteobacteria. Dysbiosis is accompanied by decreased gut integrity, which results in an increased inflammatory response [229,230,231] in neurodegenerative diseases. In addition to reducing intestinal integrity, a reduction in SCFAs can also reduce the effectiveness of the BBB, allowing microbial metabolites to enter the brain and stimulating neuroinflammation [232].

Despite the many reports of changes in the microbiome, there appears to be no consistent microbial signature for individual neurodegenerative or neurodevelopmental diseases. This may be due to individual variations in microbiomes and the individual nature of the disease, the progression of the disease, the stage of the disease and medication taken, and geographical and other differences between the populations studied, or it may be due to technological factors, such as the disparity of methods and analyses used to assess the microbiome in different studies.

### 7.1. Amyloid

The amyloid present in each neurodegenerative disease is distinct. There is an accumulation of amyloid-β and hyperphosphorylated tau in AD, α-synuclein in PD and MSA, huntingtin protein in HD, TPD-43 and FUS/TLS proteins in ALS, and prion protein (PrPc) in CJD. All of these proteins are misfolded aggregated proteins that spread in a prion-like manner [233].

Animal models of PD have shown that an altered microbiome can lead to a build-up of α-synuclein in the gut with transport to the brain, which truncating the vagus nerve can prevent [234,235]. Epidemiological studies of partial vagotomy in humans (as a previous gastric ulcer treatment) have shown that it is correlated with a reduction in the risk of developing PD [236]. The unaggregated forms of the pathological proteins amyloid-β, α-synuclein, and serum amyloid A are antibacterial peptides that participate in the immune system at the microbiome/gut/tissue interface [232]. Dysregulation of TLR signalling, stimulated by gut dysbiosis and a compromised intestinal barrier and subsequent local inflammation, may trigger α-synuclein aggregation [237]. Ageing may also increase the risk of the aggregation of amyloid protein and subsequent translocation to the brain via the vagus nerve [238]. Once in the brain, the aggregated protein spreads from neuron to neuron through the propagation of more aggregates.

Additionally, a number of bacteria also secrete amyloids. For example, Gram -ve enteric bacteria produce an amyloid protein called curli, which aids in adhesion and forms a substantial part (up to 90%) of the extracellular matrix of biofilms [11] in the gut. Other amyloids produced by the microbiome include FAP (*Pseudomonas*), chaplins (*Streptomyces*), and TasA fibres (*Bacillus*) [239]. Curli and other amyloids activate TLRs and other receptors to initiate an inflammasome. These amyloids have a similar structure to amyloids found in neurodegenerative diseases [11], such as amyloid-β, α-synuclein, and PrPc, which also initiate inflammasomes, possibly by the same mechanism. It also appears that bacterial amyloids may ”seed” pathological amyloid aggregation or trigger its formation through inflammation [11].

### 7.2. Parkinson’s Disease

PD is a heterogeneous, multisystem neurodegenerative disease and the second most common neurodegenerative disease in the world. While some causative genes have been identified [240] and there are combinations of gene loci that increase susceptibility [241], most PD is idiopathic and suspected to be related to a combination of genetic susceptibility and environmental factors [242], with toxins such as herbicides and pesticides being implicated [94,243]. The symptoms of PD accompany the build-up of aggregated α-synuclein and the death of dopaminergic neurons in the substantia nigra, with motor symptoms appearing when between 50% and 70% of dopaminergic neurons have been lost [244]. There is a very strong MGBA link in PD, and the majority of the evidence for the MGBA link in neurogenerative disease comes from the large number of studies conducted with PD. Gastrointestinal symptoms are common in PD, with up to 70% of PD patients having constipation or other GIT motility issues [245], with the prevalence increasing as the disease progresses [246]. These symptoms may pre-date motor symptoms by many years [247].

Aggregated α-synuclein can sometimes be detected in the gut prior to symptoms of PD [248], including in EECs and enteric neurons [94], and it has been suggested that at least some forms of PD can begin in the gut, from where it spreads to the brain via the vagus nerve. This gut-to-brain spread of PD was first proposed to occur due to invasion by an unknown pathogen via the vagus nerve [249,250]. The gut-to-brain hypothesis has been supported by a number of experiments that have shown that faeces transplanted from people with PD into transgenic mice that overexpress aggregated α-synuclein will worsen symptoms, whereas healthy control faeces will not [211]. Experiments have also shown that transport of α-synuclein can occur from the gastrointestinal immune system (where it acts as an antimicrobial peptide) to the ENS and hence to the vagus nerve and the CNS [234,235,251]. Rats that are fed bacteria that produce the bacterial amyloid curli deposit α-synuclein in neurons in their gut and brain [252]. In addition, surgical truncal vagotomy is protective against subsequent PD development in both animal models [235] and humans [236]. It is still not entirely clear whether changes in gut microbiota (due to a toxin or infection) begin the cascade of changes (inflammation) that leads to α-synuclein spreading from the gut to the brain or if the dysbiosis is a consequence of the disease process. While the occurrence of aggregated α-synuclein in the appendix suggested that an intact appendix might have a positive association with the risk of developing PD, recent evidence suggests that the appendix may act as a reservoir of microbiome diversity [253], with appendectomy increasing the PD risk and maintaining the appendix being protective against future PD [254].

A number of GIT diseases are associated with an increased risk of developing PD, such as IBD [255] and IBS [256]. There is an increased risk (44%) of the subsequent development of PD after the diagnosis of IBS, based on a Swedish population study of over 56,000 PD cases [257]. The use of certain antibiotics is also a risk factor for PD, again based on epidemiological studies [258,259]. Gut inflammation and a leaky gut are also hallmarks of PD, with an increase in pro-inflammatory cytokines compared to healthy controls (such as IL-1β, IL-8, IL-6, CRP, and TNF-α). In addition, calprotectin, which is an indicator of gut inflammation in IBD, is increased in PD [260].

Multiple studies have demonstrated an altered microbiome (dysbiosis) in PD [199,224,225,228,261,262,263,264]. The PD microbiome (as is typical in dysbiosis) shows reduced diversity (reduced α-diversity where analysed) compared to healthy controls. The genera that are most often found to be decreased or underrepresented in the microbiota of PD are the beneficial bacteria (SCFA-producing and anti-inflammatory), including *Faecalibacterium*, *Roseburia*, *Ruminococcus*, *Prevotella*, *Dorea*, *Bacteroides*, *Clostridium* cluster IV (leptum), and genera in the order *Lachnospirales*. SCFA levels in the gut have also been shown to be decreased [260], leading to increased gut permeability. Bacteria that are overrepresented in PD include *Akkermansia*, *Bifidobacterium*, and *Lactobacillus*, as well as the and opportunistic/potential pathogens [228] *Enterococcus* (endotoxin-producing) *Christensenella*, *Oscillospira*, *Corynebacterium*, *Alistipes*, some *Bacteroides*, *Megasphaera*, *Desulfovibrio*, *Streptococcus*, *Staphylococcus*, and the family Enterobacteriaceae (*E. coli/Shigella*, *Salmonella*, *Klebsiella*). A number of these bacteria produce LPS and other bacterial toxins, which increase pro-inflammatory cytokines when passing into the tissues. This in turn promotes the formation of aggregated α-synuclein and the disruption of the BBB. Increases in Enterobacteriaceae have been associated with motor symptom progression in PD [200]. It is somewhat paradoxical that *Akkermansia*, *Bifidobacterium*, and *Lactobacillus* are often found to be increased in PD and would, in other circumstances, be recognised as beneficial bacteria and are marketed as probiotics [262].

A recent large and in-depth (metagenomic) study of the gut microbiome in PD has confirmed that profound gut dysbiosis is associated with PD, with 30% of species affected, resulting in increased numbers of pathogens and increased microbial toxins and molecules that induce α-synuclein aggregation [262]. A total of 84 PD-associated species were identified, with 29 species depleted in PD and 55 species enriched. The enriched species, interestingly, did not include *Akkermansia* in this study.

A long-term Mediterranean diet has been shown to be associated with a lower risk of developing PD, with a Western diet having a higher risk [265], although changing to a healthier diet once PD is diagnosed may not change the disease trajectory [266]. Similarly, the consumption of coffee, tea, and polyunsaturated fatty acids has been associated with a reduced risk, while dairy products and saturated fatty acids have been associated with an increased risk [265].

### 7.3. Alzheimer’s Disease

AD is the most common neurodegenerative in the world and is characterised by the progressive accumulation of amyloid-β and tangles of hyperphosphorylated tau fibrils in the brain and gradual memory loss. Most AD (approximately 95%) is idiopathic, although there is a potential link with CKD [267], with CKD also having a strong MGBA link [268]. There have been a number of infective agents that have been postulated to be associated with AD, including hepatitis C virus, cytomegalovirus, herpes simplex virus 1, *Chlamydophila pneumoniae*, *Toxoplasma gondii*, and miRNAs [269]. In addition, amyloid-β aggregates in a prion-like manner, which might be instigated in AD from bacterial or fungal amyloids, as described for PD [269].

Similar to PD, there is a link between diet and AD, with Mediterranean [35,36] and ketogenic diets [270] being potentially protective and high-fat diets increasing the risk of developing AD later in life [271]. This is also confirmed by increased amyloid-β production, neuroinflammation, and AD symptomology in mouse models on a high-fat diet and an improvement in AD symptoms in those on a ketogenic diet [272]. A retrospective study of over 300,000 people in Korea found that the use of antibiotics increased the risk of developing AD cumulatively [273].

There have been far fewer microbiome studies in AD than in PD thus far, and the numbers of microbiome samples are small. However, the AD microbiome is also characterised by decreased microbial diversity, increased pro-inflammatory bacteria, and decreased anti-inflammatory bacteria, but as yet, there is little consensus between studies [272]. Decreases in SCFAs have been associated with AD in animal models [173]. One study found that AD had decreased Firmicutes and *Bifidobacterium* with increased Proteobacteria [274] compared to controls. A second study confirmed this [275], while yet a third found decreased Bacteroidetes and increased Actinobacteria [276]. There is also some evidence that MCI and early-stage AD already show signs of pro-inflammatory bacteria increases, such as *Escherichia/Shigella*, in addition to increased inflammation [68]. Animal models of AD have also indicated that the microbiome is altered in AD and is involved in amyloid-β formation, neuroinflammation, and possibly BBB impairement via microbial metabolites [231,277].

### 7.4. Multiple Sclerosis

There have been a number of studies that have addressed microbiome changes in MS with relatively large numbers of patients. There appears to be a strong MGBA link in MS, with almost a third of patients with MS having a history of gastrointestinal symptoms prior to diagnosis, especially constipation and diarrhoea [278], and over 80% of patients having concurrent gastrointestinal symptoms [279].

Although there is variation between studies, generally dysbiosis showed predictable changes in the microbiome, with decreases in *Butyricimonas*, *Prevotella*, *Clostridia* clusters XIVa and IV, *Faecalibacterium*, *Eubacterium*, *Bacteroides*, *Parabacteroides*, *Intestinibacter*, *Roseburia*, *Butyricicoccus*, and *Gemminger* and increases in *Methanobrevibacter*, *Streptococcus* and *Akkermansia*, *Eggerthella*, *Pseudomonas*, *Mycoplana*, *Haemophilus*, *Blautia*, *Dorea*, *Clostridium bolteae*, *Ruthenibacterium*, *Holdemania*, *Anaeromonas*, *Lactobacillus*, *Olsenella*, *Sporobacter*, *Escherichia*/*Shigella*, and some *Clostridium* species [280,281,282,283,284,285,286,287]. As with PD, many studies found an increase in *Akkermansia* and some *Lactobacillus* species.

### 7.5. Amyotrophic Lateral Sclerosis

There have been few microbiome studies with ALS, although an epidemiological study showed a correlation between antibiotic use and ALS [288]. In one small study (six patients), ALS patients showed a worsened Firmicutes-Bacteroidetes ratio, a reduction in beneficial bacteria (*Anaerostipes, Oscillibacter,* Lachnospiraceae), and an increase in *Dorea* [289]. A second small study (eight patients) found an increase in *Methanobrevibacter* and decreases in *Faecalibacterium* and *Bacteroides* in ALS patients. One relatively large study (50 patients, 50 healthy controls) found reduced microbial diversity in ALS [290], while a second large study found no significant differences between patients and healthy controls, nor any correlation between the microbiome and disease progression [291]. Animal studies have indicated that ALS mouse models have reduced levels of butyrate and butyrate-producing bacteria, as well as reduced intestinal and BBB integrity, and that there may be a microbiome link with nicotinamide and disease progression [292].

### 7.6. Multisystem Atrophy

MSA is a rare and rapidly progressive neurodegenerative condition that can have different effects depending on the area of the brain that is affected, such as low blood pressure, bladder problems, or balance and movement issues (Parkinsonism), and can be initially misdiagnosed as PD. Like PD, MSA is an α-synucleinopathy, but with the aggregate α-synuclein deposited in the glia [293]. Constipation is also common in MSA [294].

Few studies have assessed the microbiome in MSA. In one study of 6 MSA patients and 11 healthy controls, pro-inflammatory bacteria increased, anti-inflammatory bacteria decreased, and intestinal integrity was compromised in MSA patients, with increased LPS-induced TLRs [295]. In a second study with 15 patients and 15 healthy controls, the microbiome was significantly different, with increased pro-inflammatory bacteria (*Alistipes*, *Streptococcus*, *Staphylococcus*) and decreased anti-inflammatory bacteria (*Bacteroides*, *Bifidobacterium*, *Blautia*, etc.) in the MSA patients [296]. In a review of neurodegenerative disease, there was a minor overlap in the microbiomes of the two α-synucleinopathies, PD and MSA [297].

### 7.7. Huntington’s Disease

The frequency of HD in European ancestry communities ranges between 3 and 7 instances per 100,000 and appears to be rather consistent over generations and has a strong genetic component [31]. While there has been little research on the gut microbiome in HD patients, there is some evidence of gut dysbiosis in HD, including a transgenic mouse model of HD [298]. In the only human study published to date, significant differences were found in the microbiome in HD compared to healthy controls, with reduced species diversity and differences between the microbiomes at the phylum (Firmicutes) and family (Lachnospiraceae) levels, with increased *Akkermansia* [144].

### 7.8. Creutzfeldt–Jakob Disease

While the MGBA is acknowledged as important in neurodegenerative disease, research into the microbiome in CJD is lacking. A single human study with 10 patients and 10 healthy controls found no significant difference in diversity between the groups, but there was a significant decrease in *Roseburia*, *Holdemanella*, and Lachnospiraceae, as well as in SCFA levels, and an increase in *Bifidobacterium* and the Proteobacteria and Fusobacteria phyla, both associated with disease [299].

### 7.9. Autism Spectrum Disorder

Autism spectrum disorder is a common neurodevelopmental disorder affecting between 1% and 2% of children, characterised by impaired social interaction and communication, as well as repetitive patterns of behaviour. Up to 70% of children with ASD have a comorbidity of gastrointestinal impairment, such as pain, diarrhoea, constipation, and vomiting [300,301]. Multiple studies have demonstrated changes in the gut microbiota compared to a healthy microbiome [103,302] and decreases in SCFAs have been associated with ASD in humans [173]. It is unclear whether a changed microbiome precedes the acquisition of symptoms of ASD. This is further complicated by the limited food choices of children with ASD, with a preference for processed foods while rejecting fruit and vegetables [303], as well as a resistance to dietary change. However, transplantation of a microbiome from ASD individuals into germ-free mice can induce autistic behaviours [304]. There is also evidence in animal models that a mother’s diet during gestation can affect the neural development of the foetus during this crucial period. Epidemiological studies suggest that children born by caesarean can have an increased risk of developing ASD [305], as can the use or overuse of antibiotics during the crucial neurodevelopmental period [108,301]. In a study assessing the microbiome structure of mother–child pairs, there was a correlation between the microbiomes of mothers and children with ASD [306].

Although many microbial signatures of ASD have been suggested [306], these do not appear to be consistent over multiple studies [302], possibly due to the high variability of ASD microbiomes, compounded by differences in medication and diet, as well as age and sex, all of which are known to affect the microbiome structure. Children with ASD produce lower levels of SCFAs [305] and can have increased gut permeability [307]. Overall, the ASD microbiome is less diverse than its healthy counterpart and can be depleted in carbohydrate-fermenting bacteria such as *Prevotella* and *Caprococcus* and often has an increase in Enterobacteriaceae, *Streptococcus*, and *Clostridium* and other potential pathogens [307], as well as *Akkermansia* [308]. The examination of public gene databases identified the genus *Desulfovibrio* as significantly associated with ASD (and ADHD) [154]. A potential signature of ASD is *Clostridium bolteae,* which is consistently found in higher concentrations in ASD microbiomes in studies and in datasets [302]. There is also a suggestion that ASD can be caused by a *Clostridium* neurotoxin that is transmitted via the vagus nerve and blocks neurotransmitters in the brain [309,310,311]. Children with ASD are also more likely to have a higher prevalence of small intestinal bacterial overgrowth (SIBO) than children without ASD [312].

### 7.10. Attention-Deficit Hyperactivity Disorder

ADHD is the most common neurodevelopmental disorder with 5% or more children worldwide, characterised by inattention, hyperactivity, and impulsive behaviour [313]. While few studies have investigated the MGBA and ADHD and few generalisations can be made [314], one small study has shown an increase in *Bifidobacterium* [315], while another found a decrease in the SCFA-producing *Faecalibacterium* [316] compared to controls. A third study found an increase in Ruminococcaceae*_UGC_004*, which was associated with symptoms of inattention [317]. A search of an ADHD database (19,000 patients) identified *Desulfovibrio* as significantly associated with ADHD [154].

### 7.11. Long COVID (Chronic COVID)

Diseases that have features in common with neurodegenerative diseases, such as neuroinflammation and an MGBA link, and are potentially reversible might be possible surrogates for the development of treatments for neurodegenerative diseases.

Post-viral symptoms are common after SARS-CoV-2 infection, with over 70% of acute infections (hospitalised or not) progressing to long COVID after some months [318]. It is now being recognised that long COVID can have a number of neurological symptoms, such as chronic headache, fatigue, anxiety, sleep disturbances, and olfactory loss [319], with one of the most common symptoms being cognitive dysfunction [318,320,321], commonly referred to as “brain fog”. There is some thought that cognitive dysfunction in long COVID may increase the risk of developing neurodegenerative diseases, including AD and PD [319,322]. As a potentially reversible cognitive dysfunction, long-COVID brain fog may stand as a model for the treatment of intractable neurodegenerative diseases.

A number of small studies have shown that there is a change in the microbiome with long COVID [323], with increases in potential pathogens, including *Streptococcus* and *Rothia*, and some *Clostridium*, *Actinomyces*, and *Bacteroides* species and increased LPS-producing bacteria, as well as reductions in *Faecalibacterium*, *Eubacterium*, and some *Bifidobacterium* species and other SCFA-producing bacteria [318,324,325].

### 7.12. Chronic Fatigue/Myalgic Encephalomyelitis

Chronic fatigue (CF) is an underdiagnosed syndrome with multisystem symptoms, including debilitating fatigue, weakness, sore throat, tender lymph nodes, and poor sleep, as well as neurological symptoms. The constellation of neurocognitive symptoms in CF is similar to that seen in mild traumatic brain injury [320]. Many people who suffer from CF also have GIT symptoms, often IBD [326,327], and its onset seems to be triggered, in some cases, by an infection or toxin [328]. A number of studies have demonstrated an altered microbiome in CF patients [326,329,330], although, as with other diseases, there is no recognised microbial signature. Generally, there is a decrease in diversity, an increase in pro-inflammatory Enterobacteriaceae (LPS-producing), *Streptococcus*, *Coprobacillus,* Eggerthella, and *Blautia,* a decrease in anti-inflammatory Firmicutes species such as *Faecalibacterium* and *Roseburia*, and increased intestinal permeability [327].

### 7.13. Epilepsy and Other Channelopathies

Novel areas that have an MGBA connection is epilepsy and related channelopathies, including migraine with aura and pain channelopathies [331]. These diseases have in common membrane dysfunction that results in a hypersensitive neural response to inflammatory stimuli and pain. There is increasing evidence that the MGBA modulates the communication of the neuroimmune, neuroendocrine, and direct neural communication pathways via the vagus nerve in epilepsy [332]. In an animal model, a healthy microbiome was shown to be a fundamental requirement for the normal development of inflammatory pain sensation, which is needed for an organism’s survival [333]. The gut microbiome has increasingly been shown to regulate neuropathic pain in pre-clinical studies [334,335].

### 7.14. Post-Operative Cognitive Dysfunction

POCD is the loss of cognitive function after surgery and/or anaesthetics. While usually temporary, it may lead to an increase in the incidence of MCI and AD [336], although results from studies are somewhat conflicting. With an ageing population and an increase in longer surgery procedures, the incidence of POCD is increasing, currently estimated at between 25% and 42% 7 days after surgery and 10% at 3 months in older patients (>60) [337]. As with AD, POCD involves neuroinflammation due to BBB disruption and anaesthetic stress [337]. A review of nine publications addressing microbiome changes with POCD incidence in animal models indicated significant changes in the microbiome in POCD mice after anaesthetic and surgery [338], with reductions in *Firmicutes* and increases in Proteobacteria [339]. In addition, there is evidence of the link between POCD and AD, with an increase in amyloid-β and hyperphosphorylated tau in animal models and patients with POCD [340]. One of the few human studies showed that elderly patients with POCD had reduced microbiome diversity with increased *Anaerofilum* (a genus common in depression) and decreased *Fusicatenibacter*, *Coprococcus*, and *Dorea*, among others [341]. While the change in microbiome structure in POCD is due to the stress of the procedure, combined with perioperative antibiotics and opioids and restricted nutrition, it is postulated that this dysbiosis, acting on the BBB, might contribute to POCD [340].

## 8. Microbiome Alteration as a Therapeutic Target for Neurodegeneration and Neurodevelopmental Diseases

The association of the gut microbiome and PD suggests the twin possibilities that the microbiome or, more specifically, a change in the microbiome, might eventually become a diagnostic tool for the earlier detection of neurodegenerative disease and that the gut might be an appropriate therapeutic target for neurodegenerative diseases [342]. Restoring the microbiome in animal models of neurodegenerative diseases by faecal microbiome transplants (FMTs) [84,343,344], diet [345], probiotics [85,346], or photobiomodulation [347] suggests microbiome-based therapy as an avenue of treatment for a range of diseases, especially those that are intractable and have limited treatment options. Current treatments for neurodegenerative and neurodevelopmental diseases (such as Levodopa for PD) can only offer patients brief respite from symptoms and can have adverse side effects. In order to capitalise on the link between the microbiome and the brain as a therapeutic option, there are numerous trials of therapies currently being conducted, with the aim of restoring a balanced microbiome and potentially altering the course of these brain conditions.

### 8.1. Diet and Prebiotics

Dietary interventions, such as changed dietary habits and/or supplements, have the potential to alter the microbiome in a positive way. Healthy diets such as the Mediterranean diet, a traditional Japanese diet, Dietary Approaches to Stop Hypertension (DASH), the Mediterranean-DASH diet intervention for neurodegenerative delay (MIND) diet, vegetarian diets, and so on are known to produce a more balanced, anti-inflammatory microbiome over time [27] and can reduce the risk of later development of neurodegenerative diseases such as PD [266] and AD [348,349,350,351]. While a diet change can help with gastrointestinal diseases, such as IBS [352] and IBD [353], and inflammatory diseases, such as rheumatoid arthritis [354], as well as the gastrointestinal symptoms of neurodegenerative diseases [266], the ability of a dietary change to alter the trajectory of neurodegenerative and neurodevelopmental diseases is less clear and has so far shown mixed results [266,355,356,357]. The MIND diet has been shown to improve cognition and slow cognitive decline in older adults, even when cohorts include Alzheimer’s disease patients [358], and a ketogenic diet improved the quality of life and daily function in AD patients [359].

A prebiotic is a plant fibre supplement that will pass through the small intestine undigested to enter the colon and act as a substrate for beneficial bacteria of the microbiome to ferment. Non-fermentable fibres such as cellulose are not prebiotics. Fermentable fibres such as inulin, fructans, galacto-oligosaccharides, and methylcellulose are defined as prebiotics since they are utilised by the microbiome and produce a benefit. The use of soluble fibres as prebiotics is a key element in changing the microbiome structure and reversing dysbiosis [360], although there is debate about the value of supplementation versus inclusion in the diet [361]. In animal models, the effects of prebiotics can be profound. Abnormal social behaviour in a mouse model of ASD, induced by a high-fat diet, can be reversed with prebiotics [362]. Supplementation with inulin in mice can discourage pro-inflammatory Proteobacteria and encourage *Bifidobacterium* and *Lactobacillus* (SCFA-producing bacteria) [363] and *Akkermansia* [364]. Psyllium fibre is digested much more slowly than other soluble fibres and has less of an effect on the microbiome [365]. Supplementation with psyllium can reverse inflammation, reduce the severity of colitis, improve the microbiome in mice [366] and reverse symptoms in a diabetic rat model [367].

In humans, the effects of prebiotics are less easy to establish and have often been inconclusive, possibly due to heterogeneity of the gut microbiome and differences in study design. There is a large variation in the response to prebiotics in human studies due to the myriad factors involved, including the diet, dose, intervention time, and starting microbiome. For example, in a systematic review of clinical trials using inulin, most studies found increases in *Bifidobacterium, Lactobacillus*, and *Anaerostipes* and decreases in *Bacteroides*, but this was not consistent with the increases in SCFAs in animal studies [368]. While prebiotics can encourage certain groups of bacteria to increase in number, they do not increase microbiome diversity [361].

### 8.2. Probiotics

Probiotics are bacteria that have beneficial effects on the health of the person. Most probiotic studies use *Bifidobacterium*, *Lactobacillus*, and yeasts as probiotic cultures. Recently, *Akkermansia* joined this list. It is important to consider that these three genera are often increased in the microbiome in many neurodegenerative diseases, such as PD, HD, ASD, MS, and MSA. Doses in studies are usually between 10^9^ and 10^10^ colony-forming units (CFU) for 4 weeks.

There have been many trials of probiotics for multiple gastrointestinal, metabolic, and neurodegenerative/neurodevelopmental diseases, mostly in animal models. Probiotic studies have shown substantial improvements in multiple animal models of neurodegenerative [231,369,370,371,372,373] and neurodevelopmental diseases [374,375], PD [371,376], and cognitive decline [377] and reductions in symptoms in MS [378], ALS [379], and ASD [380]. Novel probiotics have also shown promise in animal models. *Akkermansia* has been shown to treat such things as obesity [381] and depression [382]. *Clostridium butyricum* (an SCFA-producing bacterium) can improve gut permeability, reduce inflammation, repair the BBB, and reverse neurological symptoms in a mouse model [98]. Treatment with probiotics in animal models of POCD was seen to have a positive effect on cognition [337].

Results from human trials are much more equivocal. A randomised placebo-controlled trial (RCT) of 30 AD patients showed significant improvements in Mini-Mental State Examination (MMSE) scores for the treated group over the placebo group, with some improvements in some metabolic markers, including CRP and insulin sensitivity [383]. This was also the case with 26 AD patients compared to controls [384]. In contrast, an RCT of 25 AD patients found no difference in the trail-making test. A larger RCT (50 + 50) [385] found improved cognition in a number of cognitive tests and improved BDNF. A single RCT for PD [386] found a significant improvement in the Unified Parkinson’s Disease Rating Scale (UPDRS), as well as a reduction in CRP and an improvement in insulin sensitivity.

A recent review of the use of probiotics for ASD and ADHD found only one trial that had a positive effect among the seven trials that were included in the review [387]. In this trial, expectant mothers who received a *Lactobacillus* probiotic had a reduced risk of having a child with ASD or ADHD diagnosed at age 13, which was also correlated with reduced *Bifidobacterium* at age 6 months [388]. There is some pre-clinical and anecdotal evidence that probiotics are able to improve gastrointestinal symptoms in ASD, although clinical trials do not support this as yet [389].

### 8.3. Faecal Microbiome Transplant

Given the established microbiome association with many diseases, replacing a microbiome showing dysbiosis with a healthy mix of microbiota would appear to be a reasonable pathway to therapy. Currently, FMT does not have a standardised protocol but is accepted as the preferred treatment for recurrent or refractory *C. difficile* infection (CDI) and has been used since the early 1980s. The treatment is highly effective [390] and is the recommended treatment for this disease [391]. Recently, the United States Food and Drug Administration (FDA) approved FMT for use to treat and prevent the recurrence of CDI that causes colitis and diarrhoea, and the Australian Therapeutic Goods Administration (TGA) approved FMT more generally. FMT is being used in many open-label studies for other conditions.

FMT shows promise for GIT diseases, including IBD [392], IBS [393,394], Crohn’s disease and ulcerative colitis [395,396], as well as metabolic diseases such as metabolic syndrome [397,398,399,400] and T2D [401]. Recently, Wang et al. published a review of FMT, with 85 conditions that the therapy has been used to treat between 2011 and 2021 [402]. For neurodegenerative diseases, much of the early research has been conducted with animal models, either transplanting from a diseased animal model or human patient into a healthy or germ-free animal or transplanting from a healthy animal into a disease model of AD, PD, MS, ALS, and ASD [403]. Not only has this reinforced the strong microbiome–gut–brain-axis link in neurogenerative and neurodevelopmental diseases, but it has also demonstrated the efficacy of FMT in animal models. In humans, the effectiveness of FMT is less clear in conditions other than CDI.

Hazan [404] reported a single case study of an 82-year-old male with AD who presented for FMT to treat CDI with his 85-year-old wife as the donor. His MMSE score subsequently improved from 20 to 29. A second case study of a 90-year-old woman, also with CDI, who received FMT from a young donor also showed improvements in cognition [405]. In a single case study, a PD patient with severe intractable constipation was treated with FMT, resulting in the immediate resolution of constipation and a reduction in leg tremor after one week [406]. Six patients with PD were treated with FMT and showed some improvement in motor and non-motor symptoms as well as constipation up to 24 weeks after treatment [407]. A case series of 15 patients receiving FMT showed significant improvements in sleep quality and motor and non-motor symptoms when given colonic FMT, but not nasogastric FMT [344]. These improvements persisted in two participants for more than 2 years. A case series of 11 PD patients with constipation showed a reduction in constipation and significant improvements in measures of PD symptoms over a 12-week period [408].

Three MS patients were treated with FMT for constipation, all of whom improved in their symptoms and one of whom regained the ability to walk and went into remission for 15 years [409]. A single-subject case study of a patient who presented for CDI stabilised her disability score for 10 years after FMT [410]. A second single-subject case study of an MS patient with bloating showed improved walking and gait [411]. In 2016, a case series of seven ASD patients showed some improvement in symptoms of ASD after FMT [412]. In an open-label study with 18 children with ASD, vancomycin treatment for 2 weeks, a bowel cleanse, and 8 weeks of FMT resulted in improvements in GIT symptoms, ASD behavioural symptoms, and microbiome diversity for the 8 weeks of the trial [301]. Metabolite profiles of ASD patients also converged with control participants with this treatment [413]. Interestingly, the improvement in symptoms persisted and, in some cases, further improved at a 2-year follow-up, including the improvement in the microbiome [414].

Risks of FMT include some short term reactions to the therapy, such as bloating, diarrhoea, constipation, nausea, and fever [415]. There is also the potential for longer-term adverse events, including the risk of transmission of pathogenic and infectious agents (bacteria, viruses, fungi), multi-drug-resistant bacteria, infectious material (such as prions), and non-infectious substances (such as antibiotics) between the donor and recipient. There have been cases of disease transfer where the infective agent or cause is unknown, such as the transfer of obesity, IBS, and rheumatoid arthritis. A systematic review of FMT publications identified a rate of serious adverse events of between 2% and 6% [416]. There is more research needed for this therapy to determine the best preparation, optimal dose, frequency of transplantation, and long-term effects on health. The key to the success of FMT is the quality of the donor material. While there is no common standard for donor screening as yet, it is common to screen for viruses, pathogens, and donors with active disease or recovering from disease.

### 8.4. Medications and Supplements

Due to the importance of SCFAs, particularly butyrate, in gut health, the administration of therapeutic butyrate would seem to be an attractive option. While animal studies have been promising [417], with butyrate preventing weight gain and improving insulin sensitivity in high-fat-fed mice, clinical trials are less clear-cut. There is some evidence of a benefit in T2DM by oral or rectal administration [417], but not so in IBD [418].

Melatonin has been shown in mice to modulate the gut microbiome, improve the Firmicutes–Bacteroidetes ratio, and increase the abundance of *Akkermansia*, as well as decrease obesity in a high-fat-diet mouse model [419]. Also in mice, melatonin was found to reverse the dysbiosis caused by stress, with increased *Akkermansia* and *Lactobacillus,* and to decrease some *Bacteroides* species and Erysipelotrichaceae [420].

Disease-modifying antirheumatic drugs (DMARDs) are used to treat rheumatoid arthritis and act to suppress the immune response and attenuate inflammation by blocking the protein synthesis of cytokines. They can also modulate the gut microbiome, with a number of studies reporting that the use of this class of drug restores the microbiome to a non-dysbiosis or a healthy state [123].

While statins have generally been reported to have an adverse effect on the microbiome [421], a recent study reported that, in an obese cohort, the prevalence of dysbiosis (17.73%) was reduced (5.88%) by the use of statins [422]. Similarly, statins were found to restore the microbiome by increasing beneficial bacteria in patients with acute coronary syndrome [423] and to reduce serum levels of TMAO, potentially via microbiome alterations [424].

Metformin and other T2DM medications have been shown to exert at least part of their anti-diabetic effect of normalising blood glucose (insulin resistance and glucose homeostasis) via the microbiome. Metformin has been shown to increase SCFA abundance and *Akkermansia* levels in the colon of both mice and humans [183,425]. The effect goes beyond this one genus, with widespread changes in the microbiome [184,426], not all of which are positive. For example, *E. coli* was found to be increased and *Intestinibacter* was found to be decreased among patients on metformin who experienced gastrointestinal side effects such as bloating, nausea, and diarrhoea [184]. Other anti-diabetic drugs are associated with mixed microbiome changes in different studies [427], including reduced Proteobacteria and *Propionibacterium*, but also reduced *Butyricicoccus*, *Bacteroides*, and *Blautia* and increased SCFA-producing bacteria (*Faecalibacterium*, *Roseburia*, etc.), *Lactobacillus* and *Bifidobacterium*.

In summary, the judicious use of medications designed to improve the microbiome might have some promise for the treatment of neurological diseases. Whether these medications specifically target the microbiome as a primary effect or whether the microbiome is improved as a secondary effect with the improvement in inflammation brought about by these medications would need to be teased out with further research.

### 8.5. Traditional Chinese Medicine and Herbal Therapy

A recent review of the effect of traditional Chinese medicine (TCM) and herbal medicines concluded that many herbs and combinations of herbs are capable of having positive effects on the microbiome, increasing microbiota diversity, improving the intestinal barrier, and reducing inflammation [428]. Most studies have been conducted on animal models of IBS, IBD, and T2DM, but they might have implications for neurodegenerative and neurodevelopmental diseases. Specific TCM treatments have been shown to reduce potential pathogens, increase SCFA-producing bacteria, and improve gastrointestinal symptoms and the intestinal barrier. In mouse models, these include a combination of curcumin, aloe vera, slippery elm, guar gum, pectin, peppermint oil, and glutamine [429]; a gegen qinlian decoction [430]; a diallyl disulphide garlic extract [431]; a Pi-Dan-Jian-Qing decoction [432]; Qingchangligan [433]; a Buyang Huanwu decoction [434]; Schisantherin A [435]; Chimonanthus nitens Oliv. leaf [436]; Lycium barbarum glycopeptide [437]; a Banxia Xiexin decoction [438]; and a Qing-Fei-Pai-Du decoction [439].

In human trials, konjaku flour was shown to improve the microbiome and serum cholesterol in obese participants [440], and a Tanhuo decoction improved outcomes over Western medicine for acute ischaemic stroke patients as well as improved the microbiome [441].

Curcumin is a plant polyphenol that, despite having low systemic bioavailability, nonetheless has many physiological effects, suggesting a direct effect on the microbiome, including transformation into more bioactive forms. There is some evidence that curcumin can promote beneficial bacteria and improve biodiversity, improve the intestinal barrier, and reduce pro-inflammatory cytokines [442]. Curcumin has also been shown to be active in a 1-methyl-4-phenyl-1,2,3,6-tetrahydropyridine (MPTP) model of PD in mice, reducing α-synuclein aggregation and improving motor deficits [443].

A recent review [444] of the effect of ginseng in animal models and humans concluded that as well as improving the gut microbiome, ginseng can have beneficial effects on such diseases as obesity, diabetes, non-alcoholic and alcoholic fatty liver disease, colitis, diarrhoea, and various cancers. Interestingly, ginseng was shown to reduce α-synuclein aggregation and neuroinflammation in an MPTP mouse model of PD [445] and to reduce amyloid-β [446] and hyperphosphorylated tau [447] in animal models of AD.

Berberine has been shown to have an effect on a number of metabolic disorders as well as alter the microbiome in what could be considered a positive direction in both animal models and in a limited number of human trials. Improvements in the microbiome include increases in the numbers of SCFA-producing bacteria and mucin-degrading bacteria (*Akkermansia*) [448,449] and decreases in LPS bacteria (Proteobacteria and *Desulfovibrio*) [425].

The combination of moxibustion and acupuncture was shown to improve the microbiome in an RCT for Crohn’s disease, with increases in the numbers of SCFA-producing bacteria (*Faecalibacterium, Roseburia*) [450], possibly due to an improvement in the intestinal barrier, as demonstrated in a rat model [451]. A number of studies have shown positive effects on the microbiome in animal models of colitis and IBS treated with moxibustion and acupuncture [452,453,454,455,456]. Preliminary evidence has also suggested that acupuncture can improve the gut microbiome in animal models of PD [457,458], and an 8-week course of electroacupuncture to the scalp and abdomen significantly decreased a number of pro-inflammatory bacteria in an RCT (15 + 15), as well as improved some symptoms of Parkinson’s disease [459]. Similarly, acupuncture was shown to alter the microbiome in a mouse model of AD [460].

The effect of vitamins on the microbiome has recently been reviewed [461]. Some vitamins (with the most evidence for vitamin D) at high doses have been reported to be beneficial to the microbiome, both by increasing the diversity and the abundance of SCFA-producing bacteria and other beneficial bacteria and by maintaining the intestinal barrier.

In summary, given the effects of various TCM and herbal combinations on the gut microbiome, it is likely that TCM could be a candidate to target neurodegenerative and neurodevelopmental diseases.

### 8.6. Targeted Antibiotics

Antibiotics are prescribed to kill or suppress pathogenic bacteria during infections and generally have an adverse effect on the microbiota, including in the gut, where potential pathogens are often increased and SCFA-producing bacteria decreased [462]. This can lead to negative health effects, such as *C. difficile* infection and potentially AD [273], PD [259], and ASD [288]. Even after antibiotics are terminated, the microbiome may take months to recover, and the post-antibiotic microbiome can be different from the pre-antibiotic microbiome [463].

There are, however, a small number of antibiotics that do not follow this same trend and are sometimes called “eubiotics” [464]. For example, Nitrofurantoin, the broad-spectrum antibiotic used for urinary tract infections, has been shown to increase *Bifidobacterium* and *Faecalibacterium* in the gut [465,466]. Rifaximin is a poorly absorbed antibiotic, and, because its antimicrobial effect is increased with bile acids, it is more effective in the small intestine than in the colon [467]. Rifaximin also downregulates inflammation by reducing pro-inflammatory cytokines, improves the intestinal barrier, and does not reduce gut microbiome diversity [467]. In fact, rifaximin has been shown to have a positive effect on the gut microbiome [468], increasing the numbers of *Bifidobacterium*, *Lactobacillus*, and *Faecalibacterium* [467]. Originally prescribed for traveller’s diarrhoea, Rifaximin is now the antibiotic of choice for SIBO [469,470] and has been shown to be effective in treating moderately active Crohn’s disease [471], ulcerative colitis, and some IBS symptoms [470]. It may therefore have some role in the treatment of neurodegenerative and neurodevelopmental diseases, especially in the prodromal stages. Rifaximin has been shown to improve PD symptoms in mice, modulate the microbiome, and improve inflammatory markers. However, in a small (n = 9) human PD trial, the only change was an increase in *Flavonifractor*, previously identified as increased in PD [472]. In AD, rifaximin was shown in a small (n = 10) trial to increase the proportion of Phylum Firmicutes and reduce serum neurofilament-light and serum phosphorylated tau, but with no changes in cognition [473].

Vancomycin is a narrow-spectrum antibiotic active against Gram +ve bacteria (such as *Staphylococcus* and *Clostridium*) and is poorly absorbed. It reduces microbiome diversity and alters the microbiome composition, reducing SCFA production and the conversion of bile acids. While not a eubiotic, vancomycin has been demonstrated to have positive effects on some aspects of the gut microbiome. Vancomycin has been shown to increase *Akkermansia* numbers and prevent the onset of diabetes in a diabetic mouse model [464]. It has also been reported that symptoms of ASD could be reversed with the administration of vancomycin, although these gains were largely lost over time [474].

A number of animal models have demonstrated the potential of β-lactam antibiotics (such as Ceftriaxone) to treat ALS [475], MS [476], PD [477], AD [478,479], and age-related senescence [480].

In summary, while there may be some potential for the use of specific antibiotics to treat neurodegenerative and neurodevelopmental diseases, more work is needed to demonstrate the effectiveness of targeted antibiotic therapy and to develop antibiotics specific for particular bacterial targets.

### 8.7. Photobiomodulation

Photobiomodulation (PBM) therapy is the use of narrow-wavelength bands of non-thermal light (LED or laser) to modulate cellular responses. PBM targets molecules that absorb light (chromophores), especially cytochrome-C-oxidase in the mitochondria [481], which increases ATP production, releases reactive oxygen species (ROS), and promotes increased mitochondrial membrane potential, as well downstream cellular signalling, including gene transcription [481,482]. It was demonstrated in a systematic review in 2009 that PBM was effective for the treatment of chronic neck pain [483], with the formation of varicosities in the neurons that effectively blocked mitochondrial transport and hence the nerve conduction of pain [484]. PBM has a multitude of effects on the body due to its action at the molecular, mitochondrial, and cellular levels. One of the main effects of PBM is anti-inflammatory [485]. PBM therapy has been shown over many decades to be safe and free of serious deleterious side effects and is non-invasive.

Few studies have investigated the effects of PBM on the gut microbiome. Experiments have demonstrated the effectiveness of PBM treatment for the symptoms and neurology of Parkinson’s disease in animal models, either when light was directed to the head [486,487,488] or when it was directed to the body [489,490,491]. This improvement was seen even when the head was shielded from the light [492]. PBM directed to the abdomen has been shown to improve the microbiome in a wavelength-dependant manner [347], with significant differences between the sham and two different wavelength groups (660 nm and 808 nm) and increases in the SCFA-producing bacteria *Allobaculum*. Changes in the microbiome have since been repeated (unpublished) and have also been demonstrated in other studies. In a mouse model of AD [493], mid-infrared light over the entire body for 1 h per day for 6 weeks reversed dysbiosis in the microbiome in AD mice to that of the wild-type microbiome, including increases in *Akkermansia*. A second mouse model of AD [494] using red and infrared LEDs directed to the abdomen for 1000 s, 5 days per week for 8 weeks, resulted in the reversal of AD dysbiosis, including decreased *Helicobacter*, a genus previously identified as a risk factor for AD [495]. In a rat model of bone health [496], 30 min of infrared-light supplementing natural light every day for 12 weeks resulted in increased numbers of SCFA-producing bacteria and decreased numbers of *Desulfovibrio* (H_2_S-producing bacteria) compared to sham. In a rat model of T2DM, PBM was administered with an endoscopic fibre-optic light source directed to the duodenal mucosa. The treatment consisted of red light for 600 s and infrared light for 100 s. The result was the enrichment of a number of genera, including the SCFA producers *Allobaculum* and *Faecalibacterium* [497].

In human trials, only two studies have been published thus far. In a small case series for the treatment of PD with PBM, 12 participants were treated with transcranial LED (830 nm) and an abdominal laser (904 nm) for 20 min, three times per week [498]. Participants showed a significant improvement in the Firmicutes–Bacteroidetes ratio and a (non-significant) trend of increased numbers in some Bacteroidetes species (e.g., *Bacteroides* species, *Odoribacter*) and decreased numbers of Proteobacteria species and potential pathogens (e.g., *Methanobrevibacter*, *Enterococcus*, *Eggerthella*, and *Paraeggerthella*). This was accompanied by improvements in many of the symptoms of PD [499]. The treatment of a second group of participants [500] with a laser to the abdomen gave similar results (unpublished). Interestingly, in both studies, *Akkermansia*, *Lactobacillus*, and *Bifidobacterium* did not show any trends towards an increase or a decrease, and some of the most notable SCFA producers (*Faecalibacterium, Roseburia*, etc.) also did not increase.

The second published report was a single case study of a cancer therapy patient with IBS, who showed a significant improvement in diversity and significant improvements in many of the bacterial markers of a healthy microbiome following PBM therapy, including increased *Akkermansia*, *Faecalibacterium*, and *Roseburia* and decreased potential pathogens *Eggerthella, Paraeggerthella, Collinsella*, and *Streptococcus*.

PBM has the potential to modulate the microbiome in a number of diseases that have a strong MGBA connection, including neurodegenerative diseases, neurodevelopmental diseases, and neuropsychiatric disorders. This also includes diseases with a channelopathy basis, such as epilepsy, migraine with aura headache, and pain channelopathies. Recent studies of the effect of PBM in pre-clinical models of epilepsy indicated good results [501], as well as the treatment of other diseases with a channelopathy link, such as chronic intractable headache with migrainous features [502]. PBM has been proposed as a treatment for diabetic kidney disease [503], also potentially acting via the MGBA. PBM has also been proposed as a treatment for a number of neurological diseases based on pre-clinical studies and small clinical trials, including depression and anxiety [504], MS [505], ASD [506], long COVID with brain fog [507], stroke [508], AD [509], neuropsychiatric disorders [510], and TBI [511], all of which have an MGBA involvement. It would be informative to understand the extent to which the microbiome is also modified by PBM in these treatments.

In summary, targeting the abdomen with PBM has been shown, in some animal models and in limited numbers of humans, to modify the microbiome and so may be a mechanism of modulating a number of intractable neurological diseases and conditions using the MGBA connection.

## 9. Conclusions

The human gut microbiome contains the largest number of bacteria in the body and has the potential to greatly influence metabolism, not only locally but also systemically. The gut microbiome can become unbalanced (dysbiosis) through dietary changes, medication use, lifestyle choices, environmental factors, and ageing. There is an established link between a healthy, balanced, and diverse microbiome and overall health, with gut dysbiosis related to many diseases, including lifestyle diseases, metabolic diseases, inflammatory diseases, and neurological diseases. While this link is largely an association between the MGBA and human disease, in animal models, a causative link can be demonstrated. The link between the gut and the brain is particularly important in maintaining brain health, with a strong association between dysbiosis in the gut and brain disorders. This suggests not only that the gut microbiota composition can be used to make an early diagnosis of neurodegenerative and neurodevelopmental disease but also that modifying the gut microbiome to influence the MGBA might present a therapeutic target for diseases that have proved intractable, with the aim of altering the trajectory of neurodegenerative and neurodevelopmental diseases. While conventional methods of altering the microbiome, such as diet, prebiotics, and probiotics, have shown some potential in animal models, results in human trials have been less convincing. Long-term modification of the diet to encourage a healthier microbiome has been shown to reduce the risk of developing neurodegenerative diseases, but the use of these techniques to alter the disease trajectory after diagnosis has been less promising. There is perhaps more opportunity to make a positive change to the microbiome with less established techniques, such as targeted medications, FMT, TCM, and PBM. In particular, FMT and PBM show promise not only in altering the microbiome but also in improving the symptoms of neurogenerative and neurodevelopmental diseases. What is unclear, is the permanency of any changes that can be made to the microbiome, that is, whether these changes are transient, semi-permanent or permanent, and how often these treatments might need to be repeated to maintain a healthy microbiome. Perhaps, a fruitful area of future research is a combination of these novel therapies to target neurodegenerative and neurodevelopmental diseases.

## Data Availability

All data are contained within this manuscript.

## References

[1] Walker R.W., Clemente J.C., Peter I., Loos R.J. (2017). The prenatal gut microbiome: Are we colonized with bacteria in utero?. Pediatr. Obes..

[2] Leinwand J.C., Paul B., Chen R., Xu F., Sierra M.A., Paluru M.M., Nanduri S., Alcantara C.G., Shadaloey S.A., Yang F. (2022). Intrahepatic microbes govern liver immunity by programming NKT cells. J. Clin. Investig..

[3] Castillo D.J., Rifkin R.F., Cowan D.A., Potgieter M. (2019). The Healthy Human Blood Microbiome: Fact or Fiction?. Front. Cell. Infect. Microbiol..

[4] Link C.D. (2021). Is there a brain microbiome?. Neurosci. Insights.

[5] Baquero F., Nombela C. (2012). The microbiome as a human organ. Clin. Microbiol. Infect..

[6] Sender R., Fuchs S., Milo R. (2016). Revised estimates for the number of human and bacteria cells in the body. PLoS Biol..

[7] Reyes A., Semenkovich N.P., Whiteson K., Rohwer F., Gordon J.I. (2012). Going viral: Next-generation sequencing applied to phage populations in the human gut. Nat. Rev. Microbiol..

[8] Tierney B.T., Yang Z., Luber J.M., Beaudin M., Wibowo M.C., Baek C., Mehlenbacher E., Patel C.J., Kostic A.D. (2019). The Landscape of Genetic Content in the Gut and Oral Human Microbiome. Cell Host Microbe.

[9] Qin J., Li R., Raes J., Arumugam M., Burgdorf K.S., Manichanh C., Nielsen T., Pons N., Levenez F., Yamada T. (2010). A human gut microbial gene catalogue established by metagenomic sequencing. Nature.

[10] Aebersold R., Agar J.N., Amster I.J., Baker M.S., Bertozzi C.R., Boja E.S., Costello C.E., Cravatt B.F., Fenselau C., Garcia B.A. (2018). How many human proteoforms are there?. Nat. Chem. Biol..

[11] Miller A.L., Bessho S., Grando K., Tükel Ç. (2021). Microbiome or Infections: Amyloid-Containing Biofilms as a Trigger for Complex Human Diseases. Front. Immunol..

[12] Levitan O., Johnson D.A., Macg M. (2020). The Inner-Colonic Bacterial Communities of the Human Gut are Unique and Profoundly Different than those of Stool Samples. Am. J. Gastroenterol..

[13] Chong C.Y.L., Bloomfield F.H., O’Sullivan J.M. (2018). Factors affecting gastrointestinal microbiome development in neonates. Nutrients.

[14] Dominguez-Bello M.G., Godoy-Vitorino F., Knight R., Blaser M.J. (2019). Role of the microbiome in human development. Gut.

[15] Spor A., Koren O., Ley R. (2011). Unravelling the effects of the environment and host genotype on the gut microbiome. Nat. Rev. Microbiol..

[16] Lu M., Wang Z., Wang Z. (2018). Microbiota and aging. Aging and Aging-Related Diseases: Mechanisms and Interventions.

[17] Nandwana V., Nandwana N.K., Das Y., Saito M., Panda T., Das S., Almaguel F., Hosmane N.S., Das B.C. (2022). The role of microbiome in brain development and neurodegenerative diseases. Molecules.

[18] Goodrich J.K., Davenport E.R., Beaumont M., Jackson M.A., Knight R., Ober C., Spector T.D., Bell J.T., Clark A.G., Ley R.E. (2016). Genetic determinants of the gut microbiome in UK twins. Cell Host Microbe.

[19] Falony G., Joossens M., Vieira-Silva S., Wang J., Darzi Y., Faust K., Kurilshikov A., Bonder M.J., Valles-Colomer M., Vandeputte D. (2016). Population-level analysis of gut microbiome variation. Science.

[20] King C.H., Desai H., Sylvetsky A.C., LoTempio J., Ayanyan S., Carrie J., Crandall K.A., Fochtman B.C., Gasparyan L., Gulzar N. (2019). Baseline human gut microbiota profile in healthy people and standard reporting template. PLoS ONE.

[21] Manor O., Dai C.L., Kornilov S.A., Smith B., Price N.D., Lovejoy J.C., Gibbons S.M., Magis A.T. (2020). Health and disease markers correlate with gut microbiome composition across thousands of people. Nat. Commun..

[22] Vaiserman A., Romanenko M., Piven L., Moseiko V., Lushchak O., Kryzhanovska N., Guryanov V., Koliada A. (2020). Differences in the gut Firmicutes to Bacteroidetes ratio across age groups in healthy Ukrainian population. BMC Microbiol..

[23] Liang D., Leung R.K.-K., Guan W., Au W.W. (2018). Involvement of gut microbiome in human health and disease: Brief overview, knowledge gaps and research opportunities. Gut Pathog..

[24] Magne F., Gotteland M., Gauthier L., Zazueta A., Pesoa S., Navarrete P., Balamurugan R. (2020). The Firmicutes/Bacteroidetes Ratio: A Relevant Marker of Gut Dysbiosis in Obese Patients?. Nutrients.

[25] Knights D., Ward T.L., McKinlay C.E., Miller H., Gonzalez A., McDonald D., Knight R. (2014). Rethinking “enterotypes”. Cell Host Microbe.

[26] Cirstea M., Radisavljevic N., Finlay B.B. (2018). Good bug, bad bug: Breaking through microbial stereotypes. Cell Host Microbe.

[27] Maskarinec G., Hullar M.A.J., Monroe K.R., Shepherd J.A., Hunt J., Randolph T.W., Wilkens L.R., Boushey C.J., Le Marchand L., Lim U. (2019). Fecal Microbial Diversity and Structure Are Associated with Diet Quality in the Multiethnic Cohort Adiposity Phenotype Study. J. Nutr..

[28] Nogal B., Blumberg J.B., Blander G., Jorge M. (2021). Gut Microbiota–Informed Precision Nutrition in the Generally Healthy Individual: Are We There Yet?. Curr. Dev. Nutr..

[29] Shi Z. (2019). Gut Microbiota: An Important Link between Western Diet and Chronic Diseases. Nutrients.

[30] De Filippo C., Cavalieri D., Di Paola M., Ramazzotti M., Poullet J.B., Massart S., Collini S., Pieraccini G., Lionetti P. (2010). Impact of diet in shaping gut microbiota revealed by a comparative study in children from Europe and rural Africa. Proc. Natl. Acad. Sci. USA.

[31] Clooney A.G., Eckenberger J., Laserna-Mendieta E., Sexton K.A., Bernstein M.T., Vagianos K., Sargent M., Ryan F.J., Moran C., Sheehan D. (2021). Ranking microbiome variance in inflammatory bowel disease: A large longitudinal intercontinental study. Gut.

[32] Metwaly A., Reitmeier S., Haller D. (2022). Microbiome risk profiles as biomarkers for inflammatory and metabolic disorders. Nat. Rev. Gastroenterol. Hepatol..

[33] Bolte L.A., Vich Vila A., Imhann F., Collij V., Gacesa R., Peters V., Wijmenga C., Kurilshikov A., Campmans-Kuijpers M.J.E., Fu J. (2021). Long-term dietary patterns are associated with pro-inflammatory and anti-inflammatory features of the gut microbiome. Gut.

[34] Ahmad S., Moorthy M.V., Demler O.V., Hu F.B., Ridker P.M., Chasman D.I., Mora S. (2018). Assessment of Risk Factors and Biomarkers Associated With Risk of Cardiovascular Disease Among Women Consuming a Mediterranean Diet. JAMA Netw. Open.

[35] Guasch-Ferré M., Willett W.C. (2021). The Mediterranean diet and health: A comprehensive overview. J. Intern. Med..

[36] Shannon O.M., Ranson J.M., Gregory S., Macpherson H., Milte C., Lentjes M., Mulligan A., McEvoy C., Griffiths A., Matu J. (2023). Mediterranean diet adherence is associated with lower dementia risk, independent of genetic predisposition: Findings from the UK Biobank prospective cohort study. BMC Med..

[37] Jin Q., Black A., Kales S.N., Vattem D., Ruiz-Canela M., Sotos-Prieto M. (2019). Metabolomics and microbiomes as potential tools to evaluate the effects of the Mediterranean diet. Nutrients.

[38] Tomova A., Bukovsky I., Rembert E., Yonas W., Alwarith J., Barnard N.D., Kahleova H. (2019). The Effects of Vegetarian and Vegan Diets on Gut Microbiota. Front. Nutr..

[39] So D., Loughman A., Staudacher H.M. (2022). Effects of a low FODMAP diet on the colonic microbiome in irritable bowel syndrome: A systematic review with meta-analysis. Am. J. Clin. Nutr..

[40] Engen P.A., Green S.J., Voigt R.M., Forsyth C.B., Keshavarzian A. (2015). The Gastrointestinal Microbiome: Alcohol Effects on the Composition of Intestinal Microbiota. Alcohol. Res..

[41] Vijay A., Kouraki A., Gohir S., Turnbull J., Kelly A., Chapman V., Barrett D.A., Bulsiewicz W.J., Valdes A.M. (2021). The anti-inflammatory effect of bacterial short chain fatty acids is partially mediated by endocannabinoids. Gut Microbes.

[42] Shin J.-H., Kim C.-S., Cha L., Kim S., Lee S., Chae S., Chun W.Y., Shin D.-M. (2022). Consumption of 85% cocoa dark chocolate improves mood in association with gut microbial changes in healthy adults: A randomized controlled trial. J. Nutr. Biochem..

[43] Camps-Bossacoma M., Massot-Cladera M., Pérez-Cano F.J., Castell M., Watson R.R., Preedy V.R. (2019). Chapter 18-Influence of a Cocoa-Enriched Diet on the Intestinal Immune System and Microbiota. Dietary Interventions in Gastrointestinal Diseases.

[44] Shivakoti R., Biggs M.L., Djoussé L., Durda P.J., Kizer J.R., Psaty B., Reiner A.P., Tracy R.P., Siscovick D., Mukamal K.J. (2022). Intake and Sources of Dietary Fiber, Inflammation, and Cardiovascular Disease in Older US Adults. JAMA Netw. Open.

[45] Acosta S., Johansson A., Drake I. (2021). Diet and Lifestyle Factors and Risk of Atherosclerotic Cardiovascular Disease—A Prospective Cohort Study. Nutrients.

[46] Kyrø C., Tjønneland A., Overvad K., Olsen A., Landberg R. (2018). Higher Whole-Grain Intake Is Associated with Lower Risk of Type 2 Diabetes among Middle-Aged Men and Women: The Danish Diet, Cancer, and Health Cohort. J. Nutr..

[47] Daïen C.I., Pinget G.V., Tan J.K., Macia L. (2017). Detrimental Impact of Microbiota-Accessible Carbohydrate-Deprived Diet on Gut and Immune Homeostasis: An Overview. Front. Immunol..

[48] Sonnenburg E.D., Sonnenburg J.L. (2014). Starving our Microbial Self: The Deleterious Consequences of a Diet Deficient in Microbiota-Accessible Carbohydrates. Cell Metab..

[49] Wagenaar C.A., van de Put M., Bisschops M., Walrabenstein W., de Jonge C.S., Herrema H., van Schaardenburg D. (2021). The Effect of Dietary Interventions on Chronic Inflammatory Diseases in Relation to the Microbiome: A Systematic Review. Nutrients.

[50] Rubin K.H., Rasmussen N.F., Petersen I., Kopp T.I., Stenager E., Magyari M., Hetland M.L., Bygum A., Glintborg B., Andersen V. (2020). Intake of dietary fibre, red and processed meat and risk of late-onset Chronic Inflammatory Diseases: A prospective Danish study on the “diet, cancer and health” cohort. Int. J. Med. Sci..

[51] Ford A.L., Nagulesapillai V., Piano A., Auger J., Girard S.-A., Christman M., Tompkins T.A., Dahl W.J. (2020). Microbiota stability and gastrointestinal tolerance in response to a high-protein diet with and without a prebiotic, probiotic, and synbiotic: A randomized, double-blind, placebo-controlled trial in older women. J. Acad. Nutr. Diet..

[52] Ma N., Tian Y., Wu Y., Ma X. (2017). Contributions of the Interaction Between Dietary Protein and Gut Microbiota to Intestinal Health. Curr. Protein Pept. Sci..

[53] Cai J., Chen Z., Wu W., Lin Q., Liang Y. (2022). High animal protein diet and gut microbiota in human health. Crit. Rev. Food Sci. Nutr..

[54] Murphy E.A., Velazquez K.T., Herbert K.M. (2015). Influence of high-fat diet on gut microbiota: A driving force for chronic disease risk. Curr. Opin. Clin. Nutr. Metab. Care.

[55] Wan Y., Wang F., Yuan J., Li J., Jiang D., Zhang J., Li H., Wang R., Tang J., Huang T. (2019). Effects of dietary fat on gut microbiota and faecal metabolites, and their relationship with cardiometabolic risk factors: A 6-month randomised controlled-feeding trial. Gut.

[56] Satokari R. (2020). High Intake of Sugar and the Balance between Pro- and Anti-Inflammatory Gut Bacteria. Nutrients.

[57] Olson C.A., Vuong H.E., Yano J.M., Liang Q.Y., Nusbaum D.J., Hsiao E.Y. (2018). The gut microbiota mediates the anti-seizure effects of the ketogenic diet. Cell.

[58] Basciani S., Camajani E., Contini S., Persichetti A., Risi R., Bertoldi L., Strigari L., Prossomariti G., Watanabe M., Mariani S. (2020). Very-low-calorie ketogenic diets with whey, vegetable, or animal protein in patients with obesity: A randomized pilot study. J. Clin. Endocrinol. Metab..

[59] Zhu L.B., Zhang Y.C., Huang H.H., Lin J. (2021). Prospects for clinical applications of butyrate-producing bacteria. World J. Clin. Pediatr..

[60] Elce A., Amato F., Zarrilli F., Calignano A., Troncone R., Castaldo G., Canani R. (2017). Butyrate modulating effects on pro-inflammatory pathways in human intestinal epithelial cells. Benef. Microbes.

[61] van der Beek C.M., Dejong C.H., Troost F.J., Masclee A.A., Lenaerts K. (2017). Role of short-chain fatty acids in colonic inflammation, carcinogenesis, and mucosal protection and healing. Nutr. Rev..

[62] Krautkramer K.A., Fan J., Bäckhed F. (2021). Gut microbial metabolites as multi-kingdom intermediates. Nat. Rev. Microbiol..

[63] Larabi A.B., Masson H.L., Bäumler A.J. (2023). Bile acids as modulators of gut microbiota composition and function. Gut Microbes.

[64] Fiorucci S., Biagioli M., Zampella A., Distrutti E. (2018). Bile acids activated receptors regulate innate immunity. Front. Immunol..

[65] Aguirre A.M., Yalcinkaya N., Wu Q., Swennes A., Tessier M.E., Roberts P., Miyajima F., Savidge T., Sorg J.A. (2021). Bile acid-independent protection against Clostridioides difficile infection. PLoS Pathog..

[66] Yaghoubfar R., Behrouzi A., Ashrafian F., Shahryari A., Moradi H.R., Choopani S., Hadifar S., Vaziri F., Nojoumi S.A., Fateh A. (2020). Modulation of serotonin signaling/metabolism by Akkermansia muciniphila and its extracellular vesicles through the gut-brain axis in mice. Sci. Rep..

[67] Tomasova L., Grman M., Ondrias K., Ufnal M. (2021). The impact of gut microbiota metabolites on cellular bioenergetics and cardiometabolic health. Nutr. Metab..

[68] Varesi A., Pierella E., Romeo M., Piccini G.B., Alfano C., Bjørklund G., Oppong A., Ricevuti G., Esposito C., Chirumbolo S. (2022). The Potential Role of Gut Microbiota in Alzheimer’s Disease: From Diagnosis to Treatment. Nutrients.

[69] Gershon M.D., Tack J. (2007). The serotonin signaling system: From basic understanding to drug development for functional GI disorders. Gastroenterology.

[70] Tennoune N., Andriamihaja M., Blachier F. (2022). Production of Indole and Indole-Related Compounds by the Intestinal Microbiota and Consequences for the Host: The Good, the Bad, and the Ugly. Microorganisms.

[71] Benech N., Rolhion N., Sokol H. (2021). Tryptophan metabolites get the gut moving. Cell Host Microbe.

[72] Hajsl M., Hlavackova A., Broulikova K., Sramek M., Maly M., Dyr J.E., Suttnar J. (2020). Tryptophan Metabolism, Inflammation, and Oxidative Stress in Patients with Neurovascular Disease. Metabolites.

[73] Rankin R., Kaiser K.A., de los Santos-Alexis K., Park H., Uhlemann A.-C., DHD G., Arpaia N. (2023). Dietary tryptophan deficiency promotes gut RORgt+ Treg cells at the expense of Gata3+ Treg cells and alters commensal microbiota metabolism. Cell Rep..

[74] Lawley T.D., Walker A.W. (2013). Intestinal colonization resistance. Immunology.

[75] Glover J.S., Ticer T.D., Engevik M.A. (2022). Characterizing the mucin-degrading capacity of the human gut microbiota. Sci. Rep..

[76] Naito Y., Uchiyama K., Takagi T. (2018). A next-generation beneficial microbe: Akkermansia muciniphila. J. Clin. Biochem. Nutr..

[77] Reinoso Webb C., Koboziev I., Furr K.L., Grisham M.B. (2016). Protective and pro-inflammatory roles of intestinal bacteria. Pathophysiology.

[78] Wiertsema S.P., van Bergenhenegouwen J., Garssen J., Knippels L.M.J. (2021). The Interplay between the Gut Microbiome and the Immune System in the Context of Infectious Diseases throughout Life and the Role of Nutrition in Optimizing Treatment Strategies. Nutrients.

[79] Zheng D., Liwinski T., Elinav E. (2020). Interaction between microbiota and immunity in health and disease. Cell Res..

[80] Gensollen T., Iyer S.S., Kasper D.L., Blumberg R.S. (2016). How colonization by microbiota in early life shapes the immune system. Science.

[81] Shi N., Li N., Duan X., Niu H. (2017). Interaction between the gut microbiome and mucosal immune system. Mil. Med. Res..

[82] Kelly J.R., Kennedy P.J., Cryan J.F., Dinan T.G., Clarke G., Hyland N.P. (2015). Breaking down the barriers: The gut microbiome, intestinal permeability and stress-related psychiatric disorders. Front. Cell. Neurosci..

[83] Buffington S.A., Dooling S.W., Sgritta M., Noecker C., Murillo O.D., Felice D.F., Turnbaugh P.J., Costa-Mattioli M. (2021). Dissecting the contribution of host genetics and the microbiome in complex behaviors. Cell.

[84] Sun J., Xu J., Ling Y., Wang F., Gong T., Yang C., Ye S., Ye K., Wei D., Song Z. (2019). Fecal microbiota transplantation alleviated Alzheimer’s disease-like pathogenesis in APP/PS1 transgenic mice. Transl. Psychiatry.

[85] Suganya K., Koo B.S. (2020). Gut-Brain Axis: Role of Gut Microbiota on Neurological Disorders and How Probiotics/Prebiotics Beneficially Modulate Microbial and Immune Pathways to Improve Brain Functions. Int. J. Mol. Sci..

[86] Treangen T.J., Wagner J., Burns M.P., Villapol S. (2018). Traumatic brain injury in mice induces acute bacterial dysbiosis within the fecal microbiome. Front. Immunol..

[87] Diaz Heijtz R., Wang S., Anuar F., Qian Y., Björkholm B., Samuelsson A., Hibberd M.L., Forssberg H., Pettersson S. (2011). Normal gut microbiota modulates brain development and behavior. Proc. Natl. Acad. Sci. USA.

[88] Bravo J.A., Forsythe P., Chew M.V., Escaravage E., Savignac H.M., Dinan T.G., Bienenstock J., Cryan J.F. (2011). Ingestion of Lactobacillus strain regulates emotional behavior and central GABA receptor expression in a mouse via the vagus nerve. Proc. Natl. Acad. Sci. USA.

[89] Sgritta M., Dooling S.W., Buffington S.A., Momin E.N., Francis M.B., Britton R.A., Costa-Mattioli M. (2019). Mechanisms underlying microbial-mediated changes in social behavior in mouse models of autism spectrum disorder. Neuron.

[90] Rogers G., Keating D.J., Young R.L., Wong M.-L., Licinio J., Wesselingh S. (2016). From gut dysbiosis to altered brain function and mental illness: Mechanisms and pathways. Mol. Psychiatry.

[91] Labanski A., Langhorst J., Engler H., Elsenbruch S. (2020). Stress and the brain-gut axis in functional and chronic-inflammatory gastrointestinal diseases: A transdisciplinary challenge. Psychoneuroendocrinology.

[92] Schneider K.M., Blank N., Alvarez Y., Thum K., Lundgren P., Litichevskiy L., Sleeman M., Bahnsen K., Kim J., Kardo S. (2023). The enteric nervous system relays psychological stress to intestinal inflammation. Cell.

[93] Almand A.T., Anderson A.P., Hitt B.D., Sitko J.C., Joy R.M., Easter B.D., Almand E.A. (2022). The influence of perceived stress on the human microbiome. BMC Res. Notes.

[94] Chen S.-J., Lin C.-H. (2022). Gut microenvironmental changes as a potential trigger in Parkinson’s disease through the gut–brain axis. J. Biomed. Sci..

[95] Chavan S.S., Pavlov V.A., Tracey K.J. (2017). Mechanisms and Therapeutic Relevance of Neuro-immune Communication. Immunity.

[96] Calarge C.A., Devaraj S., Shulman R.J. (2019). Gut permeability and depressive symptom severity in unmedicated adolescents. J. Affect. Disord..

[97] Bonaz B., Bazin T., Pellissier S. (2018). The Vagus Nerve at the Interface of the Microbiota-Gut-Brain Axis. Front. Neurosci..

[98] Tang W., Zhu H., Feng Y., Guo R., Wan D. (2020). The Impact of Gut Microbiota Disorders on the Blood-Brain Barrier. Infect. Drug Resist..

[99] Kim Y., Keogh J., Clifton P. (2018). Probiotics, prebiotics, synbiotics and insulin sensitivity. Nutr. Res. Rev..

[100] Farzi A., Fröhlich E.E., Holzer P. (2018). Gut microbiota and the neuroendocrine system. Neurotherapeutics.

[101] Zhu Y., Li Y., Zhang Q., Song Y., Wang L., Zhu Z. (2022). Interactions Between Intestinal Microbiota and Neural Mitochondria: A New Perspective on Communicating Pathway From Gut to Brain. Front. Microbiol..

[102] Borre Y.E., O’Keeffe G.W., Clarke G., Stanton C., Dinan T.G., Cryan J.F. (2014). Microbiota and neurodevelopmental windows: Implications for brain disorders. Trends Mol. Med..

[103] Cryan J.F., O’Riordan K.J., Sandhu K., Peterson V., Dinan T.G. (2020). The gut microbiome in neurological disorders. Lancet Neurol..

[104] Luczynski P., Whelan S.O., O’Sullivan C., Clarke G., Shanahan F., Dinan T.G., Cryan J.F. (2016). Adult microbiota-deficient mice have distinct dendritic morphological changes: Differential effects in the amygdala and hippocampus. Eur. J. Neurosci..

[105] Collins J., Borojevic R., Verdu E., Huizinga J., Ratcliffe E. (2014). Intestinal microbiota influence the early postnatal development of the enteric nervous system. Neurogastroenterol. Motil..

[106] de Weerth C. (2017). Do bacteria shape our development? Crosstalk between intestinal microbiota and HPA axis. Neurosci. Biobehav. Rev..

[107] Cussotto S., Sandhu K.V., Dinan T.G., Cryan J.F. (2018). The neuroendocrinology of the microbiota-gut-brain axis: A behavioural perspective. Front. Neuroendocrinol..

[108] Taniya M.A., Chung H.J., Al Mamun A., Alam S., Aziz M., Emon N.U., Islam M., Hong S.S., Podder B.R., Ara Mimi A. (2022). Role of Gut Microbiome in Autism Spectrum Disorder and Its Therapeutic Regulation. Front. Cell Infect. Microbiol..

[109] Damiani F., Cornuti S., Tognini P. (2023). The gut-brain connection: Exploring the influence of the gut microbiota on neuroplasticity and neurodevelopmental disorders. Neuropharmacology.

[110] Komaroff A.L. (2017). The microbiome and risk for obesity and diabetes. Jama.

[111] Lee C.J., Sears C.L., Maruthur N. (2020). Gut microbiome and its role in obesity and insulin resistance. Ann. N. Y. Acad. Sci..

[112] Glassner K.L., Abraham B.P., Quigley E.M. (2020). The microbiome and inflammatory bowel disease. J. Allergy Clin. Immunol..

[113] Khan I., Ullah N., Zha L., Bai Y., Khan A., Zhao T., Che T., Zhang C. (2019). Alteration of gut microbiota in inflammatory bowel disease (IBD): Cause or consequence? IBD treatment targeting the gut microbiome. Pathogens.

[114] Lee M., Chang E.B. (2021). Inflammatory Bowel Diseases (IBD) and the microbiome—Searching the crime scene for clues. Gastroenterology.

[115] Chong P.P., Chin V.K., Looi C.Y., Wong W.F., Madhavan P., Yong V.C. (2019). The microbiome and irritable bowel syndrome–a review on the pathophysiology, current research and future therapy. Front. Microbiol..

[116] Menees S., Chey W. (2018). The gut microbiome and irritable bowel syndrome. F1000Research.

[117] Li Q., Chang Y., Zhang K., Chen H., Tao S., Zhang Z. (2020). Implication of the gut microbiome composition of type 2 diabetic patients from northern China. Sci. Rep..

[118] Dabke K., Hendrick G., Devkota S. (2019). The gut microbiome and metabolic syndrome. J. Clin. Investig..

[119] Sharma S., Tripathi P. (2019). Gut microbiome and type 2 diabetes: Where we are and where to go?. J. Nutr. Biochem..

[120] Singer-Englar T., Barlow G., Mathur R. (2019). Obesity, diabetes, and the gut microbiome: An updated review. Expert Rev. Gastroenterol. Hepatol..

[121] Zhu T., Goodarzi M.O. (2020). Metabolites linking the gut microbiome with risk for type 2 diabetes. Curr. Nutr. Rep..

[122] Sikalidis A.K., Maykish A. (2020). The Gut Microbiome and Type 2 Diabetes Mellitus: Discussing A Complex Relationship. Biomedicines.

[123] Bodkhe R., Balakrishnan B., Taneja V. (2019). The role of microbiome in rheumatoid arthritis treatment. Adv. Musculoskelet. Dis..

[124] Kim S., Goel R., Kumar A., Qi Y., Lobaton G., Hosaka K., Mohammed M., Handberg E.M., Richards E.M., Pepine C.J. (2018). Imbalance of gut microbiome and intestinal epithelial barrier dysfunction in patients with high blood pressure. Clin. Sci..

[125] Jin M., Qian Z., Yin J., Xu W., Zhou X. (2019). The role of intestinal microbiota in cardiovascular disease. J. Cell. Mol. Med..

[126] Peng J., Xiao X., Hu M., Zhang X. (2018). Interaction between gut microbiome and cardiovascular disease. Life Sci..

[127] Yoshida N., Yamashita T., Hirata K.-i. (2018). Gut microbiome and cardiovascular diseases. Diseases.

[128] Tang W.W., Kitai T., Hazen S.L. (2017). Gut microbiota in cardiovascular health and disease. Circ. Res..

[129] Guo M., Wang H., Xu S., Zhuang Y., An J., Su C., Xia Y., Chen J., Xu Z.Z., Liu Q. (2020). Alteration in gut microbiota is associated with dysregulation of cytokines and glucocorticoid therapy in systemic lupus erythematosus. Gut Microbes.

[130] Safari Z., Gérard P. (2019). The links between the gut microbiome and non-alcoholic fatty liver disease (NAFLD). Cell. Mol. Life Sci..

[131] Grosicki G.J., Fielding R.A., Lustgarten M.S. (2018). Gut Microbiota Contribute to Age-Related Changes in Skeletal Muscle Size, Composition, and Function: Biological Basis for a Gut-Muscle Axis. Calcif. Tissue Int..

[132] Casati M., Ferri E., Azzolino D., Cesari M., Arosio B. (2019). Gut microbiota and physical frailty through the mediation of sarcopenia. Exp. Gerontol..

[133] Leclercq S., Matamoros S., Cani P.D., Neyrinck A.M., Jamar F., Stärkel P., Windey K., Tremaroli V., Bäckhed F., Verbeke K. (2014). Intestinal permeability, gut-bacterial dysbiosis, and behavioral markers of alcohol-dependence severity. Proc. Natl. Acad. Sci. USA.

[134] Capuco A., Urits I., Hasoon J., Chun R., Gerald B., Wang J.K., Kassem H., Ngo A.L., Abd-Elsayed A., Simopoulos T. (2020). Current Perspectives on Gut Microbiome Dysbiosis and Depression. Adv. Ther..

[135] Simpson C.A., Diaz-Arteche C., Eliby D., Schwartz O.S., Simmons J.G., Cowan C.S.M. (2021). The gut microbiota in anxiety and depression–A systematic review. Clin. Psychol. Rev..

[136] Yang B., Wei J., Ju P., Chen J. (2019). Effects of regulating intestinal microbiota on anxiety symptoms: A systematic review. Gen. Psychiatr..

[137] Arzani M., Jahromi S.R., Ghorbani Z., Vahabizad F., Martelletti P., Ghaemi A., Sacco S., Togha M., School of Advanced Studies of the European Headache Federation (EHF-SAS) (2020). Gut-brain axis and migraine headache: A comprehensive review. J. Headache Pain.

[138] Shoubridge A.P., Choo J.M., Martin A.M., Keating D.J., Wong M.-L., Licinio J., Rogers G.B. (2022). The gut microbiome and mental health: Advances in research and emerging priorities. Mol. Psychiatry.

[139] Jiang X., Gu X., Zhou X., Chen X., Zhang X., Yang Y., Qin Y., Shen L., Yu W., Su D. (2019). Intestinal dysbacteriosis mediates the reference memory deficit induced by anaesthesia/surgery in aged mice. Brain Behav. Immun..

[140] Panther E.J., Dodd W., Clark A., Lucke-Wold B. (2022). Gastrointestinal microbiome and neurologic injury. Biomedicines.

[141] Meng C., Deng P., Miao R., Tang H., Li Y., Wang J., Wu J., Wang W., Liu S., Xia J. (2023). Gut Microbiome and Risk of Ischemic Stroke: A Comprehensive Mendelian Randomization Study. Eur. J. Prev. Cardiol..

[142] Felger J.C. (2019). Role of Inflammation in Depression and Treatment Implications. Handb. Exp. Pharm..

[143] Haran J.P., Bhattarai S.K., Foley S.E., Dutta P., Ward D.V., Bucci V., McCormick B.A. (2019). Alzheimer’s disease microbiome is associated with dysregulation of the anti-inflammatory P-glycoprotein pathway. mBio.

[144] Wasser C.I., Mercieca E.-C., Kong G., Hannan A.J., McKeown S.J., Glikmann-Johnston Y., Stout J.C. (2020). Gut dysbiosis in Huntington’s disease: Associations among gut microbiota, cognitive performance and clinical outcomes. Brain Commun..

[145] Dumitrescu L., Marta D., Dănău A., Lefter A., Tulbă D., Cozma L., Manole E., Gherghiceanu M., Ceafalan L.C., Popescu B.O. (2021). Serum and fecal markers of intestinal inflammation and intestinal barrier permeability are elevated in Parkinson’s disease. Front. Neurosci..

[146] Siniscalco D., Schultz S., Brigida A.L., Antonucci N. (2018). Inflammation and Neuro-Immune Dysregulations in Autism Spectrum Disorders. Pharmaceuticals.

[147] van den Munckhof I.C.L., Kurilshikov A., ter Horst R., Riksen N.P., Joosten L.A.B., Zhernakova A., Fu J., Keating S.T., Netea M.G., de Graaf J. (2018). Role of gut microbiota in chronic low-grade inflammation as potential driver for atherosclerotic cardiovascular disease: A systematic review of human studies. Obes. Rev..

[148] Liu F., Lee S.A., Riordan S.M., Zhang L., Zhu L. (2019). Effects of Anti-Cytokine Antibodies on Gut Barrier Function. Mediat. Inflamm.

[149] Braniste V., Al-Asmakh M., Kowal C., Anuar F., Abbaspour A., Tóth M., Korecka A., Bakocevic N., Ng L.G., Kundu P. (2014). The gut microbiota influences blood-brain barrier permeability in mice. Sci. Transl. Med..

[150] Sears C.L., Geis A.L., Housseau F. (2014). Bacteroides fragilis subverts mucosal biology: From symbiont to colon carcinogenesis. J. Clin. Investig..

[151] Beishuizen A., Thijs L.G. (2003). Endotoxin and the hypothalamo-pituitary-adrenal (HPA) axis. J. Endotoxin Res..

[152] Reichardt F., Chassaing B., Nezami B.G., Li G., Tabatabavakili S., Mwangi S., Uppal K., Liang B., Vijay-Kumar M., Jones D. (2017). Western diet induces colonic nitrergic myenteric neuropathy and dysmotility in mice via saturated fatty acid-and lipopolysaccharide-induced TLR4 signalling. J. Physiol..

[153] Keita å.v., Söderholm J.D. (2010). The intestinal barrier and its regulation by neuroimmune factors. Neurogastroenterol. Motil..

[154] Theoharides T.C., Konstantinidou A.D. (2007). Corticotropin-releasing hormone and the blood-brain-barrier. Front. Biosci.-Landmark.

[155] Zhang K., Wang N., Lu L., Ma X. (2020). Fermentation and Metabolism of Dietary Protein by Intestinal Microorganisms. Curr. Protein Pept. Sci..

[156] Zhao J., Zhang X., Liu H., Brown M.A., Qiao S. (2019). Dietary Protein and Gut Microbiota Composition and Function. Curr. Protein Pept. Sci..

[157] Velasquez M.T., Ramezani A., Manal A., Raj D.S. (2016). Trimethylamine N-Oxide: The Good, the Bad and the Unknown. Toxins.

[158] O’Mahony S.M., Clarke G., Borre Y., Dinan T.G., Cryan J. (2015). Serotonin, tryptophan metabolism and the brain-gut-microbiome axis. Behav. Brain Res..

[159] Alcock J., Carroll-Portillo A., Coffman C., Lin H.C. (2021). Evolution of human diet and microbiome-driven disease. Curr. Opin. Physiol..

[160] WHO (2021). Obesity and Overweight Fact Sheet.

[161] Vangay P., Johnson A.J., Ward T.L., Al-Ghalith G.A., Shields-Cutler R.R., Hillmann B.M., Lucas S.K., Beura L.K., Thompson E.A., Till L.M. (2018). US immigration westernizes the human gut microbiome. Cell.

[162] Juul F., Vaidean G., Parekh N. (2021). Ultra-processed Foods and Cardiovascular Diseases: Potential Mechanisms of Action. Adv. Nutr..

[163] Nettleton J.E., Reimer R.A., Shearer J. (2016). Reshaping the gut microbiota: Impact of low calorie sweeteners and the link to insulin resistance?. Physiol. Behav..

[164] Chassaing B., Van de Wiele T., De Bodt J., Marzorati M., Gewirtz A.T. (2017). Dietary emulsifiers directly alter human microbiota composition and gene expression ex vivo potentiating intestinal inflammation. Gut.

[165] Bana B., Cabreiro F. (2019). The Microbiome and Aging. Annu. Rev. Genet..

[166] Thevaranjan N., Puchta A., Schulz C., Naidoo A., Szamosi J.C., Verschoor C.P., Loukov D., Schenck L.P., Jury J., Foley K.P. (2017). Age-Associated Microbial Dysbiosis Promotes Intestinal Permeability, Systemic Inflammation, and Macrophage Dysfunction. Cell Host Microbe.

[167] Mariat D., Firmesse O., Levenez F., Guimarăes V., Sokol H., Doré J., Corthier G., Furet J.P. (2009). The Firmicutes/Bacteroidetes ratio of the human microbiota changes with age. BMC Microbiol..

[168] Hussain B., Fang C., Chang J. (2021). Blood–Brain Barrier Breakdown: An Emerging Biomarker of Cognitive Impairment in Normal Aging and Dementia. Front. Neurosci..

[169] Mitchell E., Davis A., Brass K., Dendinger M., Barner R., Gharaibeh R., Fodor A., Kavanagh K. (2017). Reduced intestinal motility, mucosal barrier function, and inflammation in aged monkeys. J. Nutr. Health Aging.

[170] Müller L., Di Benedetto S., Pawelec G., Harris J.R., Korolchuk V.I. (2019). The Immune System and Its Dysregulation with Aging. Biochemistry and Cell Biology of Ageing: Part II Clinical Science.

[171] De Maeyer R.P.H., Chambers E.S. (2021). The impact of ageing on monocytes and macrophages. Immunol. Lett..

[172] Conway J., Duggal N.A. (2021). Ageing of the gut microbiome: Potential influences on immune senescence and inflammageing. Ageing Res. Rev..

[173] Cryan J.F., O’Riordan K.J., Cowan C.S., Sandhu K.V., Bastiaanssen T.F., Boehme M., Codagnone M.G., Cussotto S., Fulling C., Golubeva A.V. (2019). The microbiota-gut-brain axis. Physiol. Rev..

[174] Shobeiri P., Kalantari A., Teixeira A.L., Rezaei N. (2022). Shedding light on biological sex differences and microbiota–gut–brain axis: A comprehensive review of its roles in neuropsychiatric disorders. Biol. Sex Differ..

[175] Ahn J., Hayes R.B. (2021). Environmental influences on the human microbiome and implications for noncommunicable disease. Annu. Rev. Public Health.

[176] Blum W.E., Zechmeister-Boltenstern S., Keiblinger K.M. (2019). Does soil contribute to the human gut microbiome?. Microorganisms.

[177] Sprockett D., Fukami T., Relman D.A. (2018). Role of priority effects in the early-life assembly of the gut microbiota. Nat. Rev. Gastroenterol. Hepatol..

[178] Wu J., Peters B.A., Dominianni C., Zhang Y., Pei Z., Yang L., Ma Y., Purdue M.P., Jacobs E.J., Gapstur S.M. (2016). Cigarette smoking and the oral microbiome in a large study of American adults. ISME J..

[179] Maier L., Typas A. (2017). Systematically investigating the impact of medication on the gut microbiome. Curr. Opin. Microbiol..

[180] Jalodia R., Abu Y.F., Oppenheimer M.R., Herlihy B., Meng J., Chupikova I., Tao J., Ghosh N., Dutta R.K., Kolli U. (2022). Opioid Use, Gut Dysbiosis, Inflammation, and the Nervous System. J. Neuroimmune Pharmacol..

[181] Strafella C., Caputo V., Galota M.R., Zampatti S., Marella G., Mauriello S., Cascella R., Giardina E. (2018). Application of Precision Medicine in Neurodegenerative Diseases. Front. Neurol..

[182] Vich Vila A., Collij V., Sanna S., Sinha T., Imhann F., Bourgonje A.R., Mujagic Z., Jonkers D.M.A.E., Masclee A.A.M., Fu J. (2020). Impact of commonly used drugs on the composition and metabolic function of the gut microbiota. Nat. Commun..

[183] Chen H.-Q., Gong J.-Y., Xing K., Liu M.-Z., Ren H., Luo J.-Q. (2022). Pharmacomicrobiomics: Exploiting the Drug-Microbiota Interactions in Antihypertensive Treatment. Front. Med..

[184] Weersma R.K., Zhernakova A., Fu J. (2020). Interaction between drugs and the gut microbiome. Gut.

[185] Iizumi T., Battaglia T., Ruiz V., Perez G.I.P. (2017). Gut microbiome and antibiotics. Arch. Med. Res..

[186] Konstantinidis T., Tsigalou C., Karvelas A., Stavropoulou E., Voidarou C., Bezirtzoglou E. (2020). Effects of antibiotics upon the gut microbiome: A review of the literature. Biomedicines.

[187] Langdon A., Crook N., Dantas G. (2016). The effects of antibiotics on the microbiome throughout development and alternative approaches for therapeutic modulation. Genome Med..

[188] Liu Y., Zhu X., Li R., Zhang J., Zhang F. (2020). Proton pump inhibitor utilisation and potentially inappropriate prescribing analysis: Insights from a single-centred retrospective study. BMJ Open.

[189] Imhann F., Bonder M.J., Vich Vila A., Fu J., Mujagic Z., Vork L., Tigchelaar E.F., Jankipersadsing S.A., Cenit M.C., Harmsen H.J. (2016). Proton pump inhibitors affect the gut microbiome. Gut.

[190] Bruno G., Zaccari P., Rocco G., Scalese G., Panetta C., Porowska B., Pontone S., Severi C. (2019). Proton pump inhibitors and dysbiosis: Current knowledge and aspects to be clarified. World J. Gastroenterol..

[191] Sjöstedt P., Enander J., Isung J. (2021). Serotonin Reuptake Inhibitors and the Gut Microbiome: Significance of the Gut Microbiome in Relation to Mechanism of Action, Treatment Response, Side Effects, and Tachyphylaxis. Front. Psychiatry.

[192] Letchumanan V., Thye A.Y.-K., Tan L.T.-H., Law J.W.-F., Johnson D., Ser H.-L., Bhuvanendran S., Thurairajasingam S., Lee L.-H. (2021). IDDF2021-ABS-0164 Gut feelings in depression: Microbiota dysbiosis in response to antidepressants. Gut.

[193] Morgan A.P., Crowley J.J., Nonneman R.J., Quackenbush C.R., Miller C.N., Ryan A.K., Bogue M.A., Paredes S.H., Yourstone S., Carroll I.M. (2014). The antipsychotic olanzapine interacts with the gut microbiome to cause weight gain in mouse. PLoS ONE.

[194] Melis M., Vascellari S., Santoru M.L., Oppo V., Fabbri M., Sarchioto M., Murgia D., Zibetti M., Lopiano L., Serra A. (2021). Gut microbiota and metabolome distinctive features in Parkinson disease: Focus on levodopa and levodopa-carbidopa intrajejunal gel. Eur. J. Neurol..

[195] Lubomski M., Tan A.H., Lim S.-Y., Holmes A.J., Davis R.L., Sue C.M. (2020). Parkinson’s disease and the gastrointestinal microbiome. J. Neurol..

[196] Palacios N., Hannoun A., Flahive J., Ward D., Goostrey K., Deb A., Smith K.M. (2021). Effect of Levodopa Initiation on the Gut Microbiota in Parkinson’s Disease. Front. Neurol..

[197] Maini Rekdal V., Bess E.N., Bisanz J.E., Turnbaugh P.J., Balskus E.P. (2019). Discovery and inhibition of an interspecies gut bacterial pathway for Levodopa metabolism. Science.

[198] Barichella M., Severgnini M., Cilia R., Cassani E., Bolliri C., Caronni S., Ferri V., Cancello R., Ceccarani C., Faierman S. (2019). Unraveling gut microbiota in Parkinson’s disease and atypical parkinsonism. Mov. Disord..

[199] Hill-Burns E.M., Debelius J.W., Morton J.T., Wissemann W.T., Lewis M.R., Wallen Z.D., Peddada S.D., Factor S.A., Molho E., Zabetian C.P. (2017). Parkinson’s disease and Parkinson’s disease medications have distinct signatures of the gut microbiome. Mov. Disord..

[200] Scheperjans F., Aho V., Pereira P.A., Koskinen K., Paulin L., Pekkonen E., Haapaniemi E., Kaakkola S., Eerola-Rautio J., Pohja M. (2015). Gut microbiota are related to Parkinson’s disease and clinical phenotype. Mov. Disord..

[201] Nguyen V.T.T., Sallbach J., Dos Santos Guilherme M., Endres K. (2021). Influence of Acetylcholine Esterase Inhibitors and Memantine, Clinically Approved for Alzheimer’s Dementia Treatment, on Intestinal Properties of the Mouse. Int. J. Mol. Sci..

[202] Spencer C.M., Noble S. (1998). Rivastigmine. A review of its use in Alzheimer’s disease. Drugs Aging.

[203] Pagliuca R., Papa M.V., Ilaria P.M., Papa V.F., Varricchio G. (2023). Atypical Presentation of Acetylcholinesterase Inhibitor-Induced Diarrhea in Older Adults with Cognitive Decline: An Aspect not to be Underestimated. Ann. Geriatr. Med. Res..

[204] Bahr S.M., Weidemann B.J., Castro A.N., Walsh J.W., deLeon O., Burnett C.M.L., Pearson N.A., Murry D.J., Grobe J.L., Kirby J.R. (2015). Risperidone-induced weight gain is mediated through shifts in the gut microbiome and suppression of energy expenditure. eBioMedicine.

[205] Cussotto S., Clarke G., Dinan T.G., Cryan J.F. (2019). Psychotropics and the Microbiome: A Chamber of Secrets…. Psychopharmacology.

[206] Boertien J.M., Pereira P.A., Aho V.T., Scheperjans F. (2019). Increasing comparability and utility of gut microbiome studies in Parkinson’s disease: A systematic review. J. Park. Dis..

[207] Haikal C., Chen Q.-Q., Li J.-Y. (2019). Microbiome changes: An indicator of Parkinson’s disease?. Transl. Neurodegener..

[208] Heinzel S., Aho V.T., Suenkel U., von Thaler A.K., Schulte C., Deuschle C., Paulin L., Hantunen S., Brockmann K., Eschweiler G.W. (2020). Gut microbiome signatures of risk and prodromal markers of Parkinson disease. Ann. Neurol..

[209] Nishiwaki H., Ito M., Ishida T., Hamaguchi T., Maeda T., Kashihara K., Tsuboi Y., Ueyama J., Shimamura T., Mori H. (2020). Meta-Analysis of Gut Dysbiosis in Parkinson’s Disease. Mov. Disord..

[210] Bullich C., Keshavarzian A., Garssen J., Kraneveld A., Perez-Pardo P. (2019). Gut Vibes in Parkinson’s Disease: The Microbiota-Gut-Brain Axis. Mov. Disord. Clin. Pract..

[211] Sampson T.R., Debelius J.W., Thron T., Janssen S., Shastri G.G., Ilhan Z.E., Challis C., Schretter C.E., Rocha S., Gradinaru V. (2016). Gut Microbiota Regulate Motor Deficits and Neuroinflammation in a Model of Parkinson’s Disease. Cell.

[212] Grabrucker S., Marizzoni M., Silajdžić E., Lopizzo N., Mombelli E., Nicolas S., Dohm-Hansen S., Scassellati C., Moretti D.V., Rosa M. (2022). Faecal microbiota transplantation from Alzheimer’s participants induces impairments in neurogenesis and cognitive behaviours in rats. bioRxiv.

[213] Keshavarzian A., Green S.J., Engen P.A., Voigt R.M., Naqib A., Forsyth C.B., Mutlu E., Shannon K.M. (2015). Colonic bacterial composition in Parkinson’s disease. Mov. Disord..

[214] Bedarf J.R., Hildebrand F., Coelho L.P., Sunagawa S., Bahram M., Goeser F., Bork P., Wüllner U. (2017). Functional implications of microbial and viral gut metagenome changes in early stage L-DOPA-naïve Parkinson’s disease patients. Genome Med..

[215] Hasegawa S., Goto S., Tsuji H., Okuno T., Asahara T., Nomoto K., Shibata A., Fujisawa Y., Minato T., Okamoto A. (2015). Intestinal dysbiosis and lowered serum lipopolysaccharide-binding protein in Parkinson’s disease. PLoS ONE.

[216] Hopfner F., Künstner A., Müller S.H., Künzel S., Zeuner K.E., Margraf N.G., Deuschl G., Baines J.F., Kuhlenbäumer G. (2017). Gut microbiota in Parkinson disease in a northern German cohort. Brain Res..

[217] Li C., Cui L., Yang Y., Miao J., Zhao X., Zhang J., Cui G., Zhang Y. (2019). Gut microbiota differs between Parkinson’s disease patients and healthy controls in northeast China. Front. Mol. Neurosci..

[218] Li F., Wang P., Chen Z., Sui X., Xie X., Zhang J. (2019). Alteration of the fecal microbiota in North-Eastern Han Chinese population with sporadic Parkinson’s disease. Neurosci. Lett..

[219] Li W., Wu X., Hu X., Wang T., Liang S., Duan Y., Jin F., Qin B. (2017). Structural changes of gut microbiota in Parkinson’s disease and its correlation with clinical features. Sci. China Life Sci..

[220] Lin A., Zheng W., He Y., Tang W., Wei X., He R., Huang W., Su Y., Huang Y., Zhou H. (2018). Gut microbiota in patients with Parkinson’s disease in southern China. Park. Relat. Disord..

[221] Qian Y., Yang X., Xu S., Wu C., Song Y., Qin N., Chen S.-D., Xiao Q. (2018). Alteration of the fecal microbiota in Chinese patients with Parkinson’s disease. Brain Behav. Immun..

[222] Unger M.M., Spiegel J., Dillmann K.-U., Grundmann D., Philippeit H., Bürmann J., Faßbender K., Schwiertz A., Schäfer K.-H. (2016). Short chain fatty acids and gut microbiota differ between patients with Parkinson’s disease and age-matched controls. Park. Relat. Disord..

[223] Vascellari S., Palmas V., Melis M., Pisanu S., Cusano R., Uva P., Perra D., Madau V., Sarchioto M., Oppo V. (2020). Gut microbiota and metabolome alterations associated with Parkinson’s disease. Msystems.

[224] Baldini F., Hertel J., Sandt E., Thinnes C.C., Neuberger-Castillo L., Pavelka L., Betsou F., Krüger R., Thiele I. (2020). Parkinson’s disease-associated alterations of the gut microbiome predict disease-relevant changes in metabolic functions. BMC Biol..

[225] Cirstea M.S., Yu A.C., Golz E., Sundvick K., Kliger D., Radisavljevic N., Foulger L.H., Mackenzie M., Huan T., Finlay B.B. (2020). Microbiota Composition and Metabolism Are Associated With Gut Function in Parkinson’s Disease. Mov. Disord..

[226] Heintz-Buschart A., Pandey U., Wicke T., Sixel-Döring F., Janzen A., Sittig-Wiegand E., Trenkwalder C., Oertel W.H., Mollenhauer B., Wilmes P. (2018). The nasal and gut microbiome in Parkinson’s disease and idiopathic rapid eye movement sleep behavior disorder. Mov. Disord..

[227] Jin M., Li J., Liu F., Lyu N., Wang K., Wang L., Liang S., Tao H., Zhu B., Alkasir R. (2019). Analysis of the Gut Microflora in Patients With Parkinson’s Disease. Front. Neurosci..

[228] Wallen Z.D., Appah M., Dean M.N., Sesler C.L., Factor S.A., Molho E., Zabetian C.P., Standaert D.G., Payami H. (2020). Characterizing dysbiosis of gut microbiome in PD: Evidence for overabundance of opportunistic pathogens. npj Park. Dis..

[229] Forsyth C.B., Shannon K.M., Kordower J.H., Voigt R.M., Shaikh M., Jaglin J.A., Estes J.D., Dodiya H.B., Keshavarzian A. (2011). Increased intestinal permeability correlates with sigmoid mucosa alpha-synuclein staining and endotoxin exposure markers in early Parkinson’s disease. PLoS ONE.

[230] Toledo A.R.L., Monroy G.R., Salazar F.E., Lee J.-Y., Jain S., Yadav H., Borlongan C.V. (2022). Gut–brain axis as a pathological and therapeutic target for neurodegenerative disorders. Int. J. Mol. Sci..

[231] Sarkar S.R., Banerjee S. (2019). Gut microbiota in neurodegenerative disorders. J. Neuroimmunol..

[232] Fitzgerald E., Murphy S., Martinson H.A. (2019). Alpha-Synuclein Pathology and the Role of the Microbiota in Parkinson’s Disease. Front. Neurosci..

[233] Friedland R.P., Chapman M.R. (2017). The role of microbial amyloid in neurodegeneration. PLoS Pathog..

[234] Kim S., Kwon S.-H., Kam T.-I., Panicker N., Karuppagounder S.S., Lee S., Lee J.H., Kim W.R., Kook M., Foss C.A. (2019). Transneuronal Propagation of Pathologic α-Synuclein from the Gut to the Brain Models Parkinson’s Disease. Neuron.

[235] Uemura N., Yagi H., Uemura M.T., Hatanaka Y., Yamakado H., Takahashi R. (2018). Inoculation of α-synuclein preformed fibrils into the mouse gastrointestinal tract induces Lewy body-like aggregates in the brainstem via the vagus nerve. Mol. Neurodegener..

[236] Liu B., Fang F., Pedersen N.L., Tillander A., Ludvigsson J.F., Ekbom A., Svenningsson P., Chen H., Wirdefeldt K. (2017). Vagotomy and Parkinson disease: A Swedish register–based matched-cohort study. Neurology.

[237] Kouli A., Horne C.B., Williams-Gray C.H. (2019). Toll-like receptors and their therapeutic potential in Parkinson’s disease and α-synucleinopathies. Brain Behav. Immun..

[238] Van Den Berge N., Ferreira N., Mikkelsen T.W., Alstrup A.K.O., Tamgüney G., Karlsson P., Terkelsen A.J., Nyengaard J.R., Jensen P.H., Borghammer P. (2021). Ageing promotes pathological alpha-synuclein propagation and autonomic dysfunction in wild-type rats. Brain.

[239] Zhou Y., Blanco L.P., Smith D.R., Chapman M.R. (2012). Bacterial amyloids. Methods Mol. Biol..

[240] Lesnick T.G., Papapetropoulos S., Mash D.C., Ffrench-Mullen J., Shehadeh L., De Andrade M., Henley J.R., Rocca W.A., Ahlskog J.E., Maraganore D.M. (2007). A genomic pathway approach to a complex disease: Axon guidance and Parkinson disease. PLoS Genet..

[241] Nalls M.A., Blauwendraat C., Vallerga C.L., Heilbron K., Bandres-Ciga S., Chang D., Tan M., Kia D.A., Noyce A.J., Xue A. (2019). Identification of novel risk loci, causal insights, and heritable risk for Parkinson’s disease: A meta-analysis of genome-wide association studies. Lancet Neurol..

[242] Pang S.Y.-Y., Ho P.W.-L., Liu H.-F., Leung C.-T., Li L., Chang E.E.S., Ramsden D.B., Ho S.-L. (2019). The interplay of aging, genetics and environmental factors in the pathogenesis of Parkinson’s disease. Transl. Neurodegener..

[243] Marras C., Canning C.G., Goldman S.M. (2019). Environment, lifestyle, and Parkinson’s disease: Implications for prevention in the next decade. Mov. Disord..

[244] Cheng H.C., Ulane C.M., Burke R.E. (2010). Clinical progression in Parkinson disease and the neurobiology of axons. Ann. Neurol..

[245] Kaye J., Gage H., Kimber A., Storey L., Trend P. (2006). Excess burden of constipation in Parkinson’s disease: A pilot study. Mov. Disord. Off. J. Mov. Disord. Soc..

[246] Knudsen K., Krogh K., Østergaard K., Borghammer P. (2017). Constipation in Parkinson’s disease: Subjective symptoms, objective markers, and new perspectives. Mov. Disord..

[247] Berg D., Postuma R.B., Adler C.H., Bloem B.R., Chan P., Dubois B., Gasser T., Goetz C.G., Halliday G., Joseph L. (2015). MDS research criteria for prodromal Parkinson’s disease. Mov. Disord..

[248] Shannon K.M., Keshavarzian A., Dodiya H.B., Jakate S., Kordower J.H. (2012). Is alpha-synuclein in the colon a biomarker for premotor Parkinson’s disease? Evidence from 3 cases. Mov. Disord..

[249] Braak H., Rüb U., Gai W., Del Tredici K. (2003). Idiopathic Parkinson’s disease: Possible routes by which vulnerable neuronal types may be subject to neuroinvasion by an unknown pathogen. J. Neural Transm..

[250] Hawkes C.H., Del Tredici K., Braak H. (2007). Parkinson’s disease: A dual-hit hypothesis. Neuropathol. Appl. Neurobiol..

[251] Barbut D., Stolzenberg E., Zasloff M. (2019). Gastrointestinal Immunity and Alpha-Synuclein. J. Park. Dis..

[252] Chen S.G., Stribinskis V., Rane M.J., Demuth D.R., Gozal E., Roberts A.M., Jagadapillai R., Liu R., Choe K., Shivakumar B. (2016). Exposure to the functional bacterial amyloid protein curli enhances alpha-synuclein aggregation in aged Fischer 344 rats and Caenorhabditis elegans. Sci. Rep..

[253] Vitetta L., Chen J., Clarke S. (2019). The vermiform appendix: An immunological organ sustaining a microbiome inoculum. Clin. Sci..

[254] Killinger B., Labrie V. (2019). The Appendix in Parkinson’s Disease: From Vestigial Remnant to Vital Organ?. J. Park. Dis..

[255] Villumsen M., Aznar S., Pakkenberg B., Jess T., Brudek T. (2019). Inflammatory bowel disease increases the risk of Parkinson’s disease: A Danish nationwide cohort study 1977–2014. Gut.

[256] Fu P., Gao M., Yung K.K.L. (2019). Association of intestinal disorders with Parkinson’s disease and Alzheimer’s disease: A systematic review and meta-analysis. ACS Chem. Neurosci..

[257] Liu B., Sjölander A., Pedersen N.L., Ludvigsson J.F., Chen H., Fang F., Wirdefeldt K. (2021). Irritable bowel syndrome and Parkinson’s disease risk: Register-based studies. npj Park. Dis..

[258] Ternák G., Kuti D., Kovács K.J. (2020). Dysbiosis in Parkinson’s disease might be triggered by certain antibiotics. Med. Hypotheses.

[259] Mertsalmi T.H., Pekkonen E., Scheperjans F. (2020). Antibiotic exposure and risk of Parkinson’s disease in Finland: A nationwide case-control study. Mov. Disord..

[260] Aho V.T.E., Houser M.C., Pereira P.A.B., Chang J., Rudi K., Paulin L., Hertzberg V., Auvinen P., Tansey M.G., Scheperjans F. (2021). Relationships of gut microbiota, short-chain fatty acids, inflammation, and the gut barrier in Parkinson’s disease. Mol. Neurodegener..

[261] Lin C.-H., Chen C.-C., Chiang H.-L., Liou J.-M., Chang C.-M., Lu T.-P., Chuang E.Y., Tai Y.-C., Cheng C., Lin H.-Y. (2019). Altered gut microbiota and inflammatory cytokine responses in patients with Parkinson’s disease. J. Neuroinflammation.

[262] Wallen Z.D., Demirkan A., Twa G., Cohen G., Dean M.N., Standaert D.G., Sampson T.R., Payami H. (2022). Metagenomics of Parkinson’s disease implicates the gut microbiome in multiple disease mechanisms. Nat. Commun..

[263] Toh T.S., Chong C.W., Lim S.-Y., Bowman J., Cirstea M., Lin C.-H., Chen C.-C., Appel-Cresswell S., Finlay B.B., Tan A.H. (2022). Gut microbiome in Parkinson’s disease: New insights from meta-analysis. Park. Relat. Disord..

[264] Romano S., Savva G.M., Bedarf J.R., Charles I.G., Hildebrand F., Narbad A. (2021). Meta-analysis of the Parkinson’s disease gut microbiome suggests alterations linked to intestinal inflammation. npj Park. Dis..

[265] Boulos C., Yaghi N., El Hayeck R., Heraoui G.N., Fakhoury-Sayegh N. (2019). Nutritional risk factors, microbiota and Parkinson’s disease: What is the current evidence?. Nutrients.

[266] Knight E., Geetha T., Burnett D., Babu J.R. (2022). The Role of Diet and Dietary Patterns in Parkinson’s Disease. Nutrients.

[267] Zhang C.-Y., He F.-F., Su H., Zhang C., Meng X.-F. (2020). Association between chronic kidney disease and Alzheimer’s disease: An update. Metab. Brain Dis..

[268] Bian J., Liebert A., Bicknell B., Chen X.-M., Huang C., Pollock C.A. (2022). Faecal Microbiota Transplantation and Chronic Kidney Disease. Nutrients.

[269] Hill J.M., Clement C., Pogue A.I., Bhattacharjee S., Zhao Y., Lukiw W.J. (2014). Pathogenic microbes, the microbiome, and Alzheimer’s disease (AD). Front. Aging Neurosci..

[270] Hersant H., Grossberg G. (2022). The Ketogenic Diet and Alzheimer’s Disease. J. Nutr. Health Aging.

[271] Grant W.B. (2016). Using multicountry ecological and observational studies to determine dietary risk factors for Alzheimer’s disease. J. Am. Coll. Nutr..

[272] Chandra S., Sisodia S.S., Vassar R.J. (2023). The gut microbiome in Alzheimer’s disease: What we know and what remains to be explored. Mol. Neurodegener..

[273] Kim M., Park S.J., Choi S., Chang J., Kim S.M., Jeong S., Park Y.J., Lee G., Son J.S., Ahn J.C. (2022). Association between antibiotics and dementia risk: A retrospective cohort study. Front. Pharmacol..

[274] Vogt N.M., Kerby R.L., Dill-McFarland K.A., Harding S.J., Merluzzi A.P., Johnson S.C., Carlsson C.M., Asthana S., Zetterberg H., Blennow K. (2017). Gut microbiome alterations in Alzheimer’s disease. Sci. Rep..

[275] Liu P., Wu L., Peng G., Han Y., Tang R., Ge J., Zhang L., Jia L., Yue S., Zhou K. (2019). Altered microbiomes distinguish Alzheimer’s disease from amnestic mild cognitive impairment and health in a Chinese cohort. Brain Behav. Immun..

[276] Zhuang Z.-Q., Shen L.-L., Li W.-W., Fu X., Zeng F., Gui L., Lü Y., Cai M., Zhu C., Tan Y.-L. (2018). Gut microbiota is altered in patients with Alzheimer’s disease. J. Alzheimer’s Dis..

[277] Asti A., Gioglio L. (2014). Can a bacterial endotoxin be a key factor in the kinetics of amyloid fibril formation?. J. Alzheimer’s Dis..

[278] Almeida M.N., Silvernale C., Kuo B., Staller K. (2019). Bowel symptoms predate the diagnosis among many patients with multiple sclerosis: A 14-year cohort study. Neurogastroenterol. Motil..

[279] Khanna L., Zeydan B., Kantarci O.H., Camilleri M. (2022). Gastrointestinal motility disorders in patients with multiple sclerosis: A single-center study. Neurogastroenterol. Motil..

[280] Cekanaviciute E., Yoo B.B., Runia T.F., Debelius J.W., Singh S., Nelson C.A., Kanner R., Bencosme Y., Lee Y.K., Hauser S.L. (2017). Gut bacteria from multiple sclerosis patients modulate human T cells and exacerbate symptoms in mouse models. Proc. Natl. Acad. Sci. USA.

[281] Cox L.M., Maghzi A.H., Liu S., Tankou S.K., Dhang F.H., Willocq V., Song A., Wasén C., Tauhid S., Chu R. (2021). Gut Microbiome in Progressive Multiple Sclerosis. Ann. Neurol..

[282] Jangi S., Gandhi R., Cox L.M., Li N., von Glehn F., Yan R., Patel B., Mazzola M.A., Liu S., Glanz B.L. (2016). Alterations of the human gut microbiome in multiple sclerosis. Nat. Commun..

[283] Cosorich I., Dalla-Costa G., Sorini C., Ferrarese R., Messina M.J., Dolpady J., Radice E., Mariani A., Testoni P.A., Canducci F. (2017). High frequency of intestinal T_H_17 cells correlates with microbiota alterations and disease activity in multiple sclerosis. Sci. Adv..

[284] Miyake S., Kim S., Suda W., Oshima K., Nakamura M., Matsuoka T., Chihara N., Tomita A., Sato W., Kim S.-W. (2015). Dysbiosis in the Gut Microbiota of Patients with Multiple Sclerosis, with a Striking Depletion of Species Belonging to Clostridia XIVa and IV Clusters. PLoS ONE.

[285] Chen J., Chia N., Kalari K.R., Yao J.Z., Novotna M., Paz Soldan M.M., Luckey D.H., Marietta E.V., Jeraldo P.R., Chen X. (2016). Multiple sclerosis patients have a distinct gut microbiota compared to healthy controls. Sci. Rep..

[286] Reynders T., Devolder L., Valles-Colomer M., Van Remoortel A., Joossens M., De Keyser J., Nagels G., D’hooghe M., Raes J. (2020). Gut microbiome variation is associated to Multiple Sclerosis phenotypic subtypes. Ann. Clin. Transl. Neurol..

[287] Melbye P., Olsson A., Hansen T.H., Søndergaard H.B., Bang Oturai A. (2019). Short-chain fatty acids and gut microbiota in multiple sclerosis. Acta Neurol. Scand..

[288] Sun J., Zhan Y., Mariosa D., Larsson H., Almqvist C., Ingre C., Zagai U., Pawitan Y., Fang F. (2019). Antibiotics use and risk of amyotrophic lateral sclerosis in Sweden. Eur. J. Neurol..

[289] Fang X., Wang X., Yang S., Meng F., Wang X., Wei H., Chen T. (2016). Evaluation of the Microbial Diversity in Amyotrophic Lateral Sclerosis Using High-Throughput Sequencing. Front. Microbiol..

[290] Di Gioia D., Bozzi Cionci N., Baffoni L., Amoruso A., Pane M., Mogna L., Gaggìa F., Lucenti M.A., Bersano E., Cantello R. (2020). A prospective longitudinal study on themicrobiota composition in amyotrophic lateral sclerosis. BMC Med..

[291] Ngo S.T., Restuadi R., McCrae A.F., Van Eijk R.P., Garton F., Henderson R.D., Wray N.R., McCombe P.A., Steyn F.J. (2020). Progression and survival of patients with motor neuron disease relative to their fecal microbiota. Amyotroph. Lateral Scler. Front. Degener..

[292] Boddy S.L., Giovannelli I., Sassani M., Cooper-Knock J., Snyder M.P., Segal E., Elinav E., Barker L.A., Shaw P.J., McDermott C.J. (2021). The gut microbiome: A key player in the complexity of amyotrophic lateral sclerosis (ALS). BMC Med..

[293] Woerman A.L., Watts J.C., Aoyagi A., Giles K., Middleton L.T., Prusiner S.B. (2018). α-Synuclein: Multiple System Atrophy Prions. Cold Spring Harb. Perspect. Med..

[294] Mishima T., Fujioka S., Kawazoe M., Inoue K., Arima H., Tsuboi Y. (2022). Constipation Symptoms in Multiple System Atrophy Using Rome Criteria and Their Impact on Personalized Medicine. J. Pers Med..

[295] Engen P.A., Dodiya H.B., Naqib A., Forsyth C.B., Green S.J., Voigt R.M., Kordower J.H., Mutlu E.A., Shannon K.M., Keshavarzian A. (2017). The Potential Role of Gut-Derived Inflammation in Multiple System Atrophy. J. Park. Dis..

[296] Wan L., Zhou X., Wang C., Chen Z., Peng H., Hou X., Peng Y., Wang P., Li T., Yuan H. (2019). Alterations of the Gut Microbiota in Multiple System Atrophy Patients. Front. Neurosci..

[297] Gerhardt S., Mohajeri M.H. (2018). Changes of Colonic Bacterial Composition in Parkinson’s Disease and Other Neurodegenerative Diseases. Nutrients.

[298] Kong G., Lê Cao K.-A., Judd L.M., Li S., Renoir T., Hannan A.J. (2020). Microbiome profiling reveals gut dysbiosis in a transgenic mouse model of Huntington’s disease. Neurobiol. Dis..

[299] Guo Y., Xu Y., Lin X., Zhen Z., Yi F., Guan H., Shi Q., Sun W., Yang A., Dong X. (2022). Creutzfeldt-Jakob Disease: Alterations of Gut Microbiota. Front. Neurol..

[300] Gondalia S.V., Palombo E.A., Knowles S.R., Cox S.B., Meyer D., Austin D.W. (2012). Molecular characterisation of gastrointestinal microbiota of children with autism (with and without gastrointestinal dysfunction) and their neurotypical siblings. Autism Res..

[301] Kang D.-W., Adams J.B., Gregory A.C., Borody T., Chittick L., Fasano A., Khoruts A., Geis E., Maldonado J., McDonough-Means S. (2017). Microbiota Transfer Therapy alters gut ecosystem and improves gastrointestinal and autism symptoms: An open-label study. Microbiome.

[302] West K.A., Yin X., Rutherford E.M., Wee B., Choi J., Chrisman B.S., Dunlap K.L., Hannibal R.L., Hartono W., Lin M. (2022). Multi-angle meta-analysis of the gut microbiome in Autism Spectrum Disorder: A step toward understanding patient subgroups. Sci. Rep..

[303] Bandini L.G., Curtin C., Phillips S., Anderson S.E., Maslin M., Must A. (2017). Changes in Food Selectivity in Children with Autism Spectrum Disorder. J. Autism Dev. Disord..

[304] Sharon G., Cruz N.J., Kang D.-W., Gandal M.J., Wang B., Kim Y.-M., Zink E.M., Casey C.P., Taylor B.C., Lane C.J. (2019). Human gut microbiota from autism spectrum disorder promote behavioral symptoms in mice. Cell.

[305] Davies C., Mishra D., Eshraghi R.S., Mittal J., Sinha R., Bulut E., Mittal R., Eshraghi A.A. (2021). Altering the gut microbiome to potentially modulate behavioral manifestations in autism spectrum disorders: A systematic review. Neurosci. Biobehav. Rev..

[306] Li N., Yang J., Zhang J., Liang C., Wang Y., Chen B., Zhao C., Wang J., Zhang G., Zhao D. (2019). Correlation of Gut Microbiome Between ASD Children and Mothers and Potential Biomarkers for Risk Assessment. Genom. Proteom. Bioinform..

[307] Fowlie G., Cohen N., Ming X. (2018). The Perturbance of Microbiome and Gut-Brain Axis in Autism Spectrum Disorders. Int. J. Mol. Sci..

[308] Liu S., Rezende R.M., Moreira T.G., Tankou S.K., Cox L.M., Wu M., Song A., Dhang F.H., Wei Z., Costamagna G. (2019). Oral administration of miR-30d from feces of MS patients suppresses MS-like symptoms in mice by expanding Akkermansia muciniphila. Cell Host Microbe.

[309] Bolte E.R. (1998). Autism and Clostridium tetani. Med. Hypotheses.

[310] Zeidán-Chuliá F., Fonseca Moreira J. (2013). Clostridium Bacteria and its Impact in Autism Research: Thinking “Outside The Box” of Neuroscience. Med. Hypotheses.

[311] Alshammari M.K., AlKhulaifi M.M., Al Farraj D.A., Somily A.M., Albarrag A.M. (2020). Incidence of Clostridium perfringens and its toxin genes in the gut of children with autism spectrum disorder. Anaerobe.

[312] Wang L., Yu Y.-M., Zhang Y.-q., Zhang J., Lu N., Liu N. (2018). Hydrogen breath test to detect small intestinal bacterial overgrowth: A prevalence case–control study in autism. Eur. Child Adolesc. Psychiatry.

[313] Checa-Ros A., Jeréz-Calero A., Molina-Carballo A., Campoy C., Muñoz-Hoyos A. (2021). Current Evidence on the Role of the Gut Microbiome in ADHD Pathophysiology and Therapeutic Implications. Nutrients.

[314] Sukmajaya A.C., Lusida M.I., Soetjipto, Setiawati Y. (2021). Systematic review of gut microbiota and attention-deficit hyperactivity disorder (ADHD). Ann. Gen. Psychiatry.

[315] Aarts E., Ederveen T.H., Naaijen J., Zwiers M.P., Boekhorst J., Timmerman H.M., Smeekens S.P., Netea M.G., Buitelaar J.K., Franke B. (2017). Gut microbiome in ADHD and its relation to neural reward anticipation. PLoS ONE.

[316] Jiang H.-y., Zhou Y.-y., Zhou G.-l., Li Y.-c., Yuan J., Li X.-h., Ruan B. (2018). Gut microbiota profiles in treatment-naïve children with attention deficit hyperactivity disorder. Behav. Brain Res..

[317] Szopinska-Tokov J., Dam S., Naaijen J., Konstanti P., Rommelse N., Belzer C., Buitelaar J., Franke B., Bloemendaal M., Aarts E. (2020). Investigating the gut microbiota composition of individuals with attention-deficit/hyperactivity disorder and association with symptoms. Microorganisms.

[318] Liu Q., Mak J.W.Y., Su Q., Yeoh Y.K., Lui G.C.-Y., Ng S.S.S., Zhang F., Li A.Y.L., Lu W., Hui D.S.-C. (2022). Gut microbiota dynamics in a prospective cohort of patients with post-acute COVID-19 syndrome. Gut.

[319] Vakili K., Fathi M., Yaghoobpoor S., Sayehmiri F., Nazerian Y., Nazerian A., Mohamadkhani A., Khodabakhsh P., Réus G.Z., Hajibeygi R. (2022). The contribution of gut-brain axis to development of neurological symptoms in COVID-19 recovered patients: A hypothesis and review of literature. Front. Cell. Infect. Microbiol..

[320] Graham E.L., Clark J.R., Orban Z.S., Lim P.H., Szymanski A.L., Taylor C., DiBiase R.M., Jia D.T., Balabanov R., Ho S.U. (2021). Persistent neurologic symptoms and cognitive dysfunction in non-hospitalized Covid-19 “long haulers”. Ann. Clin. Transl. Neurol..

[321] Asadi-Pooya A.A., Akbari A., Emami A., Lotfi M., Rostamihosseinkhani M., Nemati H., Barzegar Z., Kabiri M., Zeraatpisheh Z., Farjoud-Kouhanjani M. (2022). Long COVID syndrome-associated brain fog. J. Med. Virol..

[322] Chambers P. (2023). Long Covid and Neurodegenerative Disease. Preprints.

[323] Hilpert K., Mikut R. (2021). Is There a Connection Between Gut Microbiome Dysbiosis Occurring in COVID-19 Patients and Post-COVID-19 Symptoms?. Front. Microbiol..

[324] Yeoh Y.K., Zuo T., Lui G.C.-Y., Zhang F., Liu Q., Li A.Y., Chung A.C., Cheung C.P., Tso E.Y., Fung K.S. (2021). Gut microbiota composition reflects disease severity and dysfunctional immune responses in patients with COVID-19. Gut.

[325] Chen Y., Gu S., Chen Y., Lu H., Shi D., Guo J., Wu W.-R., Yang Y., Li Y., Xu K.-J. (2022). Six-month follow-up of gut microbiota richness in patients with COVID-19. Gut.

[326] Giloteaux L., Goodrich J.K., Walters W.A., Levine S.M., Ley R.E., Hanson M.R. (2016). Reduced diversity and altered composition of the gut microbiome in individuals with myalgic encephalomyelitis/chronic fatigue syndrome. Microbiome.

[327] König R.S., Albrich W.C., Kahlert C.R., Bahr L.S., Löber U., Vernazza P., Scheibenbogen C., Forslund S.K. (2022). The Gut Microbiome in Myalgic Encephalomyelitis (ME)/Chronic Fatigue Syndrome (CFS). Front. Immunol..

[328] Chu L., Valencia I.J., Garvert D.W., Montoya J.G. (2019). Onset patterns and course of myalgic encephalomyelitis/chronic fatigue syndrome. Front. Pediatr..

[329] Frémont M., Coomans D., Massart S., De Meirleir K. (2013). High-throughput 16S rRNA gene sequencing reveals alterations of intestinal microbiota in myalgic encephalomyelitis/chronic fatigue syndrome patients. Anaerobe.

[330] Newberry F., Hsieh S.-Y., Wileman T., Carding S.R. (2018). Does the microbiome and virome contribute to myalgic encephalomyelitis/chronic fatigue syndrome?. Clin. Sci..

[331] Robertson B. (2010). Introduction to the Journal of Physiology’s Special edition on neurological channelopathies. J. Physiol..

[332] Yue Q., Cai M., Xiao B., Zhan Q., Zeng C. (2022). The Microbiota-Gut-Brain Axis and Epilepsy. Cell Mol. Neurobiol..

[333] Amaral F., Sachs D., Costa V., Fagundes C., Cisalpino D., Cunha T., Ferreira S., Cunha F., Silva T., Nicoli J. (2008). Commensal microbiota is fundamental for the development of inflammatory pain. Proc. Natl. Acad. Sci. USA.

[334] Lin B., Wang Y., Zhang P., Yuan Y., Zhang Y., Chen G. (2020). Gut microbiota regulates neuropathic pain: Potential mechanisms and therapeutic strategy. J. Headache Pain.

[335] Guo R., Chen L.-H., Xing C., Liu T. (2019). Pain regulation by gut microbiota: Molecular mechanisms and therapeutic potential. Br. J. Anaesth..

[336] Belrose J.C., Noppens R.R. (2019). Anesthesiology and cognitive impairment: A narrative review of current clinical literature. BMC Anesthesiol..

[337] Sugita S., Tahir P., Kinjo S. (2023). The effects of microbiome-targeted therapy on cognitive impairment and postoperative cognitive dysfunction-A systematic review. PLoS ONE.

[338] Xu X., Hu Y., Yan E., Zhan G., Liu C., Yang C. (2020). Perioperative neurocognitive dysfunction: Thinking from the gut?. Aging.

[339] Zhan G., Hua D., Huang N., Wang Y., Li S., Zhou Z., Yang N., Jiang R., Zhu B., Yang L. (2019). Anesthesia and surgery induce cognitive dysfunction in elderly male mice: The role of gut microbiota. Aging.

[340] Dong L., Li J., Zhang C., Liu D.-X. (2021). Gut microbiota: A new player in the pathogenesis of perioperative neurocognitive disorder?. Ibrain.

[341] Liu H., Yin X., Li J., Cao Y., Wang Y., Mu W., Zhuo Z., Chen L., Zhang Z., Qu X. (2022). Preoperative intestinal microbiome and metabolome in elderly patients with delayed neurocognitive recovery. Anaesth. Crit. Care Pain Med..

[342] Lubomski M., Davis R.L., Sue C.M. (2019). The gut microbiota: A novel therapeutic target in Parkinson’s disease?. Park. Relat. Disord..

[343] DuPont H.L., Suescun J., Jiang Z.-D., Brown E.L., Iqbal T., Alexander A.S., DuPont A.W., Newark M., Essigmann H.T., Schiess M.C. (2020). Microbiome characterization and reversal of dysbiosis in Parkinson’s disease by Fecal Microbiota Transplantation (1825). Neurology.

[344] Xue L.J., Yang X.Z., Tong Q., Shen P., Ma S.J., Wu S.N., Zheng J.L., Wang H.G. (2020). Fecal microbiota transplantation therapy for Parkinson’s disease: A preliminary study. Medicine.

[345] Kraeuter A.-K., Phillips R., Sarnyai Z. (2020). Ketogenic therapy in neurodegenerative and psychiatric disorders: From mice to men. Prog. Neuro-Psychopharmacol. Biol. Psychiatry.

[346] Castelli V., d’Angelo M., Quintiliani M., Benedetti E., Cifone M.G., Cimini A. (2021). The emerging role of probiotics in neurodegenerative diseases: New hope for Parkinson’s disease?. Neural Regen Res..

[347] Bicknell B., Liebert A., Johnstone D., Kiat H. (2018). Photobiomodulation of the microbiome: Implications for metabolic and inflammatory diseases. Lasers Med. Sci..

[348] van den Brink A.C., Brouwer-Brolsma E.M., Berendsen A.A.M., van de Rest O. (2019). The Mediterranean, Dietary Approaches to Stop Hypertension (DASH), and Mediterranean-DASH Intervention for Neurodegenerative Delay (MIND) Diets Are Associated with Less Cognitive Decline and a Lower Risk of Alzheimer’s Disease—A Review. Adv. Nutr..

[349] Kheirouri S., Alizadeh M. (2022). MIND diet and cognitive performance in older adults: A systematic review. Crit. Rev. Food Sci. Nutr..

[350] de Crom T.O.E., Mooldijk S.S., Ikram M.K., Ikram M.A., Voortman T. (2022). MIND diet and the risk of dementia: A population-based study. Alzheimer’s Res. Ther..

[351] Agarwal P., Leurgans S.E., Agrawal S., Aggarwal N., Cherian I.J., James B.D., Dhana K., Barnes L.L., Bennett D.A., Schneider J.A. (2023). Association of Mediterranean-DASH Intervention for Neurodegenerative Delay and Mediterranean Diets With Alzheimer Disease Pathology. Neurology.

[352] Singh P., Tuck C., Gibson P.R., Chey W.D. (2022). The Role of Food in the Treatment of Bowel Disorders: Focus on Irritable Bowel Syndrome and Functional Constipation. Am. J. Gastroenterol..

[353] Chicco F., Magrì S., Cingolani A., Paduano D., Pesenti M., Zara F., Tumbarello F., Urru E., Melis A., Casula L. (2020). Multidimensional Impact of Mediterranean Diet on IBD Patients. Inflamm. Bowel Dis..

[354] Picchianti Diamanti A., Panebianco C., Salerno G., Di Rosa R., Salemi S., Sorgi M.L., Meneguzzi G., Mariani M.B., Rai A., Iacono D. (2020). Impact of Mediterranean diet on disease activity and gut microbiota composition of rheumatoid arthritis patients. Microorganisms.

[355] Maggi S., Ticinesi A., Limongi F., Noale M., Ecarnot F. (2023). The role of nutrition and the Mediterranean diet on the trajectories of cognitive decline. Exp. Gerontol..

[356] Wade A.T., Elias M.F., Murphy K.J. (2021). Adherence to a Mediterranean diet is associated with cognitive function in an older non-Mediterranean sample: Findings from the Maine-Syracuse Longitudinal Study. Nutr. Neurosci..

[357] Hegelmaier T., Lebbing M., Duscha A., Tomaske L., Tönges L., Holm J.B., Bjørn Nielsen H., Gatermann S.G., Przuntek H., Haghikia A. (2020). Interventional Influence of the Intestinal Microbiome Through Dietary Intervention and Bowel Cleansing Might Improve Motor Symptoms in Parkinson’s Disease. Cells.

[358] Dhana K., James B.D., Agarwal P., Aggarwal N.T., Cherian L.J., Leurgans S.E., Barnes L.L., Bennett D.A., Schneider J.A. (2021). MIND Diet, Common Brain Pathologies, and Cognition in Community-Dwelling Older Adults. J. Alzheimer’s Dis..

[359] Phillips M.C.L., Deprez L.M., Mortimer G.M.N., Murtagh D.K.J., McCoy S., Mylchreest R., Gilbertson L.J., Clark K.M., Simpson P.V., McManus E.J. (2021). Randomized crossover trial of a modified ketogenic diet in Alzheimer’s disease. Alzheimer’s Res. Ther..

[360] Zhang F., Fan D., Huang J.-l., Zuo T. (2022). The gut microbiome: Linking dietary fiber to inflammatory diseases. Med. Microecol..

[361] Whelan K., Staudacher H.M. (2022). Fibre is good for the microbiome: But what is the evidence?. Lancet Gastroenterol. Hepatol..

[362] Buffington S.A., Di Prisco G.V., Auchtung T.A., Ajami N.J., Petrosino J.F., Costa-Mattioli M. (2016). Microbial Reconstitution Reverses Maternal Diet-Induced Social and Synaptic Deficits in Offspring. Cell.

[363] Hoffman J.D., Yanckello L.M., Chlipala G., Hammond T.C., McCulloch S.D., Parikh I., Sun S., Morganti J.M., Green S.J., Lin A.L. (2019). Dietary inulin alters the gut microbiome, enhances systemic metabolism and reduces neuroinflammation in an APOE4 mouse model. PLoS ONE.

[364] Pérez-Monter C., Álvarez-Arce A., Nuño-Lambarri N., Escalona-Nández I., Juárez-Hernández E., Chávez-Tapia N.C., Uribe M., Barbero-Becerra V.J. (2022). Inulin Improves Diet-Induced Hepatic Steatosis and Increases Intestinal Akkermansia Genus Level. Int. J. Mol. Sci..

[365] Jalanka J., Major G., Murray K., Singh G., Nowak A., Kurtz C., Silos-Santiago I., Johnston J.M., de Vos W.M., Spiller R. (2019). The Effect of Psyllium Husk on Intestinal Microbiota in Constipated Patients and Healthy Controls. Int. J. Mol. Sci..

[366] Bretin A., Zou J., San Yeoh B., Ngo V.L., Winer S., Winer D.A., Reddivari L., Pellizzon M., Walters W.A., Patterson A.D. (2023). Psyllium fiber protects against colitis via activation of bile acid sensor fxr. Cell. Mol. Gastroenterol. Hepatol..

[367] Mostafa M.Y.A. (2017). Effect of Feeding Psyllium Husk (*Plantago Ovata*) Herbs on Rats suffering from Hyperglycemia. J. Specif. Educ. Stud. Res..

[368] Le Bastard Q., Chapelet G., Javaudin F., Lepelletier D., Batard E., Montassier E. (2020). The effects of inulin on gut microbial composition: A systematic review of evidence from human studies. Eur. J. Clin. Microbiol. Infect. Dis..

[369] Alipour Nosrani E., Tamtaji O.R., Alibolandi Z., Sarkar P., Ghazanfari M., Azami Tameh A., Taghizadeh M., Banikazemi Z., Hadavi R., Naderi Taheri M. (2021). Neuroprotective effects of probiotics bacteria on animal model of Parkinson’s disease induced by 6-hydroxydopamine: A behavioral, biochemical, and histological study. J. Immunoass. Immunochem..

[370] Ji H.-F., Shen L. (2021). Probiotics as potential therapeutic options for Alzheimer’s disease. Appl. Microbiol. Biotechnol..

[371] Mirzaei H., Sedighi S., Kouchaki E., Barati E., Dadgostar E., Aschner M., Tamtaji O.R. (2021). Probiotics and the Treatment of Parkinson’s Disease: An Update. Cell. Mol. Neurobiol..

[372] Divyashri G., Prapulla S., Deol P.K., Sandu S.K. (2022). Animal Models Used for Studying the Benefits of Probiotics in Neurodegeneration. Probiotic Research in Therapeutics: Probiotics in Neurodegenerative Disorders.

[373] Schaly S., Prakash S., Marotta F. (2023). Combating the Sustained Inflammation Involved in Aging and Neurodegenerative Diseases with Probiotics. Gut Microbiota in Aging and Chronic Diseases.

[374] Mintál K., Tóth A., Hormay E., Kovács A., László K., Bufa A., Marosvölgyi T., Kocsis B., Varga A., Vizvári Z. (2022). Novel probiotic treatment of autism spectrum disorder associated social behavioral symptoms in two rodent models. Sci. Rep..

[375] Mitchell L.K., Davies P.S. (2022). Pre-and probiotics in the management of children with autism and gut issues: A review of the current evidence. Eur. J. Clin. Nutr..

[376] Castelli V., d’Angelo M., Lombardi F., Alfonsetti M., Antonosante A., Catanesi M., Benedetti E., Palumbo P., Cifone M.G., Giordano A. (2020). Effects of the probiotic formulation SLAB51 in in vitro and in vivo Parkinson’s disease models. Aging.

[377] Yang X., Yu D., Xue L., Li H., Du J. (2020). Probiotics modulate the microbiota–gut–brain axis and improve memory deficits in aged SAMP8 mice. Acta Pharm. Sin. B.

[378] Jiang J., Chu C., Wu C., Wang C., Zhang C., Li T., Zhai Q., Yu L., Tian F., Chen W. (2021). Efficacy of probiotics in multiple sclerosis: A systematic review of preclinical trials and meta-analysis of randomized controlled trials. Food Funct..

[379] Blacher E., Bashiardes S., Shapiro H., Rothschild D., Mor U., Dori-Bachash M., Kleimeyer C., Moresi C., Harnik Y., Zur M. (2019). Potential roles of gut microbiome and metabolites in modulating ALS in mice. Nature.

[380] Tabouy L., Getselter D., Ziv O., Karpuj M., Tabouy T., Lukic I., Maayouf R., Werbner N., Ben-Amram H., Nuriel-Ohayon M. (2018). Dysbiosis of microbiome and probiotic treatment in a genetic model of autism spectrum disorders. Brain Behav. Immun..

[381] Kong C., Gao R., Yan X., Huang L., Qin H. (2019). Probiotics improve gut microbiota dysbiosis in obese mice fed a high-fat or high-sucrose diet. Nutrition.

[382] Ding Y., Bu F., Chen T., Shi G., Yuan X., Feng Z., Duan Z., Wang R., Zhang S., Wang Q. (2021). A next-generation probiotic: Akkermansia muciniphila ameliorates chronic stress–induced depressive-like behavior in mice by regulating gut microbiota and metabolites. Appl. Microbiol. Biotechnol..

[383] Akbari E., Asemi Z., Daneshvar Kakhaki R., Bahmani F., Kouchaki E., Tamtaji O.R., Hamidi G.A., Salami M. (2016). Effect of Probiotic Supplementation on Cognitive Function and Metabolic Status in Alzheimer’s Disease: A Randomized, Double-Blind and Controlled Trial. Front. Aging Neurosci..

[384] Tamtaji O.R., Heidari-Soureshjani R., Mirhosseini N., Kouchaki E., Bahmani F., Aghadavod E., Tajabadi-Ebrahimi M., Asemi Z. (2019). Probiotic and selenium co-supplementation, and the effects on clinical, metabolic and genetic status in Alzheimer’s disease: A randomized, double-blind, controlled trial. Clin. Nutr..

[385] Hwang Y.-H., Park S., Paik J.-W., Chae S.-W., Kim D.-H., Jeong D.-G., Ha E., Kim M., Hong G., Park S.-H. (2019). Efficacy and Safety of Lactobacillus Plantarum C29-Fermented Soybean (DW2009) in Individuals with Mild Cognitive Impairment: A 12-Week, Multi-Center, Randomized, Double-Blind, Placebo-Controlled Clinical Trial. Nutrients.

[386] Tamtaji O.R., Taghizadeh M., Kakhaki R.D., Kouchaki E., Bahmani F., Borzabadi S., Oryan S., Mafi A., Asemi Z. (2019). Clinical and metabolic response to probiotic administration in people with Parkinson’s disease: A randomized, double-blind, placebo-controlled trial. Clin. Nutr..

[387] Rianda D., Agustina R., Setiawan E., Manikam N. (2019). Effect of probiotic supplementation on cognitive function in children and adolescents: A systematic review of randomised trials. Benef. Microbes.

[388] Pärtty A., Kalliomäki M., Wacklin P., Salminen S., Isolauri E. (2015). A possible link between early probiotic intervention and the risk of neuropsychiatric disorders later in childhood: A randomized trial. Pediatr. Res..

[389] Ng Q.X., Loke W., Venkatanarayanan N., Lim D.Y., Soh A.Y.S., Yeo W.S. (2019). A Systematic Review of the Role of Prebiotics and Probiotics in Autism Spectrum Disorders. Medicina.

[390] Quraishi M.N., Widlak M., Bhala N.a., Moore D., Price M., Sharma N., Iqbal T. (2017). Systematic review with meta-analysis: The efficacy of faecal microbiota transplantation for the treatment of recurrent and refractory Clostridium difficile infection. Aliment. Pharmacol. Ther..

[391] Mullish B.H., Quraishi M.N., Segal J.P., McCune V.L., Baxter M., Marsden G.L., Moore D.J., Colville A., Bhala N., Iqbal T.H. (2018). The use of faecal microbiota transplant as treatment for recurrent or refractory *Clostridium difficile* infection and other potential indications: Joint British Society of Gastroenterology (BSG) and Healthcare Infection Society (HIS) guidelines. Gut.

[392] Ianiro G., Bibbò S., Scaldaferri F., Gasbarrini A., Cammarota G. (2014). Fecal Microbiota Transplantation in Inflammatory Bowel Disease: Beyond the Excitement. Medicine.

[393] Xu D., Chen V.L., Steiner C.A., Berinstein J.A., Eswaran S., Waljee A.K., Higgins P.D., Owyang C. (2019). Efficacy of fecal microbiota transplantation in irritable bowel syndrome: A systematic review and meta-analysis. Am. J. Gastroenterol..

[394] El-Salhy M., Hatlebakk J.G., Gilja O.H., Kristoffersen A.B., Hausken T. (2020). Efficacy of faecal microbiota transplantation for patients with irritable bowel syndrome in a randomised, double-blind, placebo-controlled study. Gut.

[395] Sokol H., Landman C., Seksik P., Berard L., Montil M., Nion-Larmurier I., Bourrier A., Le Gall G., Lalande V., De Rougemont A. (2020). Fecal microbiota transplantation to maintain remission in Crohn’s disease: A pilot randomized controlled study. Microbiome.

[396] Moayyedi P., Surette M.G., Kim P.T., Libertucci J., Wolfe M., Onischi C., Armstrong D., Marshall J.K., Kassam Z., Reinisch W. (2015). Fecal microbiota transplantation induces remission in patients with active ulcerative colitis in a randomized controlled trial. Gastroenterology.

[397] Vrieze A., Van Nood E., Holleman F., Salojärvi J., Kootte R.S., Bartelsman J.F., Dallinga–Thie G.M., Ackermans M.T., Serlie M.J., Oozeer R. (2012). Transfer of intestinal microbiota from lean donors increases insulin sensitivity in individuals with metabolic syndrome. Gastroenterology.

[398] Wu Z., Zhang B., Chen F., Xia R., Zhu D., Chen B., Lin A., Zheng C., Hou D., Li X. (2023). Fecal microbiota transplantation reverses insulin resistance in type 2 diabetes: A randomized, controlled, prospective study. Front. Cell. Infect. Microbiol..

[399] Yu E.W., Gao L., Stastka P., Cheney M.C., Mahabamunuge J., Torres Soto M., Ford C.B., Bryant J.A., Henn M.R., Hohmann E.L. (2020). Fecal microbiota transplantation for the improvement of metabolism in obesity: The FMT-TRIM double-blind placebo-controlled pilot trial. PLoS Med..

[400] Kootte R.S., Levin E., Salojärvi J., Smits L.P., Hartstra A.V., Udayappan S.D., Hermes G., Bouter K.E., Koopen A.M., Holst J.J. (2017). Improvement of insulin sensitivity after lean donor feces in metabolic syndrome is driven by baseline intestinal microbiota composition. Cell Metab..

[401] Wang H., Lu Y., Yan Y., Tian S., Zheng D., Leng D., Wang C., Jiao J., Wang Z., Bai Y. (2020). Promising treatment for type 2 diabetes: Fecal microbiota transplantation reverses insulin resistance and impaired islets. Front. Cell. Infect. Microbiol..

[402] Wang Y., Zhang S., Borody T.J., Zhang F. (2022). Encyclopedia of fecal microbiota transplantation: A review of effectiveness in the treatment of 85 diseases. Chin. Med. J..

[403] Matheson J.-A.T., Holsinger R.M.D. (2023). The Role of Fecal Microbiota Transplantation in the Treatment of Neurodegenerative Diseases: A Review. Int. J. Mol. Sci..

[404] Hazan S. (2020). Rapid improvement in Alzheimer’s disease symptoms following fecal microbiota transplantation: A case report. J. Int. Med. Res..

[405] Park S.-H., Lee J.H., Shin J., Kim J.-S., Cha B., Lee S., Kwon K.S., Shin Y.W., Choi S.H. (2021). Cognitive function improvement after fecal microbiota transplantation in Alzheimer’s dementia patient: A case report. Curr. Med. Res. Opin..

[406] Huang H., Xu H., Luo Q., He J., Li M., Chen H., Tang W., Nie Y., Zhou Y. (2019). Fecal microbiota transplantation to treat Parkinson’s disease with constipation: A case report. Medicine.

[407] Segal A., Zlotnik Y., Moyal-Atias K., Abuhasira R., Ifergane G. (2021). Fecal microbiota transplant as a potential treatment for Parkinson’s disease–A case series. Clin. Neurol. Neurosurg..

[408] Kuai X.-Y., Yao X.-H., Xu L.-J., Zhou Y.-Q., Zhang L.-P., Liu Y., Pei S.-F., Zhou C.-L. (2021). Evaluation of fecal microbiota transplantation in Parkinson’s disease patients with constipation. Microb. Cell Factories.

[409] Borody T., Leis S., Campbell J., Torres M., Nowak A. (2011). Fecal microbiota transplantation (FMT) in multiple sclerosis (MS): 942. Off. J. Am. Coll. Gastroenterol. ACG.

[410] Makkawi S., Camara-Lemarroy C., Metz L. (2018). Fecal microbiota transplantation associated with 10 years of stability in a patient with SPMS. Neurol. -Neuroimmunol. Neuroinflamm..

[411] Engen P.A., Zaferiou A., Rasmussen H., Naqib A., Green S.J., Fogg L.F., Forsyth C.B., Raeisi S., Hamaker B., Keshavarzian A. (2020). Single-Arm, Non-randomized, Time Series, Single-Subject Study of Fecal Microbiota Transplantation in Multiple Sclerosis. Front. Neurol..

[412] Ward L., O’Grady H., Wu K., Cannon K., Workentine M., Louie T. (2016). Combined oral fecal capsules plus fecal enema as treatment of late-onset autism spectrum disorder in children: Report of a small case series. Open Forum Infectious Diseases.

[413] Qureshi F., Adams J., Hanagan K., Kang D.-W., Krajmalnik-Brown R., Hahn J. (2020). Multivariate Analysis of Fecal Metabolites from Children with Autism Spectrum Disorder and Gastrointestinal Symptoms before and after Microbiota Transfer Therapy. J. Pers. Med..

[414] Kang D.-W., Adams J.B., Coleman D.M., Pollard E.L., Maldonado J., McDonough-Means S., Caporaso J.G., Krajmalnik-Brown R. (2019). Long-term benefit of Microbiota Transfer Therapy on autism symptoms and gut microbiota. Sci. Rep..

[415] Park S.Y., Seo G.S. (2021). Fecal Microbiota Transplantation: Is It Safe?. Clin. Endosc..

[416] Wang S., Xu M., Wang W., Cao X., Piao M., Khan S., Yan F., Cao H., Wang B. (2016). Systematic review: Adverse events of fecal microbiota transplantation. PLoS ONE.

[417] Tang R., Li L. (2021). Modulation of short-chain fatty acids as potential therapy method for type 2 diabetes mellitus. Can. J. Infect. Dis. Med. Microbiol..

[418] Breuer R., Soergel K., Lashner B., Christ M., Hanauer S., Vanagunas A., Harig J., Keshavarzian A., Robinson M., Sellin J. (1997). Short chain fatty acid rectal irrigation for left-sided ulcerative colitis: A randomised, placebo controlled trial. Gut.

[419] Xu P., Wang J., Hong F., Wang S., Jin X., Xue T., Jia L., Zhai Y. (2017). Melatonin prevents obesity through modulation of gut microbiota in mice. J. Pineal Res..

[420] Park Y.S., Kim S.H., Park J.W., Kho Y., Seok P.R., Shin J.H., Choi Y.J., Jun J.H., Jung H.C., Kim E.K. (2020). Melatonin in the colon modulates intestinal microbiota in response to stress and sleep deprivation. Intest. Res..

[421] Dias A.M., Cordeiro G., Estevinho M.M., Veiga R., Figueira L., Reina-Couto M., Magro F., the Clinical Pharmacology Unit, São João Hospital University Centre (2020). Gut bacterial microbiome composition and statin intake—A systematic review. Pharmacol. Res. Perspect..

[422] Vieira-Silva S., Falony G., Belda E., Nielsen T., Aron-Wisnewsky J., Chakaroun R., Forslund S.K., Assmann K., Valles-Colomer M., Nguyen T.T.D. (2020). Statin therapy is associated with lower prevalence of gut microbiota dysbiosis. Nature.

[423] Hu X., Li H., Zhao X., Zhou R., Liu H., Sun Y., Fan Y., Shi Y., Qiao S., Liu S. (2021). Multi-omics study reveals that statin therapy is associated with restoration of gut microbiota homeostasis and improvement in outcomes in patients with acute coronary syndrome. Theranostics.

[424] Li D.Y., Li X.S., Chaikijurajai T., Li L., Wang Z., Hazen S.L., Tang W.H.W. (2022). Relation of Statin Use to Gut Microbial Trimethylamine N-Oxide and Cardiovascular Risk. Am. J. Cardiol..

[425] Zhang W., Xu J.-H., Yu T., Chen Q.-K. (2019). Effects of berberine and metformin on intestinal inflammation and gut microbiome composition in db/db mice. Biomed. Pharmacother..

[426] Wu H., Esteve E., Tremaroli V., Khan M.T., Caesar R., Mannerås-Holm L., Ståhlman M., Olsson L.M., Serino M., Planas-Fèlix M. (2017). Metformin alters the gut microbiome of individuals with treatment-naive type 2 diabetes, contributing to the therapeutic effects of the drug. Nat. Med..

[427] Whang A., Nagpal R., Yadav H. (2019). Bi-directional drug-microbiome interactions of anti-diabetics. EBioMedicine.

[428] Zhang B., Liu K., Yang H., Jin Z., Ding Q., Zhao L. (2022). Gut Microbiota: The Potential Key Target of TCM’s Therapeutic Effect of Treating Different Diseases Using the Same Method—UC and T2DM as Examples. Front. Cell. Infect. Microbiol..

[429] Fortea M., Albert-Bayo M., Abril-Gil M., Ganda Mall J.-P., Serra-Ruiz X., Henao-Paez A., Expósito E., González-Castro A.M., Guagnozzi D., Lobo B. (2021). Present and Future Therapeutic Approaches to Barrier Dysfunction. Front. Nutr..

[430] Tian J., Bai B., Gao Z., Yang Y., Wu H., Wang X., Wang J., Li M., Tong X. (2021). Alleviation Effects of GQD, a Traditional Chinese Medicine Formula, on Diabetes Rats Linked to Modulation of the Gut Microbiome. Front. Cell. Infect. Microbiol..

[431] Hu W., Huang L., Zhou Z., Yin L., Tang J. (2022). Diallyl Disulfide (DADS) Ameliorates Intestinal Candida albicans Infection by Modulating the Gut microbiota and Metabolites and Providing Intestinal Protection in Mice. Front. Cell. Infect. Microbiol..

[432] Xie X., Liao J., Ai Y., Gao J., Zhao J., Qu F., Xu C., Zhang Z., Wen W., Cui H. (2021). Pi-Dan-Jian-Qing Decoction Ameliorates Type 2 Diabetes Mellitus Through Regulating the Gut Microbiota and Serum Metabolism. Front. Cell. Infect. Microbiol..

[433] Yin R., Liu S., Jiang X., Zhang X., Wei F., Hu J. (2022). The Qingchangligan Formula Alleviates Acute Liver Failure by Regulating Galactose Metabolism and Gut Microbiota. Front. Cell. Infect. Microbiol..

[434] Tang R., Yi J., Lu S., Chen B., Liu B. (2022). Therapeutic Effect of Buyang Huanwu Decoction on the Gut Microbiota and Hippocampal Metabolism in a Rat Model of Cerebral Ischemia. Front. Cell. Infect. Microbiol..

[435] Yu S., Jiang J., Li Q., Liu X., Wang Z., Yang L., Ding L. (2022). Schisantherin A alleviates non-alcoholic fatty liver disease by restoring intestinal barrier function. Front. Cell. Infect. Microbiol..

[436] Huang J.-Q., Wei S.-Y., Cheng N., Zhong Y.-B., Yu F.-H., Li M.-D., Liu D.-Y., Li S.-S., Zhao H.-M. (2022). Chimonanthus nitens Oliv. Leaf Granule Ameliorates DSS-Induced Acute Colitis Through Treg Cell Improvement, Oxidative Stress Reduction, and Gut Microflora Modulation. Front. Cell. Infect. Microbiol..

[437] Huang Y., Zheng Y., Yang F., Feng Y., Xu K., Wu J., Qu S., Yu Z., Fan F., Huang L. (2022). Lycium barbarum Glycopeptide prevents the development and progression of acute colitis by regulating the composition and diversity of the gut microbiota in mice. Front. Cell. Infect. Microbiol..

[438] Zhao H., Chen R., Zheng D., Xiong F., Jia F., Liu J., Zhang L., Zhang N., Zhu S., Liu Y. (2022). Modified Banxia Xiexin Decoction Ameliorates Polycystic Ovarian Syndrome With Insulin Resistance by Regulating Intestinal Microbiota. Front. Cell. Infect. Microbiol..

[439] Wu G., Zhang W., Zheng N., Zu X., Tian S., Zhong J., Zhang Y., Liao J., Sheng L., Ge G. (2022). Integrated microbiome and metabolome analysis reveals the potential therapeutic mechanism of Qing-Fei-Pai-Du decoction in mice with coronavirus-induced pneumonia. Front. Cell. Infect. Microbiol..

[440] Li Y., Kang Y., Du Y., Chen M., Guo L., Huang X., Li T., Chen S., Yang F., Yu F. (2022). Effects of Konjaku Flour on the Gut Microbiota of Obese Patients. Front. Cell. Infect. Microbiol..

[441] Guo Q., Ni C., Li L., Li M., Jiang X., Gao L., Zhu H., Song J. (2022). Integrated Traditional Chinese Medicine Improves Functional Outcome in Acute Ischemic Stroke: From Clinic to Mechanism Exploration With Gut Microbiota. Front. Cell. Infect. Microbiol..

[442] Jabczyk M., Nowak J., Hudzik B., Zubelewicz-Szkodzińska B. (2021). Curcumin and Its Potential Impact on Microbiota. Nutrients.

[443] Cui C., Han Y., Li H., Yu H., Zhang B., Li G. (2022). Curcumin-driven reprogramming of the gut microbiota and metabolome ameliorates motor deficits and neuroinflammation in a mouse model of Parkinson’s disease. Front. Cell. Infect. Microbiol..

[444] Chen Z., Zhang Z., Liu J., Qi H., Li J., Chen J., Huang Q., Liu Q., Mi J., Li X. (2022). Gut Microbiota: Therapeutic Targets of Ginseng Against Multiple Disorders and Ginsenoside Transformation. Front. Cell. Infect. Microbiol..

[445] Jeon H., Kim H.-Y., Bae C.-H., Lee Y., Kim S. (2020). Korean Red ginseng regulates intestinal tight junction and inflammation in the colon of a Parkinson’s disease mouse model. J. Med. Food.

[446] Xiong W., Zhao X., Xu Q., Wei G., Zhang L., Fan Y., Wen L., Liu Y., Zhang T., Zhang L. (2022). Qisheng Wan formula ameliorates cognitive impairment of Alzheimer’s disease rat via inflammation inhibition and intestinal microbiota regulation. J. Ethnopharmacol..

[447] Wang L., Lu J., Zeng Y., Guo Y., Wu C., Zhao H., Zheng H., Jiao J. (2020). Improving Alzheimer’s disease by altering gut microbiota in tree shrews with ginsenoside Rg1. FEMS Microbiol. Lett..

[448] Dong C., Yu J., Yang Y., Zhang F., Su W., Fan Q., Wu C., Wu S. (2021). Berberine, a potential prebiotic to indirectly promote Akkermansia growth through stimulating gut mucin secretion. Biomed. Pharmacother..

[449] Wang H., Zhang H., Gao Z., Zhang Q., Gu C. (2022). The mechanism of berberine alleviating metabolic disorder based on gut microbiome. Front. Cell. Infect. Microbiol..

[450] Bao C., Wu L., Wang D., Chen L., Jin X., Shi Y., Li G., Zhang J., Zeng X., Chen J. (2022). Acupuncture improves the symptoms, intestinal microbiota, and inflammation of patients with mild to moderate Crohn’s disease: A randomized controlled trial. eClinicalMedicine.

[451] Bao C., Wu L., Shi Y., Wu H., Liu H., Zhang R., Yu L., Wang J. (2011). Moxibustion down-regulates colonic epithelial cell apoptosis and repairs tight junctions in rats with Crohn’s disease. World J. Gastroenterol..

[452] Wei D., Xie L., Zhuang Z., Zhao N., Huang B., Tang Y., Yu S., Zhou Q., Wu Q. (2019). Gut microbiota: A new strategy to study the mechanism of electroacupuncture and moxibustion in treating ulcerative colitis. Evid.-Based Complement. Altern. Med..

[453] Qi Q., Liu Y.-N., Jin X.-M., Zhang L.-S., Wang C., Bao C.-H., Liu H.-R., Wu H.-G., Wang X.-M. (2018). Moxibustion treatment modulates the gut microbiota and immune function in a dextran sulphate sodium-induced colitis rat model. World J. Gastroenterol..

[454] Qi Q., Liu Y.-N., Lv S.-Y., Wu H.-G., Zhang L.-S., Cao Z., Liu H.-R., Wang X.-M., Wu L.-Y. (2022). Gut microbiome alterations in colitis rats after moxibustion at bilateral Tianshu acupoints. BMC Gastroenterol..

[455] Wang X., Qi Q., Wang Y., Wu H., Jin X., Yao H., Jin D., Liu Y., Wang C. (2018). Gut microbiota was modulated by moxibustion stimulation in rats with irritable bowel syndrome. Chin. Med..

[456] Bao C.H., Wang C.Y., Li G.N., Yan Y.L., Wang D., Jin X.M., Wu L.Y., Liu H.R., Wang X.M., Shi Z. (2019). Effect of mild moxibustion on intestinal microbiota and NLRP6 inflammasome signaling in rats with post-inflammatory irritable bowel syndrome. World J. Gastroenterol..

[457] Jang J.-H., Yeom M.-J., Ahn S., Oh J.-Y., Ji S., Kim T.-H., Park H.-J. (2020). Acupuncture inhibits neuroinflammation and gut microbial dysbiosis in a mouse model of Parkinson’s disease. Brain Behav. Immun..

[458] Wang Q., Wang Y., Liu Z., Guo J., Li J., Zhao Y. (2022). Improvement effect of acupuncture on locomotor function in Parkinson disease via regulating gut microbiota and inhibiting inflammatory factor release. J. Acupunct. Tuina Sci..

[459] Nazarova L., Liu H., Xie H., Wang L., Ding H., An H., Huang D. (2022). Targeting gut-brain axis through scalp-abdominal electroacupuncture in Parkinson’s disease. Brain Res..

[460] Yang B., He M., Chen X., Sun M., Pan T., Xu X., Zhang X., Gong Q., Zhao Y., Jin Z. (2022). Acupuncture effect assessment in APP/PS1 transgenic mice: On regulating learning-memory abilities, gut microbiota, and microbial metabolites. Comput. Math. Methods Med..

[461] Pham V.T., Dold S., Rehman A., Bird J.K., Steinert R.E. (2021). Vitamins, the gut microbiome and gastrointestinal health in humans. Nutr. Res..

[462] Francino M. (2016). Antibiotics and the human gut microbiome: Dysbioses and accumulation of resistances. Front. Microbiol..

[463] Jernberg C., Lofmark S., Edlund C., Jansson J.K. (2010). Long-term impacts of antibiotic exposure on the human intestinal microbiota. Microbiology.

[464] Ianiro G., Tilg H., Gasbarrini A. (2016). Antibiotics as deep modulators of gut microbiota: Between good and evil. Gut.

[465] Stewardson A.J., Gaïa N., Francois P., Malhotra-Kumar S., Delemont C., de Tejada B.M., Schrenzel J., Harbarth S., Lazarevic V., WP S. (2015). Collateral damage from oral ciprofloxacin versus nitrofurantoin in outpatients with urinary tract infections: A culture-free analysis of gut microbiota. Clin. Microbiol. Infect..

[466] Vervoort J., Xavier B.B., Stewardson A., Coenen S., Godycki-Cwirko M., Adriaenssens N., Kowalczyk A., Lammens C., Harbarth S., Goossens H. (2015). Metagenomic analysis of the impact of nitrofurantoin treatment on the human faecal microbiota. J. Antimicrob. Chemother..

[467] Ponziani F.R., Zocco M.A., D’Aversa F., Pompili M., Gasbarrini A. (2017). Eubiotic properties of rifaximin: Disruption of the traditional concepts in gut microbiota modulation. World J. Gastroenterol..

[468] Li H., Xiang Y., Zhu Z., Wang W., Jiang Z., Zhao M., Cheng S., Pan F., Liu D., Ho R.C.M. (2021). Rifaximin-mediated gut microbiota regulation modulates the function of microglia and protects against CUMS-induced depression-like behaviors in adolescent rat. J. Neuroinflamm..

[469] Rao S.S.C., Bhagatwala J. (2019). Small Intestinal Bacterial Overgrowth: Clinical Features and Therapeutic Management. Clin. Transl. Gastroenterol..

[470] Sroka N., Rydzewska-Rosołowska A., Kakareko K., Rosołowski M., Głowińska I., Hryszko T. (2023). Show Me What You Have Inside—The Complex Interplay between SIBO and Multiple Medical Conditions—A Systematic Review. Nutrients.

[471] Prantera C., Lochs H., Grimaldi M., Danese S., Scribano M.L., Gionchetti P. (2012). Rifaximin-Extended Intestinal Release Induces Remission in Patients With Moderately Active Crohn’s Disease. Gastroenterology.

[472] Hong C.-T., Chan L., Chen K.-Y., Lee H.-H., Huang L.-K., Yang Y.-C.S., Liu Y.-R., Hu C.-J. (2022). Rifaximin Modifies Gut Microbiota and Attenuates Inflammation in Parkinson’s Disease: Preclinical and Clinical Studies. Cells.

[473] Suhocki P.V., Ronald J.S., Diehl A.M.E., Murdoch D.M., Doraiswamy P.M. (2022). Probing gut-brain links in Alzheimer’s disease with rifaximin. Alzheimers Dement.

[474] Sandler R.H., Finegold S.M., Bolte E.R., Buchanan C.P., Maxwell A.P., Väisänen M.-L., Nelson M.N., Wexler H.M. (2000). Short-term benefit from oral vancomycin treatment of regressive-onset autism. J. Child Neurol..

[475] Obrenovich M., Jaworski H., Tadimalla T., Mistry A., Sykes L., Perry G., Bonomo R.A. (2020). The role of the microbiota–gut–brain axis and antibiotics in ALS and neurodegenerative diseases. Microorganisms.

[476] Melzer N., Meuth S.G., Torres-Salazar D., Bittner S., Zozulya A.L., Weidenfeller C., Kotsiari A., Stangel M., Fahlke C., Wiendl H. (2008). A β-Lactam Antibiotic Dampens Excitotoxic Inflammatory CNS Damage in a Mouse Model of Multiple Sclerosis. PLoS ONE.

[477] Weng J.-C., Tikhonova M.A., Chen J.-H., Shen M.-S., Meng W.-Y., Chang Y.-T., Chen K.-H., Liang K.-C., Hung C.-S., Amstislavskaya T.G. (2016). Ceftriaxone prevents the neurodegeneration and decreased neurogenesis seen in a Parkinson’s disease rat model: An immunohistochemical and MRI study. Behav. Brain Res..

[478] Kumari S., Deshmukh R. (2021). β-lactam antibiotics to tame down molecular pathways of Alzheimer’s disease. Eur. J. Pharmacol..

[479] Tai C.-H., Bellesi M., Chen A.-C., Lin C.-L., Li H.-H., Lin P.-J., Liao W.-C., Hung C.-S., Schwarting R.K., Ho Y.-J. (2019). A new avenue for treating neuronal diseases: Ceftriaxone, an old antibiotic demonstrating behavioral neuronal effects. Behav. Brain Res..

[480] Tikhonova M.A., Ho S.-C., Akopyan A.A., Kolosova N.G., Weng J.-C., Meng W.-Y., Lin C.-L., Amstislavskaya T.G., Ho Y.-J. (2017). Neuroprotective effects of ceftriaxone treatment on cognitive and neuronal deficits in a rat model of accelerated senescence. Behav. Brain Res..

[481] Hamblin M.R. (2018). Mechanisms and Mitochondrial Redox Signaling in Photobiomodulation. Photochem. Photobiol..

[482] Benson P., Kim J.Y., Riveros C., Camp A., Johnstone D.M. (2020). Elucidating the time course of the transcriptomic response to photobiomodulation through gene co-expression analysis. J. Photochem. Photobiol. B Biol..

[483] Chow R.T., Johnson M.I., Lopes-Martins R.A., Bjordal J.M. (2009). Efficacy of low-level laser therapy in the management of neck pain: A systematic review and meta-analysis of randomised placebo or active-treatment controlled trials. Lancet.

[484] Chow R.T., David M.A., Armati P.J. (2007). 830 nm laser irradiation induces varicosity formation, reduces mitochondrial membrane potential and blocks fast axonal flow in small and medium diameter rat dorsal root ganglion neurons: Implications for the analgesic effects of 830 nm laser. J. Peripher. Nerv. Syst..

[485] Hamblin M.R. (2017). Mechanisms and applications of the anti-inflammatory effects of photobiomodulation. AIMS Biophys..

[486] El Massri N., Johnstone D.M., Peoples C.L., Moro C., Reinhart F., Torres N., Stone J., Benabid A.-L., Mitrofanis J. (2016). The effect of different doses of near infrared light on dopaminergic cell survival and gliosis in MPTP-treated mice. Int. J. Neurosci..

[487] El Massri N., Mitrofanis J., Hamblin M.R., Huang Y.-Y. (2019). The experimental evidence for photobiomodulation-induced cellular and behavioral changes in animal models of Parkinson’s disease: A template for translation to patients. Photobiomodulation in the Brain.

[488] El Massri N., Moro C., Torres N., Darlot F., Agay D., Chabrol C., Johnstone D.M., Stone J., Benabid A.-L., Mitrofanis J. (2016). Near-infrared light treatment reduces astrogliosis in MPTP-treated monkeys. Exp. Brain Res..

[489] Ganeshan V., Skladnev N.V., Kim J.Y., Mitrofanis J., Stone J., Johnstone D.M. (2019). Pre-conditioning with remote photobiomodulation modulates the brain transcriptome and protects against MPTP insult in mice. Neuroscience.

[490] Johnstone D.M., el Massri N., Moro C., Spana S., Wang X.S., Torres N., Chabrol C., De Jaeger X., Reinhart F., Purushothuman S. (2014). Indirect application of near infrared light induces neuroprotection in a mouse model of parkinsonism—An abscopal neuroprotective effect. Neuroscience.

[491] Johnstone D.M., Mitrofanis J., Stone J. (2015). Targeting the body to protect the brain: Inducing neuroprotection with remotely-applied near infrared light. Neural Regen. Res..

[492] Stone J., Johnstone D., Mitrofanis J. The helmet experiment in Parkinson’s disease: An observation of the mechanism of neuroprotection by near infra-red light. Proceedings of the 9th WALT Congress.

[493] Wang M., Cao J., Amakye W.K., Gong C., Li Q., Ren J. (2020). Mid infrared light treatment attenuates cognitive decline and alters the gut microbiota community in APP/PS1 mouse model. Biochem. Biophys. Res. Commun..

[494] Chen Q., Jinpeng W.U., Dong X., Yin H., Shi X., Siying S.U., Che B., Yingxin L.I., Yang J. (2021). Gut flora-targeted photobiomodulation therapy improves senile dementia in an Aß-induced Alzheimer’s disease animal model. J. Photochem. Photobiol. B Biol..

[495] Roubaud Baudron C., Letenneur L., Langlais A., Buissonnière A., Mégraud F., Dartigues J.-F., Salles N., Personnes Agées QUID Study (2013). Does *Helicobacter pylori* Infection Increase Incidence of Dementia? The Personnes Agées QUID Study. J. Am. Geriatr. Soc..

[496] Lu Y., Yang J., Dong C., Fu Y., Liu H. (2021). Gut microbiome-mediated changes in bone metabolism upon infrared light exposure in rats. J. Photochem. Photobiol. B Biol..

[497] Min S.H., Kwon J., Do E.-J., Kim S.H., Kim E.S., Jeong J.-Y., Bae S.M., Kim S.-Y., Park D.H. (2022). Duodenal Dual-Wavelength Photobiomodulation Improves Hyperglycemia and Hepatic Parameters with Alteration of Gut Microbiome in Type 2 Diabetes Animal Model. Cells.

[498] Bicknell B., Liebert A., McLachlan C.S., Kiat H. (2022). Microbiome Changes in Humans with Parkinson’s Disease after Photobiomodulation Therapy: A Retrospective Study. J. Pers. Med..

[499] Liebert A., Bicknell B., Laakso E.L., Heller G., Jalilitabaei P., Tilley S., Mitrofanis J., Kiat H. (2021). Improvements in clinical signs of Parkinson’s disease using photobiomodulation: A prospective proof-of-concept study. BMC Neurol..

[500] Liebert A., Bicknell B., Laakso E.-L., Jalilitabaei P., Tilley S., Kiat H., Mitrofanis J. (2022). Remote Photobiomodulation Treatment for the Clinical Signs of Parkinson’s Disease: A Case Series Conducted During COVID-19. Photobiomodul. Photomed. Laser Surg..

[501] Cardoso F.D.S., Gonzalez-Lima F., Coimbra N.C. (2022). Mitochondrial Photobiomodulation as a Neurotherapeutic Strategy for Epilepsy. Front. Neurol..

[502] Liebert A.D., Bicknell B. (2017). Clinical trial involving sufferers and non-sufferers of cervicogenic headache (CGH): Potential mechanisms of action of photobiomodulation. Mechanisms of Photobiomodulation Therapy XII.

[503] Bian J., Liebert A., Bicknell B., Chen X.-M., Huang C., Pollock C.A. (2022). Therapeutic potential of photobiomodulation for chronic kidney disease. Int. J. Mol. Sci..

[504] Caldieraro M.A., Cassano P. (2019). Transcranial and systemic photobiomodulation for major depressive disorder: A systematic review of efficacy, tolerability and biological mechanisms. J. Affect. Disord..

[505] Tolentino M., Cho C.C., Lyons J.-A. (2019). Photobiomodulation therapy (PBMT) regulates the production of IL-10 and IFN-Ɣ by peripheral blood mononuclear cells (PBMC) and CD4+ T cells isolated from subjects with Multiple Sclerosis (MS). J. Immunol..

[506] Ceranoglu T.A., Cassano P., Hoskova B., Green A., Dallenbach N., DiSalvo M., Biederman J., Joshi G. (2022). Transcranial photobiomodulation in adults with high-functioning autism spectrum disorder: Positive findings from a proof-of-concept study. Photobiomodul. Photomed. Laser Surg..

[507] Bowen R., Arany P.R. (2023). Use of either Transcranial or Whole-Body Photobiomodulation Treatments improves COVID-19 Brain Fog. J. Biophotonics.

[508] Naeser M.A., Ho M.D., Martin P.I., Hamblin M.R., Koo B.-B. (2020). Increased functional connectivity within intrinsic neural networks in chronic stroke following treatment with red/near-infrared transcranial photobiomodulation: Case series with improved naming in aphasia. Photobiomodul. Photomed. Laser Surg..

[509] Salehpour F., Khademi M., Hamblin M.R. (2021). Photobiomodulation Therapy for Dementia: A Systematic Review of Pre-Clinical and Clinical Studies. J. Alzheimer’s Dis..

[510] Salehpour F., Gholipour-Khalili S., Farajdokht F., Kamari F., Walski T., Hamblin M.R., DiDuro J.O., Cassano P. (2020). Therapeutic potential of intranasal photobiomodulation therapy for neurological and neuropsychiatric disorders: A narrative review. Rev. Neurosci..

[511] Stevens A.R., Hadis M., Milward M., Ahmed Z., Belli A., Palin W., Davies D.J. (2023). Photobiomodulation in acute traumatic brain injury: A systematic review and meta-analysis. J. Neurotrauma.

